# Shaping of olfactory responses by taste in a new assay for operant learning in *Drosophila melanogaster*

**DOI:** 10.1242/jeb.251074

**Published:** 2025-12-19

**Authors:** John S. Hernandez, Nelson Le, Rebecca Oramas, Reza Azanchi, Nicholas J. Mei, Abigail T. Long, Maggie R. Robertson, Oliver M. Cook, Andrew M. Dacks, Karla R. Kaun

**Affiliations:** ^1^ Department of Neuroscience, Brown University, Providence, RI, 02912, USA; ^2^ Post-baccalaureate Research Education Program, Brown University, Providence, RI, 02912, USA; ^3^ Neuroscience Graduate Program, Brown University, Providence, RI, 02912, USA; ^4^ Department of Biology, West Virginia University, Morgantown, WV, 26506, USA

**Keywords:** *Drosophila*, Operant behavior, Motivation, Learning, Multisensory, Olfaction, Taste

## Abstract

Animals are driven to maximize food rewards and adjust their behavior to seek high-quality food and avoid low-quality options. This holds true for *Drosophila melanogaster*, which approaches food-associated odors and tastes while avoiding aversive ones. Despite its importance for understanding motivation, how voluntary olfactory and gustatory experiences shape fly interactions with these stimuli over time remains unclear. Here, we investigate how stimuli shape volitional behavior using our novel operant learning assay (Open-LA), which tracks individual flies as they enter/exit a region of stimuli self-administration. We analyzed the behaviors flies demonstrated when they control access to an aversive or appetitive odor or taste and analyzed how these behaviors were shaped by experience. As predicted, flies pursued apple cider vinegar and avoided benzaldehyde and showed rapid operant learning for both odors. Flies also self-administered both simulated sweet and bitter taste, which slightly altered aversive odor responses, but did not strongly enhance odor-based operant learning. These data suggest olfaction is the primary sense guiding volitional behaviors and provides a behavioral framework for examining how animals pursue positive and avoid negative stimuli.

## Teaser

**Summary:** A new assay that characterizes subtle behaviors and assesses operant learning in freely behaving *Drosophila* reveals that olfaction more strongly influences stimulus-seeking behavior than taste.

## INTRODUCTION

Survival in a complex environment depends on an animal's ability to associate a stimulus with its consequence (classical conditioning; Pavlov, 1960) or to adapt its behavior based on past outcomes (operant conditioning; Skinner, 1938). By learning from their behaviors’ outcomes, animals can gain environmental control, adjusting subsequent actions to alter the likelihood of reward or punishment (Toates, 2012).

Animals use olfactory and taste cues to navigate their environment (Quinn et al., 1974) and assess positive and negative food reinforcers (Boesveldt and de Graaf, 2017; Reisenman and Scott, 2019). Like other animals, *Drosophila melanogaster* maximizes fitness by balancing the drive to seek rewards with the drive to avoid danger (Álvarez-Salvado et al., 2018; Budick and Dickinson, 2006; Demir et al., 2020; Jayaram et al., 2023; Lowet et al., 2020; Matheson et al., 2022; van Breugel and Dickinson, 2014). They also use both classical and operant conditioning strategies to adapt their behavior based on their past and current circumstances (Rajagopalan et al., 2023; Wiggin et al., 2021).

Flies can learn to approach an odor paired with sugar reward (Tempel et al., 1983) and will avoid odors paired with a noxious experience like shock or a bitter substance using classical conditioning strategies (Quinn et al., 1974; Tully and Quinn, 1985). Odors act as cues that attract or repel flies at a distance from their source, whereas tastes provide immediate localized feedback on whether the substance emitting the odor is nutritious/detrimental. Although both taste (Gerber et al., 2009; Masek and Keene, 2016; Scott, 2005) and smell (Álvarez-Salvado et al., 2018; Budick and Dickinson, 2006; Gaudry et al., 2012; Hung and Stopfer, 2018; Steck et al., 2012; van Breugel and Dickinson, 2014) are key for reinforcing behavior, they are typically studied in isolation and in an involuntary context. How taste modulates operant behaviors upon odor exposure, and how this integrated behavioral output changes with experience, remains poorly understood.

Previous work has demonstrated that *Drosophila* can operantly respond to appetitive and aversive stimuli. Flies make robust operant motor decisions to avoid electric shock (Murphey, 1967; Platt et al., 1980; Zars et al., 2000) and intense heat (Wolf and Heisenberg, 1991). In contrast, operant behaviors in response to rewards appear to be strongest when an ethologically related cue predicts rewards. For example, activation of sweet receptors induces local search in a virtual environment, but is insufficient to induce association with a visual stimulus (Haberkern et al., 2019). Flies can also learn to turn left or right for a sucrose reward in a Y-maze, but such data are highly variable and a pattern emerges only in well-rested flies (Wiggin et al., 2021). However, flies readily learn to turn left or right toward an odor that predicts an optogenetically induced sweet taste, instead of one that does not predict a gustatory stimulus (Rajagopalan et al., 2023). Although these findings demonstrate flies operantly respond, they do not capture the complexity of individual fly behavior when freely interacting with reward-predicting cues. To capture this complexity, it is necessary to assess the dynamic behavioral strategies flies employ as they learn to engage with a reward or punishment, as opposed to measuring just the outcome of accessing the reward.

To overcome these limitations and enhance our understanding of motivated decisions, we developed a novel automated operant learning assay (Open-LA). This high-throughput, self-paced assay simultaneously tracks multiple flies in individual arenas for a 15 min session as they associate regions of an arena with an odor, a simulated taste, or a combination of odor and simulated taste. Tracking flies while they self-administer these stimuli allows characterization of multiple behaviors that are shaped by motivational stimuli over time. The Open-LA thus quantifies operant learning by providing measures of how the frequency of a behavior is determined by its consequences (e.g. punishers, reinforcers; Murphy and Lupfer, 2014; Skinner, 1938).

To identify the complexity of reinforced behaviors during operant learning, we analyzed multiple metrics of behavior in response to repeated self-exposures to an aversive or appetitive odor and/or taste. We first evaluated total stimulus administration time, total distance moved during administration, and closest proximity to the delivery port. Since these acted as a benchmark to determine the overall outcome of a training session, we collectively termed these ‘session behaviors’. We also analyzed stimulus administration frequency, stimulation duration, distance moved per stimulation and latency to re-stimulate. Since these were periodic behaviors that occur repeatedly over the length of a training session, we defined them as ‘bout behaviors’. Using these analyses, we provide a rigorous characterization of the process of operant learning by assessing how reward and punishment influence stimulus pursuit and avoidance, and how these volitional behaviors change with repeated experience.

By analyzing both session and bout behaviors in response to an appetitive (apple cider vinegar) or aversive (benzaldehyde) odor, and optogenetic simulation of a sweet (Gr64a) or bitter (Gr66a) taste, this work provides a more advanced understanding of how behavior is affected by odor and taste, uncovering biologically relevant nuances. For instance, these data revealed that olfaction is the primary sense guiding volitional behaviors, while gustatory neuron activation in sated flies minimally influences operant learning for both reward seeking and punishment avoidance. Moreover, this detailed behavioral characterization can serve as a technical foundation for future *Drosophila* studies to thoroughly examine how different stimuli combinations, environmental factors and internal states (e.g. disease, reproductive, metabolic) can impact volitional behavior and operant learning.

## MATERIALS AND METHODS

### Fly stocks and husbandry

Virgin female and virgin male wild-type *Canton-S (w+CS)* flies were reared on standard cornmeal-agar food supplemented with the antifungal agent tegosept, maintained at 24°C with 70% humidity, and kept on a 14 h:10 h light:dark cycle. First-generation flies were collected following a brief CO_2_ exposure and grouped in separate vials containing 10 flies each (housing females and males separately) 3 days before experimentation. Flies used in experiments were 3–4 days old at the start of the experiment. Flies were not food-deprived during the experiments.

For taste experiments, female and male *Gr64f-GAL4;20XUAS-IVS-CsChrimson-mCherry* and *Gr66a-GAL4;20XUAS-IVS-CsChrimson-mCherry* were used (gift from Gilad Barnea at Brown University), which had a *w[1118]* genetic background. Information about the *Gr64f-GAL4.9.7* (Dahanukar et al., 2007) FlyBase ID FBti0162679), *Gr66a-GAL4.D* (Wang et al., 2004; FlyBase ID FBti0126845) and 20XUAS-IVS-CsChrimson-mCherry (Jovanic et al., 2016) lines has been published.

### Operant learning assay (Open-LA) apparatus

**Fig. 1.**
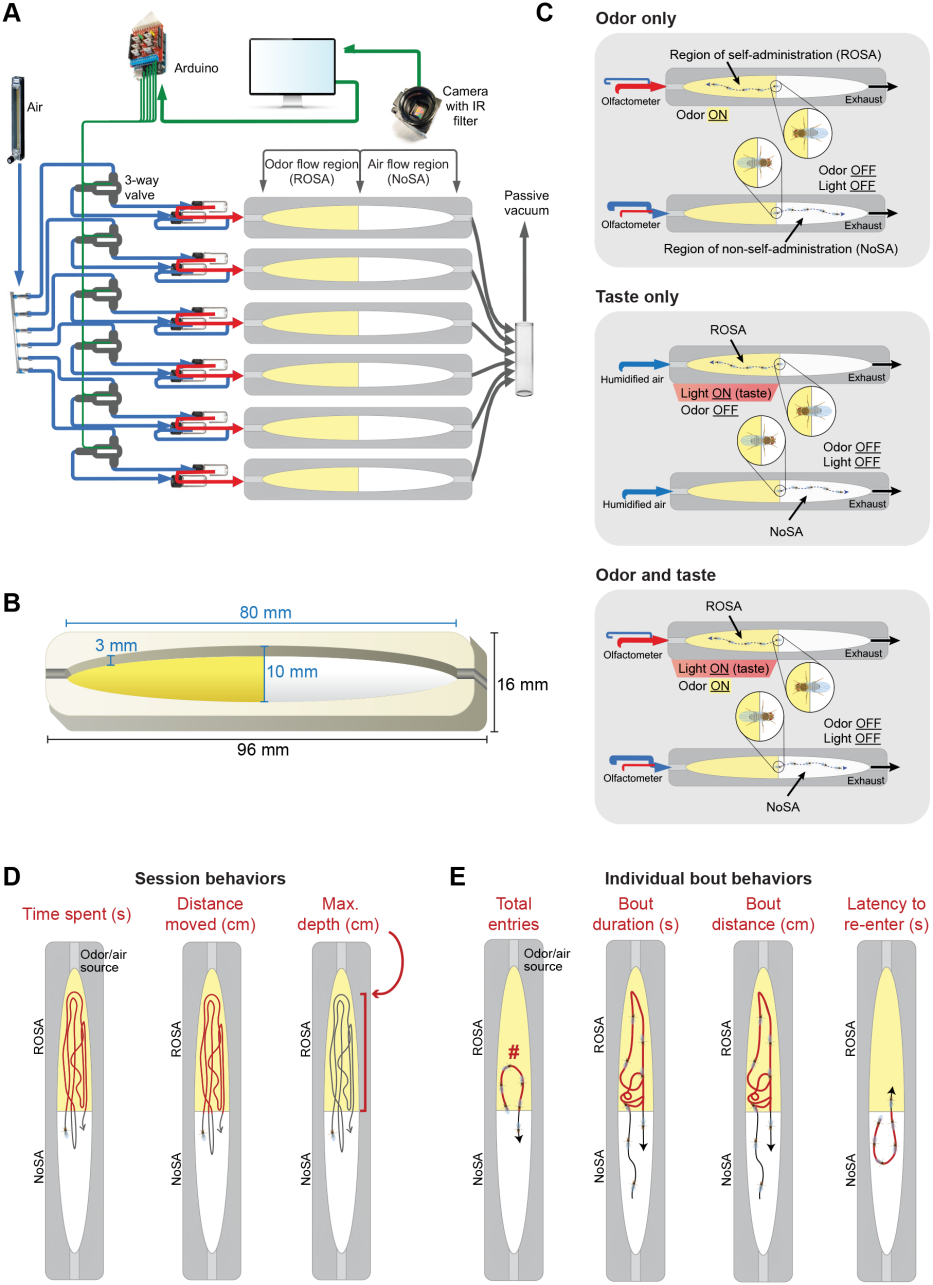
**Open-LA apparatus, arena dimensions, stimulus overview and categories of behaviors observed.** (A) Six operant arenas with Arduino-controlled air, odor and LED lights. (B) Arena measurements. (C) Within the arena, the fly learns to self-administer odors (top), activate taste CsChrimson-expressing neurons via red light (middle) or both (bottom), by entering the region of self-administration (ROSA). To terminate stimulus exposure, they enter the region of non-self administration (NoSA). (D) Illustrations depicting session behaviors: time spent in ROSA, distance moved in ROSA, and closeness to the odor/air delivery port (depth) across the 15 min tracking session. (E) Illustrations depicting bout behaviors: total entries (number of bouts) into ROSA, bout duration, bout distance and latency to re-enter ROSA after exiting.

The Open-LA apparatus consists of six custom-made oblong arenas (in-house laser cut), each equipped with infrared LEDs beneath them and placed inside a temperature- and humidity-controlled box (Fig. 1A,B, Fig. S1). The box protects the arenas from external light and has an internal bulb located in the upper corner for fly vision (Fig. S1). A USB camera is integrated with Python code and linked to an Arduino, which controls flow choice using three-way solenoid valves (411L2312HVS, ASCO, Florham Park, NJ, USA) (Fig. 1A, Fig. S1). Yellow tape was placed below half of the chamber, demarcating the region of self-administration (ROSA) and the non-taped side where flies could experience humidified air (non-self-administration region: NoSA; Fig. S1A,C). In air-only experiments, flies entering ROSA experienced humidified air regardless of location.

A high-resolution tracking software was used to record the fly's position (*x*,*y*) at 15 fps over 15 min, while the flies chose to avoid or pursue the stimuli. Automated tracking and behavior analysis algorithms were used to generate behavioral output datasets from video recordings. Video recordings were made using a USB camera (USBFHD01M-L21, ELP, China) positioned approximately 16 cm above the arenas using a camera stand (80/20 Extrusion rail, Amazon, Seattle, WA, USA). The camera was fitted with a 6 mm focal length, F2.0, M12 adaptor lens for 1/3″ (8.5 mm) image sensors (PT-0620, M12 Lenses, Maitland, FL, USA). The lens was fitted with a low-wavelength pass (IR) light filter [Edmund Optics, 54-664 (RG-850), Barrington, NJ, USA] to prevent ambient light and optogenetic stimulation from negatively affecting the accuracy of IR tracking. Our high-resolution tracking data output is also suitable for analysis using open-source software such as Ctrax (Branson et al., 2009) and JAABA (Kabra et al., 2013) to obtain additional behavioral outputs. Although analysis using these systems was not performed in this manuscript, we provide access to our raw tracking videos for further analysis (available to download at https://doi.org/10.26300/ghx7-a039).

The hardware used in this experiment was controlled by a Python script (https://github.com/kaun-lab). This script controlled which of the two glass vials (CAS 60958A912 and CAS 60942A40, Fisher Scientific, Waltham, MA, USA) containing odors or water associated with each lane provided the airflow passing through each arena (Fig. 1A) via PTFE tubing [1/16″ ID, 1/8″ OD (1.6 mm, 3.2 mm); MFLX96000-02, VWR, Radnor, PA, USA]. Airflow was provided by a Whisper Air Pump (AP-300, Amazon) via Teflon tubing [1/4″ (6.4 mm) OD, Home Depot, Atlanta, GA, USA] and a Teflon six-way manifold air distributor/mixer (N-Research, West Caldwell, NJ, USA), which were controlled by a flowmeter (MFLX03217-20, VWR). For each arena on each day (i.e. every time the script was run), a .csv file was generated, which included the fly position in *x*- and *y*-coordinates, as well as a Boolean Occupation Score indicating if the fly was in the user-defined region of interest for each frame. When flies entered the ROSA, the Boolean Occupation Score was 1, and when the flies entered the NoSA the Boolean Occupation Score was 0. For a given time bin (e.g. 30 s or 1 min), the total number of 1s was summed across all frames and divided by the total number of frames to produce an averaged Boolean Occupation Score.

### Odor stimuli

Air flow was maintained via a three-way solenoid valve (ASCO valves, St Louis, MO, USA) at 200 standard cubic centimeters per minute (sccm) and measured using a digital flow monitor (Alicat Scientific, Tucson, AZ, USA) before experimentation. Initially, the following apple cider vinegar (ACV) (Heinz, Pittsburgh, PA, USA) and benzaldehyde (BENZ) (CAS 100-52-7, SigmaAldrich) concentrations were tested: 5 ml of undiluted ACV (100%), ACV diluted in deionized H_2_O to make the final concentration of 1.5% (Lin et al., 2015) and BENZ diluted in mineral oil to make the final concentrations of 0.3% and 0.5%, respectively. For all further experiments, ACV was diluted in deionized H_2_O to make the final concentration of 1.5% (Lin et al., 2015), whereas BENZ was used at 0.5%. Odor solutions were freshly added to vials before the experiment, while the air was perfused through the Open-LA arenas.

### Optogenetic manipulation

For optogenetic self-stimulation experiments, an apparatus was developed that had six individual decuplet red LED (680 nm, Mouser Electronics, Mansfield, TX, USA) arrays, two universal 68 Ω resistances, a power source (12 V 30 A, S-360-12, Amazon) and a power driver shield kit (Sparkfun, Niwot, CO, USA) connected to an Arduino UNO 3.0 REV3 (Amazon) to operate the device. Activation parameters were set using a custom Python script (RRID:SCR_008394; available for download at https://github.com/kaunlab/OpenLA). Flies were exposed to 680 nm of red light at 30 Hz while in the ROSA. Air flow and odor perfusion were also controlled via a three-way solenoid valve, as described above.

Optogenetic stimulation parameters and all-*trans*-retinal (ATR) exposure necessary for the successful manipulation of circuit function were validated previously (Scaplen et al., 2019). For optogenetic manipulations, flies were raised on food containing 0.2 mmol l^−1^ ATR (Sigma-Aldrich, CAS 116-31-4) and maintained at this concentration post-eclosion until the completion of testing. Control flies for these experiments were raised on non-ATR food and were kept in the same rearing and testing conditions. To prevent ATR from light exposure and minimize nonspecific activation of Chrimson-expressing neurons, all vials, and holding containers were wrapped in aluminium foil and kept in dark conditions.

### Open-LA experimental procedures

We used sated flies to assess all behavior because starvation alters the response to appetitive and aversive stimuli, including: sensitizing olfactory receptor neurons (ORNs) wired to respond to attractive odors (Inagaki et al., 2014; Marella et al., 2012; Root et al., 2011), dampening the activity of ORNs wired for aversion (Inagaki et al., 2014; Ko et al., 2015), increasing sucrose sensitivity in sweet projection neurons (Kain and Dahanukar, 2015), decreasing sensitivity to bitter taste (LeDue et al., 2016), increasing locomotion (Lin et al., 2019; Yu et al., 2016), and reducing sleep (Keene et al., 2010).

Experiments were conducted starting at zeitgeber time 0–2 (when the lights in the incubator turned on). On the experimental day, individual flies were removed from group-housed containers by gentle aspiration and placed in each of the six chambers for their first 15 min self-administration session. The chambers were then placed within the Open-LA apparatus box, which was kept at 24°C with 70% humidity. Before tracking, flies were allowed to acclimate to the arena for 2 min. Depending on the experiment, flies could self-administer (1) apple cider vinegar (ACV) or benzaldehyde (BENZ) odor, (2) optogenetic activation of *Gr64f* (sweet) or *Gr66a* (bitter) gustatory receptor neurons (GRNs) via red light activation of CsChrimson, or (3) a combination of ACV and *Gr64f* GRNs activation or BENZ and *Gr66a* GRNs activation. Male or female flies were run, and in-between running the different sexes, the six chambers were washed with water and dried. Once flies finished their 15 min self-administration session, they were individually placed in food vials and maintained at 24°C with 70% humidity. The number of flies used for each experiment is indicated in the appropriate figure legends and Table S1.

### Preference index and operant learning index calculations

Time spent in ROSA/NoSA was used to obtain a preference index for each condition. The total time spent in each region (seconds) during the last seven minutes of tracking was used to calculate the preference index (PI) for the region of stimulus delivery. The PI was obtained by: [(total time spent flies in ROSA)–(total time spent in NoSA)]/(total time spent in ROSA+NoSA). A positive PI indicated a preference for ROSA and a negative index indicated a preference for NoSA.

Furthermore, operant learning indices were calculated for each behavioral metric and condition by comparing the behavioral metric at the beginning (1 min) and end (14 min) of the tracking session. The operant learning index was obtained by: [(total metric in ROSA at 14 min)–(total metric in ROSA at 1 min)]/(total metric in ROSA at 14 min+1 min). A statistically significant difference between the control and experimental group indicated operant learning occurred for that stimulus condition.

### T-maze forced odor assay

*w[1118]* (Bloomington *Drosophila* Stock Center #5905, RRID:BDSC_5905) flies were reared on Semi-Defined medium (See Bloomington *Drosophila* Stock Center recipe at https://bdsc.indiana.edu/information/recipes/germanfood.html), maintained at 25°C with 60–70% humidity, and kept on a 12 h:12 h light:dark cycle. Five-day-old mated female flies were starved for 24 h in a vial Kimwipe dampened with 1 ml of water. Then, 50 µl of 1:100 ACV (Heinz) diluted in water, or BENZ (CAS B1334, Sigma-Aldrich), diluted in mineral oil (CAS 8042-47-5, Sigma-Aldrich) were dispensed onto filter paper (CAS WHA10347512, Sigma-Aldrich) within each tube. The T-maze apparatus consisted of three chambers, two lower chambers for solvent and odor delivery (positions alternated across trials to minimize side preferences), and a detachable elevator that can be inserted into the core to move flies in and out (Fig. 1D). Flies (25–30) were gently transferred into the elevators, which were then inserted into the T-maze core and lowered until aligned with the bottom chambers. The T-maze apparatus was transferred into a 25°C incubator with 40–70% humidity and lights off. The flies acclimated for 60 s, after which the elevator doors opened, and the flies were given another 60 s to choose between the odor and control. Following the trial, flies were collected from each tube and those remaining in the elevator were transferred into the DNP tube. Fly death was recorded and subtracted from the final results. The tubes were frozen for 2–3 h for immobilization, and the number of flies was recorded. A conditioned preference index (CPI) was calculated by: [(no. flies in A)–(no. flies in B)]/(total no. flies in A+B). A positive CPI indicated a preference for an odor. For each trial, a participation index was also calculated by: [(no. flies in A)+(no. flies in B)]/(total no. flies in A+B+DNP). Trials with a participation index ≤0.5 (less than 50% of the flies participated) were excluded from the analysis.

### Statistical analysis

For the T-maze forced choice assay, one-way ANOVAs were run comparing preference indexes for ACV, BENZ and AIR followed by Bonferroni corrections using Prism version 10.0.0 (GraphPad Software, Boston, MA, USA, www.graphpad.com, RRID:SCR_002798). *Post hoc* analysis of .csv files from the Open-LA software or video files was conducted using custom-made MATLAB (MathWorks, Natick, MA) (RRID:SCR_001622; available to download at https://github.com/kaun-lab) and Python script (RRID:SCR_008394; https://github.com/kaun-lab).

Normal (Gaussian) distributions were tested using multiple tests: D'Agostino-Pearson omnibus, Shapiro–Wilk and Kolmogorov–Smirnov using Prism. One and two-way ANOVAs were performed on all data sets with planned contrasts on continuous datasets using Prism or custom-made Python script (RRID:SCR_008394; https://github.com/kaun-lab). Significant ANOVAs were followed by *post hoc* tests with Bonferroni or Dunn corrections. When examining the role of time in operant behaviors across a tracking session (line graphs), we used a mixed-effects model (REML) followed by *post hoc* tests with Bonferroni corrections. *t*-tests or nonparametric tests were used to compare discrete datasets (scatter plots) to compare behavior within and between operant outcomes (i.e. odor, taste, odor+taste).

For 15 min scatter plots, we used one-way ANOVA followed by Dunn multiple comparison test or Bonferroni tests. When comparing behavior across time bins of the operant session, we used a Wilcoxon matched-pairs signed rank test. QQ plots were used to examine the homogeneity of variance across odor types. If QQ plots suggested a non-normal distribution of data, nonparametric tests were reported, and degrees of freedom were corrected using Geisser-Greenhouse estimates of sphericity. When examining the effects of *Gr66a* or *Gr64f* activation on behavior, comparisons were made between groups exposed to ATR food and those in ATR-free food during development using the Mann–Whitney *U*-test. Detailed information about the statistical tests conducted for each experiment, along with the corresponding statistical results, can be found in Table S1.

Principal component analysis (PCA) was performed using Prism. Behavioral variables were standardized by converting values to z-score which were subsequently centered (Berman et al., 2014). Principal components were considered for analyses if they explained variance for more than one variable.

## RESULTS

### The Open-LA

Animals forage or navigate toward odor sources that could predict food (Chow and Frye, 2008; van Breugel and Dickinson, 2014; Wasserman et al., 2012). However, we lack a comprehensive understanding of how animals change their behaviors to pursue or avoid an odor or taste stimulus, and how taste can shape this. To study how animals respond to taste, and how the addition of taste in appetitive and aversive contexts shapes volitional behaviors over time, we created a novel automated Open-LA (Fig. 1A–C, Fig. S1A,B) that allows flies to self-administer odor and/or optogenetically activate neurons.

Within the Open-LA arena, the fly learns to self-administer odors and/or taste activation by entering the region of self-administration (ROSA). To terminate stimulus administration, flies enter the region of non-self-administration (NoSA) (Fig. 1C). We first analyzed total time spent administering a stimulus, total distance moved during administration, and maximum proximity to the delivery port (depth), which we termed session behaviors. Together, these determined overall pursuit or avoidance across a 15 min tracking session (Fig. 1D).

To capture subtle dynamics overlooked by session behaviors alone, we analyzed bouts, defined as single instances in which flies enter and subsequently exit from ROSA. These bout behaviors included stimulus administration frequency (total number of entries/bouts), mean bout duration, mean distance moved per bout and mean latency to re-enter ROSA after exiting (Fig. 1E). This analysis rigorously characterizes how reward and punishment shape behavioral choices, revealing the discrete behavioral choices resulting in pursuit and avoidance.

### Volitional behaviors to odors

**Fig. 2.**
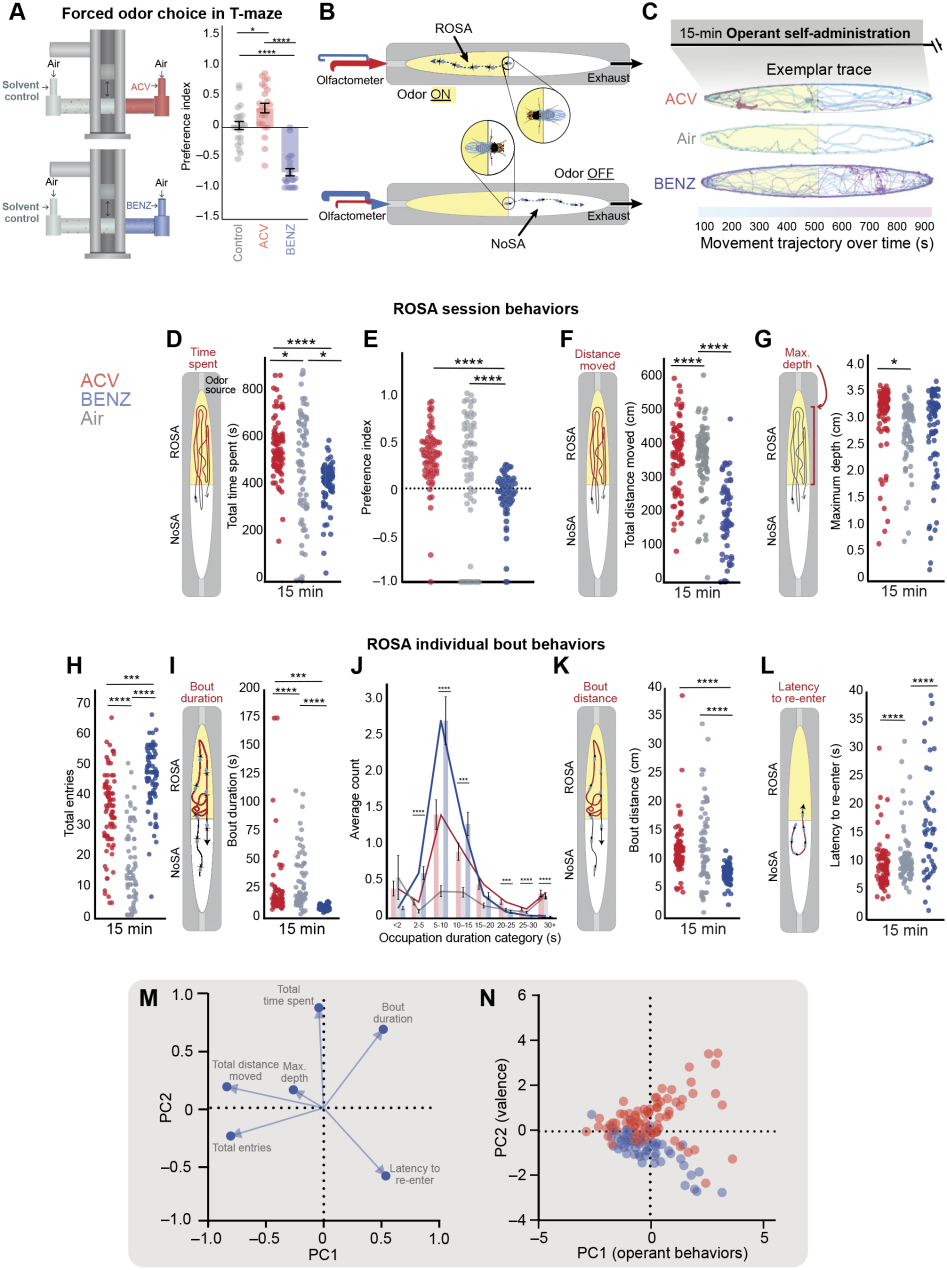
**Volitional behavioral repertoire observed upon odor exposure in the Open-LA assay.** (A) Forced choice T-maze assay apparatus demonstrates preference for apple cider vinegar (ACV; *n*=24) and avoidance of benzaldehyde (BENZ; *n*=23) and solvent control (*n*=22); data are presented as means±s.e.m. (B) In Open-LA, a fly learns to self-administer odors (ROSA) and terminate odor exposure (NoSA). (C) Exemplar trajectories depict movement trajectories over time for flies experiencing ACV, Air, and BENZ. *t*_0s_=blue, *t*_900s_=pink. (D–L) Canton-S flies were allowed to self-administer ACV (*n*=76), air (*n*=65) and BENZ (*n*=74). (D–G) Scatter plots depicting session behaviors in ROSA: (D) total time spent (sum across 15 min), (E) preference index, (F) total distance moved (sum across 15 min) and (G) maximum depth moved by each fly towards the odor port across 15 min. (H–L) Scatter plot depicting bout behaviors in ROSA: (H) total number of entries (count), (I) bout duration (seconds), (J) number of bouts within different occupation duration categories (seconds), (K) bout distance (centimeters) and (L) latency to re-enter (seconds). (M,N) Principal component analysis (PCA) was carried out using (M) behavioral metrics that describe (N) reward-type and valence differences in operant behavior upon odor exposure. Each data point represents one fly. Asterisks indicate significant differences, *P<0.05, ***P<0.001, ****P<0.0001. Detailed statistics in Table S1. Red dots indicate response to ACV; blue dots indicate response to BENZ.

Odor exposure influences subsequent odor response across time and often is studied in the context of seeking the source of appetitive odor streams (Álvarez-Salvado et al., 2018; Faucher et al., 2006; Semmelhack and Wang, 2009; Zhu et al., 2003). While previous studies have shed light on the behaviors flies employ to track odors (Budick and Dickinson, 2006; Kellogg et al., 1962) and the mechanisms underlying olfactory habituation (Sadanandappa et al., 2013), little is known about how flies make choices about odor access over time. We first demonstrated in a forced choice T-maze assay (Fig. 2A, Table S1) that flies prefer (*P*=0.0276) classically appetitive (ACV) and avoid (*P*<0.0001) aversive (BENZ) odors. To understand how flies freely pursue and avoid these odors, we used our Open-LA apparatus (Fig. 2B) to track flies self-administering ACV, BENZ or humidified air (Fig. 2C, Movies 1–3). We hypothesized that flies would choose to pursue the appetitive odors more than aversive odors. In order to investigate behavior independent of hunger state, and to control for the effects of food deprivation on locomotion, all of our experiments were performed in sated flies (Connolly, 1966).

We first assessed volitional session behaviors to investigate the operant learning outcome and found that flies chose to spend most of the session self-administering ACV odor and spent approximately half of the total time pursuing BENZ (Fig. 2D, *P*<0.0001; Table S1). Flies also self-administered air but spent less time administering air than ACV (Fig. 2D, *P*=0.0100; Table S1) and more time with air than BENZ (Fig. 2D, *P*=0.0100; Table S1). When we calculated a preference index using total time spent in ROSA relative to NoSA, it was clear that flies show a preference for ACV and air but not BENZ (Fig. 2E, *P*<0.0001; Table S1). While in the ROSA, flies moved more in the presence of ACV than air (Fig. 2F, *P*<0.0001; Table S1) or BENZ (Fig. 2F, *P*<0.0001; Table S1), and stayed closer to the odor port for ACV than to that for air (Fig. 2G, *P*=0.016; Table S1), but not for BENZ (Fig. 2G, *P*>0.05; Table S1). Together, the increase in time spent, movement and depth towards odor port reflected an increase in volitional pursuit of ACV. Conversely, although BENZ did not elicit a negative preference index (Fig. 2E), the observed decrease in time spent and movement relative to air reflected avoidance of BENZ.

Next, we assessed volitional bout behaviors to investigate the operant learning process. Flies had less frequent, but longer bouts for air than ACV or BENZ (Fig. 2H–J, *P*<0.0001; Table S1). They also had less frequent, but longer bouts with ACV and more frequent but shorter bouts administering BENZ (Fig. 2H–J, *P*<0.001; Table S1). Flies also moved more per bout in the presence of ACV than BENZ (Fig. 2K, *P*<0.001; Table S1). Compared with latency for air, flies showed a shorter latency to re-administer ACV and a longer latency for BENZ (Fig. 2L, *P*<0.0001; Table S1). Collectively, these data suggest that flies will choose to experience an aversive odor, but also choose to spend less time, move less and avoid the BENZ odor source, indicating a nuanced strategy for stimulus pursuit and avoidance.

To understand how these behaviors could predict odor valence, we implemented the linear dimensionality reduction technique PCA (Odell et al., 2023; Godesberg, Bockemühl and Büschges, 2024). This allowed us to graphically represent the distribution of the behavioral metrics tested (Fig. 2M) and each fly's experience (i.e. ACV or BENZ self-administration; Fig. 2N). Operant outcomes and bouts for odor could be distinguished by the second principal component (PC2; Fig. 2M,N), which separated appetitive from aversive odor choices by distinct behaviors. Motivation for ACV could be explained by total distance traveled in ROSA, proximity to ROSA odor port (max. depth), total time spent in ROSA and bout duration in ROSA, whereas BENZ behavior was best predicted by total entries into ROSA and latency to re-enter behaviors (Fig. 2M,N). Overall, these results indicate that flies make distinct choices depending on the odor valence, engaging in behaviors that culminate in pursuit of an appetitive odor and avoidance of an aversive one.

### Operant response to odors

**Fig. 3.**
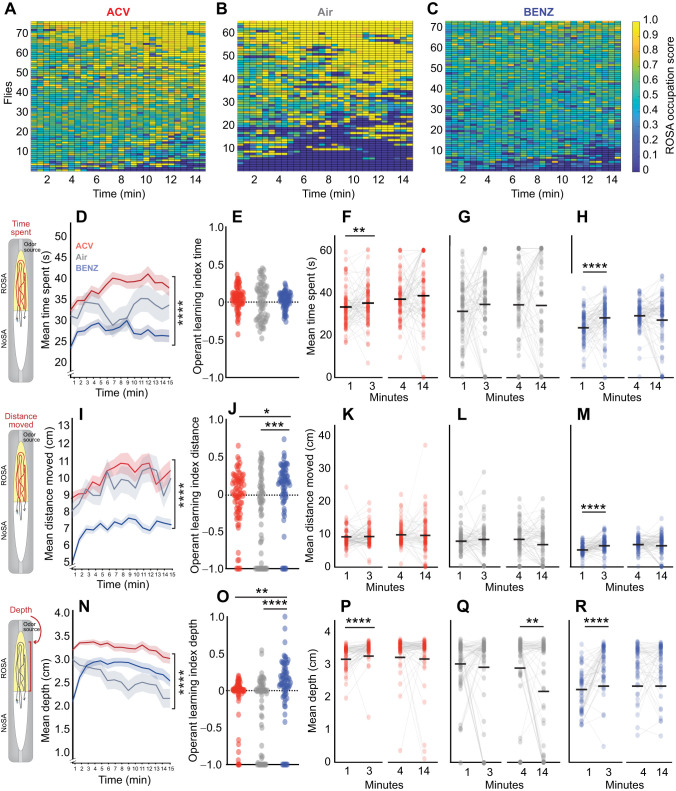
**Behavior dynamics over time uncover early operant learning upon odor exposure.** (A–C) Heat maps for each fly experiencing (A) apple cider vinegar (ACV, *n*=76), (B) air (*n*=65) and (C) benzaldehyde (BENZ, *n*=74). Scores indicate ROSA occupancy; yellow=high, blue=low. (D–H) Mean time spent (seconds) in ROSA. (I–M) Mean distance moved (centimeters) in ROSA. (N-R) Mean depth towards the ROSA odor port across 15 min. (E,J,O) Operant learning index for each metric. (F–H, K–M, P–R) Comparison of each metric over time: 0–1 min (bin 1) versus 2–3 min (bin 3) and 3–4 min (bin 4) versus 13–14 min (bin 14) for ACV, air and BENZ. For line graphs, data are mean±s.e.m., and for scatter plots, each data point represents one fly. Asterisks indicate significant differences, **P<0.01, ****P<0.0001. Detailed statistics in Table S1.

To study whether operant learning occurred in response to individual odors, we obtained Boolean ROSA occupation scores for air and odors throughout the 15 min tracking session (Fig. 3A–C) and analyzed how volitional behavior changed over time (Fig. 3D–R). To evaluate if operant learning occurred, we first calculated an operant learning index by comparing the behavioral metric in ROSA at the 14 min to the 1 min marks. The ROSA score at 1 min was subtracted from the ROSA score at 14 min, and that value was then divided by the sum of the two scores.

Throughout the session, time spent was greater for ACV than BENZ (Fig. 3D; *P*<0.0001; Table S1). Although no operant learning index differences were observed for time spent throughout the session (Fig. 3E), comparing behavioral metrics between the early and late time bins revealed that time spent increased within the first 3 min for both ACV (Fig. 3F, *P*=0.0083; Table S1) and BENZ (Fig. 3H, *P*<0.0001; Table S1). Distance traveled was also greater for ACV than BENZ (Fig. 3I, *P*<0.0001; Table S1). The operant learning index revealed that, although flies pursued ACV to a greater extent than BENZ, operant learning was greatest for BENZ relative to other stimuli for distance traveled (Fig. 3J, Air *P*=0.0004, ACV *P*= 0.0452; Table S1), an effect that could be attributed to operant learning early in the session (Fig. 3M, *P*<0.0001). Closeness to the odor port (depth) was also greater for ACV compared with BENZ (Fig. 3N; *P*<0.0001; Table S1); however, operant learning occurred to a greater extent for BENZ relative to ACV and air (Fig. 3O, Air *P*<0.0001, ACV *P*=0.0014; Table S1). Depth in ROSA increased within the first 3 min for both ACV (3P, *P*<0.0001; Table S1) and BENZ (3R, *P*<0.0001; Table S1). When air was administered, we noticed that flies significantly reduced their proximity to the port across the session (Fig. 3Q, *P*=0.0053; Table S1). These data demonstrate that multiple behavioral parameters change over time, with a more prevalent change early in the session, suggesting that operant learning occurs rapidly in response to aversive and appetitive odors.

We next asked whether flies behave differently when avoiding odors or humidified air while in NoSA. After self-administering ACV or BENZ, flies spent more time and moved more in NoSA for BENZ than for ACV (Fig. S1C-F, *P*<0.0001; Table S1). They also traveled closer to the BENZ vacuum port compared with the ACV vacuum port (Fig. S1G; S1H, *P*=0.0051; Table S1), which suggests a strategy to actively avoid the BENZ odor. In air experiments, we observed that flies reduce their proximity to air vacuum ports (Fig. S1G, *P*<0.0001; Table S1), which reflects a generalized reduction in stimulus pursuit (Fig.S1H, Table S1). Flies also exhibited longer bouts when avoiding BENZ in NoSA relative to ACV (Fig. S1I, *P*<0.0001; Table S1) but displayed similar bout distances in NoSA when avoiding ACV and BENZ (Fig. S1J, *P*=0.0792; Table S1). Thus, flies utilize multiple behaviors to actively avoid self-administration of BENZ more than they did for ACV.

One concern is that odor concentration influences operant behavior. To confirm that behaviors were not influenced by receptor desensitization or resensitization rates (Lin et al., 2015), we tested additional doses of ACV (Lin et al., 2015) and BENZ. Our results indicate this mechanism is not at play, since our behavioral metrics are consistent between different appetitive (Fig. S1K–N, *P*>0.05; Table S1) or aversive (Fig. S1O–R, *P*>0.05; Table S1) odor concentrations.

When we examined sex differences in volitional behaviors for odors and air, we observed subtle differences between male and female flies in ACV (Fig. S2A–K) and BENZ (Fig. S2L–V). Generally, we observed that females moved greater distances (Fig. S2C, *P*=0.0075; S2N, *P*=0.0271; Table S1) and pursued the ROSA odor ports more than males for both odors (Fig. S2E, *P*=0.0384; S2P, *P*=0.0208; Table S1). On the other hand, most bout behaviors between females and males were comparable between odors (Fig. S2G–K, Fig. S2R–V, Table S1). These data suggest that females could be more motivated to explore in response to appetitive and aversive odors than males are, highlighting the importance of examining sex differences in odor choice.

### Volitional behaviors to taste

**Fig. 4.**
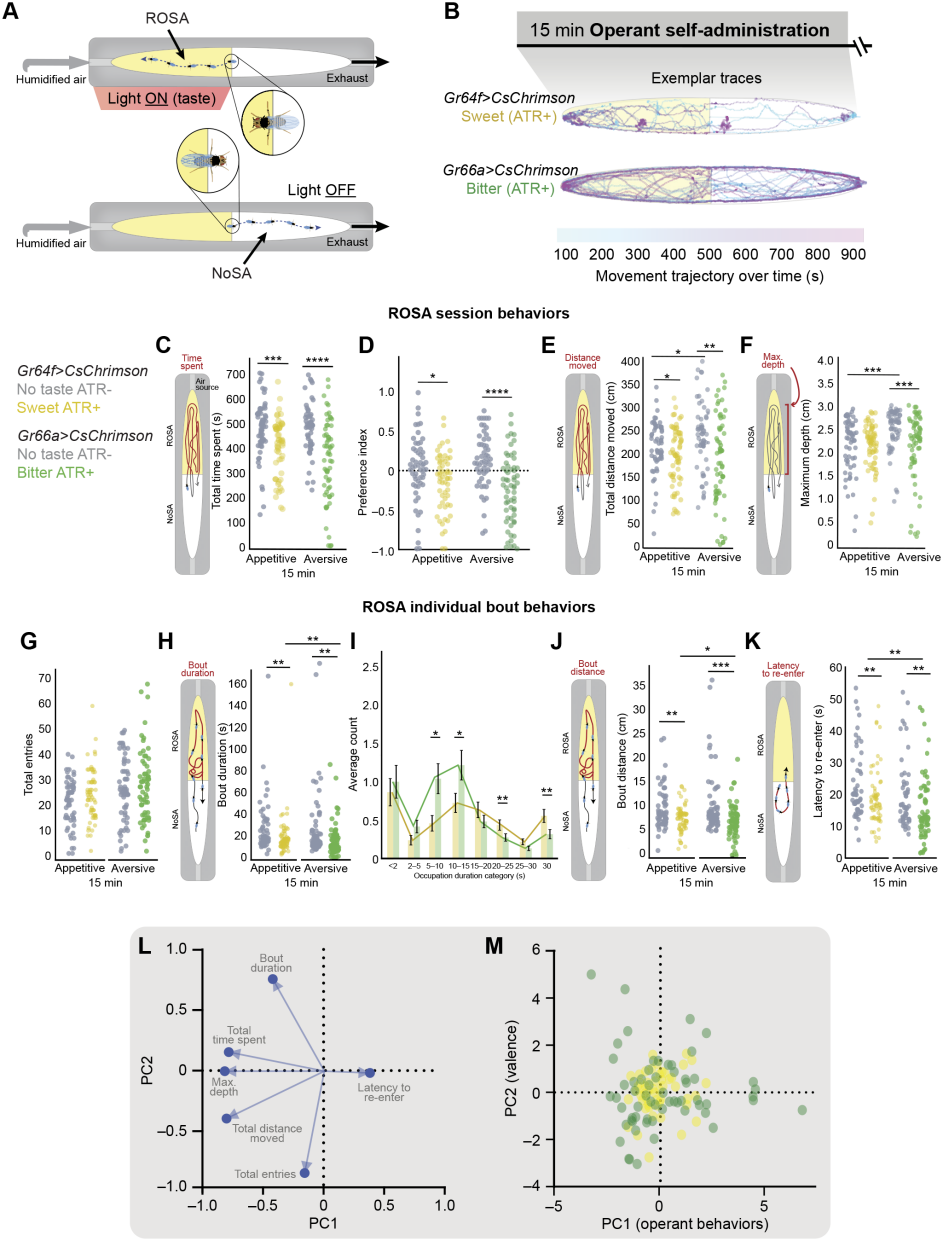
**Volitional behavioral repertoire observed upon taste activation in the Open-LA assay.** (A) Flies activate taste neurons expressing *CsChrimson* via red light (ROSA) and to terminate taste activation (NoSA). (B) Exemplar trajectories depict movement trajectory over time for ATR+ flies experiencing taste. *t*_0s_=blue, *t*_900s_=pink. (C–J) Metrics of behaviors are shown for *Gr64f>CsChrimson* no taste controls (ATR–) (*n*=56), *Gr64f>CsChrimson* sweet taste (ATR+) (*n*=58), *Gr66a>CsChrimson* no taste controls (ATR–) (*n*=57), and *Gr66a>CsChrimson* bitter taste (ATR+) (*n*=61) flies. (C–F) Scatter plots depicting session behaviors in ROSA: (C) total time spent (sum across 15 min), (D) preference index, (E) total distance moved (sum across 15 min), and (F) maximum depth moved by each fly towards odor port across 15 min. (G–K) Scatter plots depicting bout behaviors in ROSA: (G) total number of entries (count), (H) bout duration (seconds), (I) number of bouts within different occupation duration categories (seconds), (J) bout distance (centimeters), (K) latency to re-enter (seconds). (L,M) Principal component analysis (PCA) was carried out using (L) behavioral metrics that describe (M) reward-type and valence differences in operant behavior upon taste activation. Each data point represents one fly. Asterisks indicate significant differences, *P<0.05, **P<0.01, ***P<0.001, ****P<0.0001. Detailed statistics in Table S1. Yellow dots indicate response to sweet GRN activation; green dots indicate response to bitter GRN activation.

To measure how fly behavior is shaped by repeated self-administration of taste alone, we modified the Open-LA apparatus so flies received optogenetic activation of gustatory receptor neurons, without odor, when they entered ROSA (Fig. 4A). We tracked flies as they self-administered sweet taste via activation of *CsChrimson* in *Gr64f*-expressing neurons (Jiao et al., 2008; Kwon et al., 2014; Marella et al., 2006; Musso et al., 2019; Thorne et al., 2004; Wang et al., 2004) or bitter taste via activation of *Gr66a*-expressing neurons (Fig. 4B, Movies 4,5) (Dahanukar et al., 2001; Jiao et al., 2008; Kwon et al., 2014; Marella et al., 2006; Musso et al., 2019; Thorne et al., 2004; Wang et al., 2004). Before performing operant experiments, we confirmed that activation of *Gr64f*- and *Gr66a*-expressing neurons in sated flies was able to induce or inhibit proboscis extension, respectively. ATR is a light-sensitive chromophore that functions as a co-factor for opsins, facilitating the light-controlled activation of ion channels. We compared behavior in ATR– controls to ATR+ flies to be sure that the effects we observed were due to gustatory receptor neuron activation as opposed to red light. We hypothesized that flies would spend more time with the ROSA region in response to sweet neuron activation compared with activation of bitter neurons (Fu et al., 2023; Sareen et al., 2021).

We first assessed session behaviors and found that, unexpectedly, optogenetic activation of sweet and bitter receptor neurons led to a similar significant reduction in the time spent (Fig. 4C, appetitive *P*=0.0003, aversive *P*<0.0001; Table S1) and distance traveled (Fig. 4E, appetitive *P*=0.0200, aversive *P*=0.0019; Table S1) in ROSA. This effect was also reflected as a significant reduction in preference for taste experiences (Fig. 4D, appetitive *P*=0.02, aversive *P*<0.0001; Table S1). Interestingly, flies stayed closer to the air port when experiencing bitter taste (Fig. 4F, *P*=0.0006; Table S1), but not sweet taste (Fig. 4F, *P*>0.05; Table S1).

When we analyzed bout behaviors, we found that bout frequency was comparable across conditions (Fig. 4G, appetitive and aversive *P*>0.05; Table S1), and taste activation resulted in shorter bout durations (Fig. 4H, appetitive *P*=0.0055, aversive *P*=0.0012; Table S1), bout distances (Fig. 4J, appetitive, *P*=0.0083, aversive *P*=0.0001; Table S1) and latencies to re-administer (Fig. 4K, appetitive *P*=0.0055; aversive *P*=0.0012; Table S1), regardless of the stimuli valence. Comparison between the valences revealed that flies demonstrated a strategic behavioral shift in bout behavior for sweet versus bitter taste activation. Compared with sweet taste activation, bitter taste activation caused greater decreases in bout duration (Fig. 4H, *P*=0.0058; Table S1), which could be attributed to a reduction in longer duration bouts (Fig. 4I; 20–25 s bouts, *P*=0.0123; 30 s bouts, *P*=0.0027; Table S1) and a shift towards shorter bout durations (5–15 s). Moreover, bitter taste decreased bout distances (Fig. 4J, *P*=0.0110; Table S1) and reduced the latency between bouts (Fig. 4K, *P*=0.0058; Table S1) compared with sweet taste activation.

We then asked whether the behaviors observed could predict taste valence. In contrast to odor-only experiments, a PCA for behaviors exhibited in sweet and bitter taste experiments (Fig. 4L) demonstrated that flies used a similar behavioral repertoire for both taste experiences (Fig. 4M). No distinction across the second principal component was observed, indicating that behaviors from each taste valence overlapped (PC2; Fig. 4M).

Together, these data demonstrate that taste activation reduces the volitional behaviors used to pursue gustatory stimuli, irrespective of the taste's valence. Furthermore, while overall session outcomes were not influenced by taste valence (no net increase in sweet pursuit or bitter avoidance), significant shifts in bout behaviors suggest that flies employ distinct strategies to avoid bitter stimulation. This indicates that although flies are capable of self-administering taste activation, accounting for broad behavioral choices does not differentiate appetitive and aversive taste responses, unlike our original prediction. This underscores the importance of analyzing not only the culminating outcome of fly choices over time, but also the individual behaviors that co-vary within a session to lead to that behavioral outcome.

### Operant response to taste

**Fig. 5.**
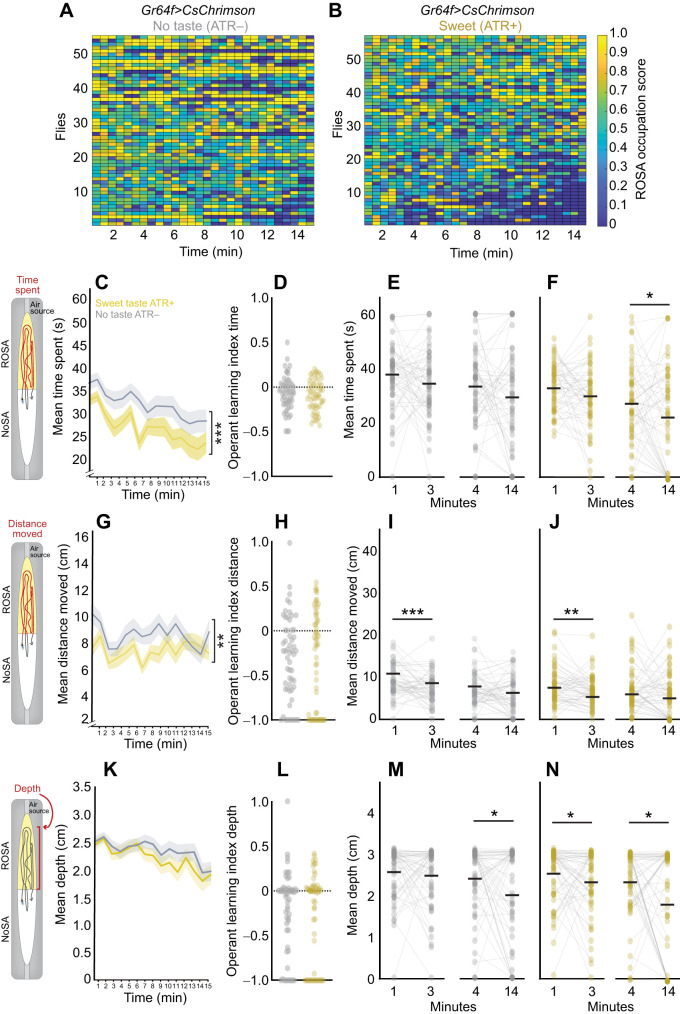
**Behavior dynamics over time uncover slight operant learning upon sweet taste activation.** (A,B) Heat maps for each *Gr64f>CsChrimson* fly experiencing (A) no taste (ATR–) (*n*=56) and (B) sweet Taste (ATR+) (*n*=58). Scores indicate ROSA occupancy; high=yellow, low=blue. (C–F) Mean time spent (seconds) in ROSA. (G–J) Mean distance moved (centimeters) in ROSA. (K–N) Mean depth towards the ROSA odor port across 15 min. (D,H,L) Operant learning index for each metric. (E,F,I,J,M,N) Comparison of each metric over time: 0–1 min (bin 1) versus 2–3 min (bin 3) and 3–4 min (bin 4) versus 13–14 min (bin 14) for no taste (ATR–) and sweet taste (ATR+). For line graphs, data are means±s.e.m. and for scatter plots, each data point represents one fly. Asterisks indicate significant differences, *P<0.05, **P<0.01, ***P<0.001. Detailed statistics in Table S1.

**Fig. 6.**
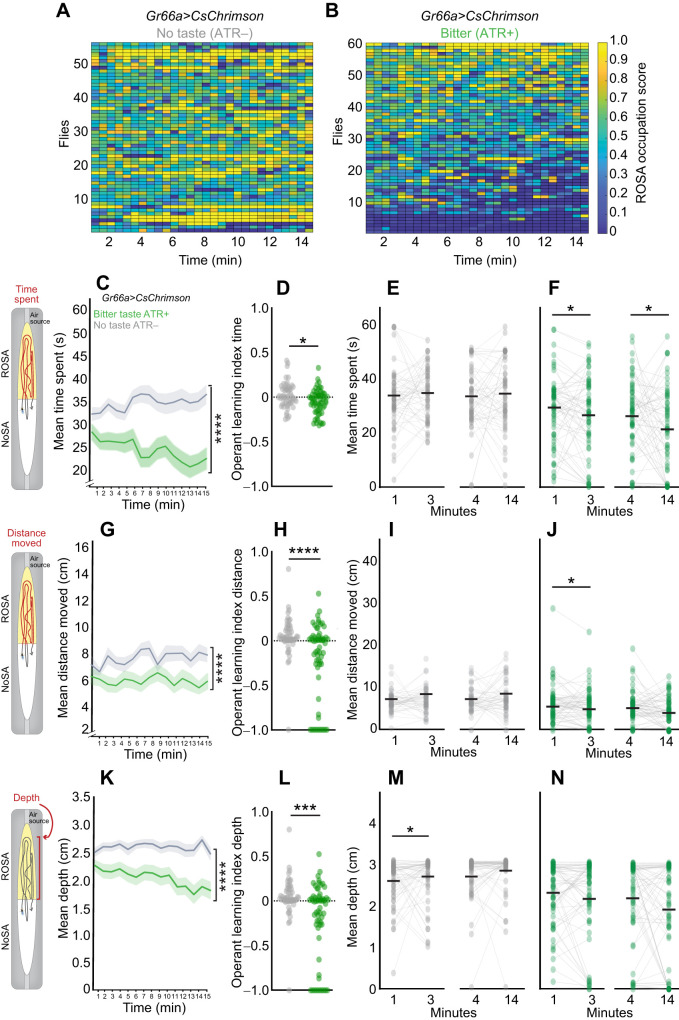
**Behavior dynamics over time uncover slight operant learning upon bitter taste activation.** (A,B) Heat maps for each *Gr66a>CsChrimson* fly experiencing (A) No taste (ATR−) (*n*=57) and (B) Bitter Taste (ATR+) (*n*=61). Scores indicate ROSA occupancy; high=yellow, low=blue. (C–F) Mean time spent (seconds) in ROSA. (G–J) Mean distance moved (centimeters) in ROSA. (K–N) Mean depth towards the ROSA odor port across 15 min. (D,H,L) Operant learning index for each metric. (E,F,I,J,M,N) Comparison of each metric over time: 0–1 min (bin 1) versus 2–3 min (bin 3) and 3–4 min (bin 4) versus 13–14 min (bin 14) for no taste (ATR–) and bitter taste (ATR+). For line graphs, data are means±s.e.m. and for scatter plots, each data point represents one fly. Asterisks indicate significant differences, *P<0.05, ****P<0.0001. Detailed statistics in Table S1.

To study whether operant learning occurred in response to appetitive (Fig. 5) and aversive (Fig. 6) taste activation, we tracked sated flies during activation of sweet (*Gr64f>CsChrimson*; Fig. 5A,B) and bitter (*Gr66a>CsChrimson*; Fig. 6A,B) taste receptor neurons and analyzed how volitional behaviors changed over time (Figs 5C–N and 6C–N). In these experiments, operant learning was defined by the presence of behavioral changes in flies experiencing taste (ATR+) and the absence of such changes in control flies without taste experience (ATR–).

We observed that flies experiencing either taste decreased their time spent (Fig. 5C, *P*=0.0006; 6C, *P*<0.0001; Table S1) and distance traveled (Fig. 5G, *P*=0.0032; 6G, *P*<0.0001; Table S1) in ROSA across the session, and only bitter taste elicited a decrease in air port engagement (Fig. 6K, *P*<0.0001; Table S1). The operant learning index calculated by comparing the metrics at the beginning and the end of the session revealed that only bitter taste significantly increased operant learning (Fig. 5D,H,L, *P*>0.05; Fig. 6D, *P*=0.0123; Fig. 6H, *P*<0.0001; Fig. 6L *P*=0.0008; Table S2). We also show that modest operant learning in response to sweet occurred within discrete time points for some behaviors. Decreases in time spent emerged later for sweet (Fig. 5E,F; *P*<0.01; Table S1) and occurred progressively across the session for bitter (Fig. 6E,F, *P*<0.01; Table S1) taste. Other behavioral metrics showed subtle differences in operant learning between sweet and bitter taste activation: bitter taste decreased the distance traveled early in the session (Fig. 6I,J, *P*<0.01; Table S1), whereas sweet taste decreased air port proximity (depth) early in the session (Fig. 5M,N, *P*<0.01; Table S1). In summary, these behavioral changes indicate operant learning occurred at different time points based on the valence of the taste experienced.

We next asked if flies behave differently when avoiding taste activation while in the NoSA. In the context of session behaviors, flies spend more time (Fig. S3A, *P*=0.0006; Fig. S3B,C, *P*<0.0001; Table S1) and move more (Fig. S3D, *P*=0.0305; Fig. S3E, *P*=0.0005; Fig. S3F, *P*=0.0078, *P*=0.0340; Table S1) in the NoSA region when avoiding gustatory receptor neurons independent of taste valence, displaying no changes in depth traveled towards vacuum ports (Fig. S3G–I, *P*>0.05; Table S1). In the context of bout behaviors, flies increased bout duration (*P*=0.0123) and bout distance (*P*=0.0074) when avoiding bitter taste (Fig. S3J,K, Table S1). Together, these results indicate that although both sweet and bitter taste induce avoidance behaviors, bitter taste induces a stronger avoidance than sweet taste.

Subtle sex differences were observed when comparing volitional behaviors for sweet (Fig. S4A–J) and bitter (Fig. S4K–T) gustatory neuron activation. Similarly to odor-only experiments, we observed that females spent more time (Fig. S4A, *P*=0.0026; Fig. S4K, *P*<0.0001; Table S1) while activating taste receptor neurons regardless of valence compared with males. When assessing bout behaviors, males displayed slightly increased avoidance bout behaviors in NoSA upon sweet and bitter neuron activation compared with females (Fig. S4G–J and Q–T; *P*≤0.0465; Table S1). Together, these data demonstrate that females may be more motivated to pursue taste activation than males, highlighting interesting sex differences in response to gustatory stimuli.

### Volitional behaviors to combined odor and taste

**Fig. 7.**
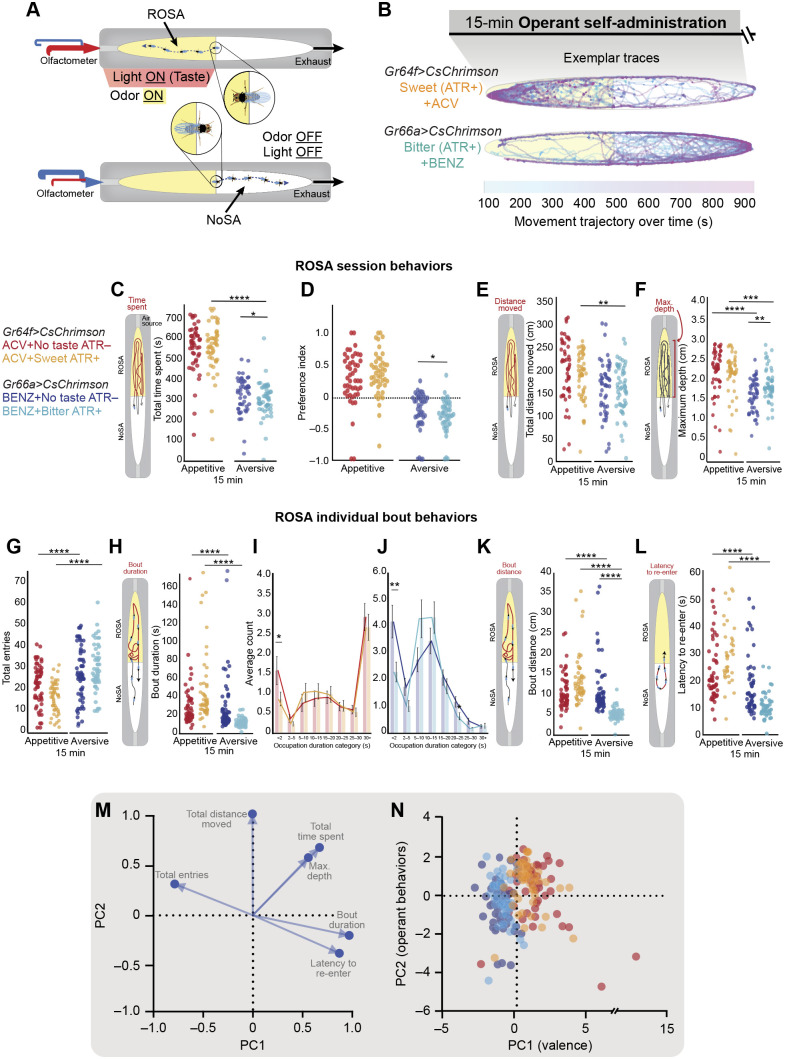
**Volitional behavioral repertoire observed upon concurrent odor and taste experience in the Open-LA assay.** (A) Flies learn to simultaneously self-administer odors and activate taste neurons expressing *CsChrimson* via red light (ROSA) and to terminate stimulus exposure (NoSA). (B) Exemplar trajectories depict movement trajectory over time for ATR+ flies experiencing sweet taste+ACV and bitter taste+BENZ, *t*_0s_=blue, *t*_900s_=pink. (C–K) Behaviors are shown for *Gr64f>CsChrimson* flies experiencing ACV without taste (ATR–) (*n*=47) and ACV with sweet taste (ATR+) (*n*=47), as well as *Gr66a>CsChrimson* flies experiencing BENZ without taste (ATR−) (*n*=49) and BENZ with bitter taste (ATR+) (*n*=48). (C–F) Scatter plots depicting session behaviors in ROSA: (C) total time spent (sum across 15 min), (D) preference index, (E) total distance moved (sum across 15 min) and (F) maximum depth moved by each fly towards the odor port across 15 min. (G–L) Scatter plot depicting bout behaviors in ROSA: (G) total number of entries (count); (H) bout duration (seconds); number of bouts within different occupation duration categories (seconds) for (I) appetitive and (J) aversive stimuli; (K) bout distance (centimeters); and (L) latency to re-enter (seconds). (M,N) Principal component analysis (PCA) was carried out using (M) subtle behavioral metrics that describe (N) reward-type and valence differences in operant behavior upon exposure to unisensory/multisensory stimuli. Each data point represents one fly. Asterisks indicate significant differences, *P<0.05, **P<0.01, ***P<0.001, ****P<0.0001. Detailed statistics in Table S1.

Understanding how animals pursue odors while simultaneously experiencing taste is crucial for revealing how they weigh and integrate multisensory cues to make decisions. To characterize how taste experience shapes odor self-administration, we tracked the behavior of sated flies administering both ACV and sweet taste or BENZ and bitter taste (Fig. 7A,B, Movies 6,7). We compared ATR+ flies that experienced both taste and odor with ATR– flies that experienced odor alone. We hypothesized that taste neuron activation would enhance an animal's response to an odor and alter the operant trajectory compared with odor alone.

When examining volitional session behaviors, we observed that flies chose to pursue multisensory appetitive stimuli more than the multisensory aversive stimuli (Fig. 7C–F, *P*<0.001; Table S1). However, contrary to our expectation, sweet taste did not enhance ACV pursuit since the time spent, distance traveled and nearness to the odor port (depth) in ROSA remained unchanged (Fig. 7C–F, *P*>0.05; Table S1). Notably, flies experiencing bitter taste and BENZ spent less time (Fig. 7C, *P*=0.0400; Table S1) and moved less (Fig. 7E, *P*>0.05; Table S1) in ROSA compared with BENZ alone, an effect that was also reflected in a decrease in preference (Fig. 7D, *P*=0.02; Table S1). Unexpectedly, we observed that flies experiencing bitter taste with BENZ moved closer to odor ports than flies experiencing BENZ only (Fig. 7F, *P*=0.0030; Table S1). Together, these data suggest that aversive taste activation was sufficient to induce modest changes in odor-induced volitional behaviors.

We next wanted to determine if bout behaviors reflected unique effects of taste on odor response. Multisensory experience did not influence the frequency of bouts (Fig. 7G) or mean bout duration for ACV or BENZ (Fig. 7H). Interestingly, appetitive stimuli (Fig. 7I) promoted more longer-duration bouts into ROSA compared to aversive stimuli (Fig. 7J), regardless of whether stimulus presentation was odor-only or odor combined with taste. Regardless of valence, concurrent taste activation only decreased the number of short duration bouts compared with odor-only controls (Fig. 7I,J; Table S1), causing a shift that increased mid-range bout durations. The effects elicited by bitter taste were slightly stronger than those elicited by sweet taste (Fig. 7I,J). Only bitter taste reduced bout distance in response to odors (Fig. 7K; *P*<0.0001; Table S1). Regardless of odor valence, experiencing taste did not affect the latency to re-administer odors (Fig. 7L). Together, these results indicate that experience of taste in sated flies does not influence the pursuit or avoidance we observed for odor stimuli, independent of valence. Moreover, only aversive odor and bitter taste triggers subtle behavioral adjustments (specifically, a shift towards shorter bout durations and reduced bout distances), implying a nuanced avoidance mechanism.

A PCA analysis revealed that operant pursuit and bout behaviors for different valences were distinguished by the first principal component (PC1; Fig. 7M,N), which separated behaviors for appetitive and aversive stimuli. However, the second principal component (PC2; Fig. 7M,N) did not distinguish between the operant behaviors for odor-only and multisensory modality. Together, these results suggest that behavioral changes in response to stimuli of different valence appear to be dictated mainly by odor.

### Operant response to combined odor and taste

**Fig. 8.**
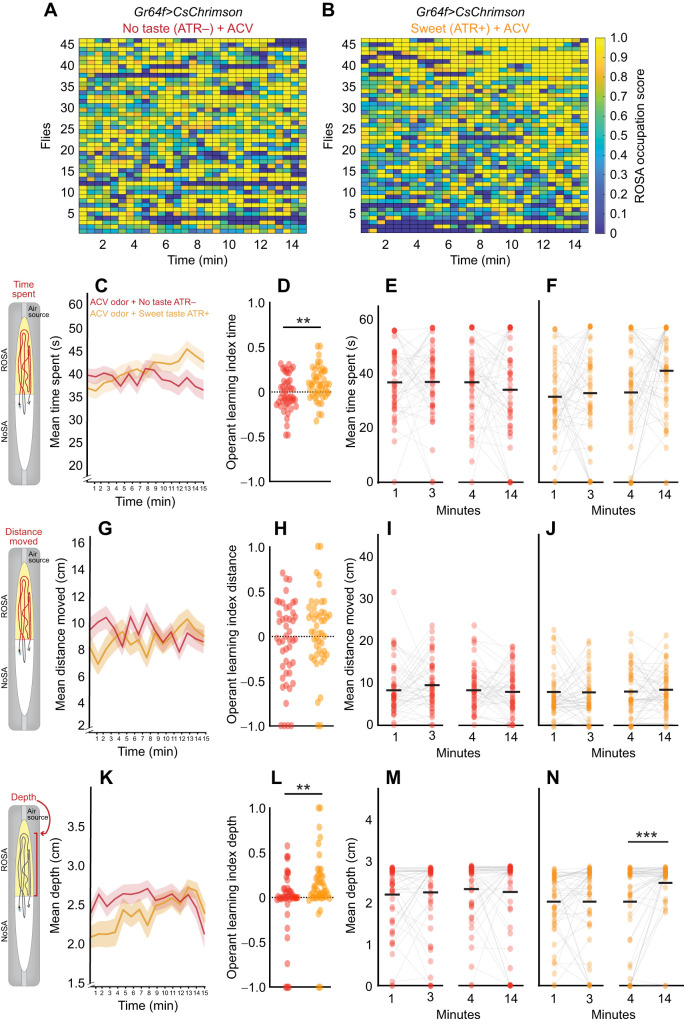
**Behavior dynamics over time indicate sweet taste generally does not potentiate operant learning for odors.** (A,B) Heat maps for each *Gr64f>CsChrimson* fly experiencing (A) ACV without taste (ATR−) (*n*=47) and (B) ACV with sweet taste (ATR+) (*n*=47). Scores indicate ROSA occupancy; high=yellow, low=blue. (C–F) Mean time spent in ROSA (seconds). (G–J) Mean distance moved in ROSA (centimeters). (K–N) Mean depth towards the ROSA odor port across 15 min. (D,H,L) Operant learning index for each metric. (E,F,I,J,M,N) Comparison of each metric over time: 0–1 min (bin 1) versus 2–3 min (bin 3) and 3–4 min (bin 4) versus 13–14 min (bin 14) for ACV and no taste (ATR–; red) and ACV with sweet taste (ATR+; orange). For line graphs, data are mean±s.e.m., and for scatter plots, each data point represents one fly. Asterisks indicate significant differences, ***P<0.001. Detailed statistics in Table S1.

**Fig. 9.**
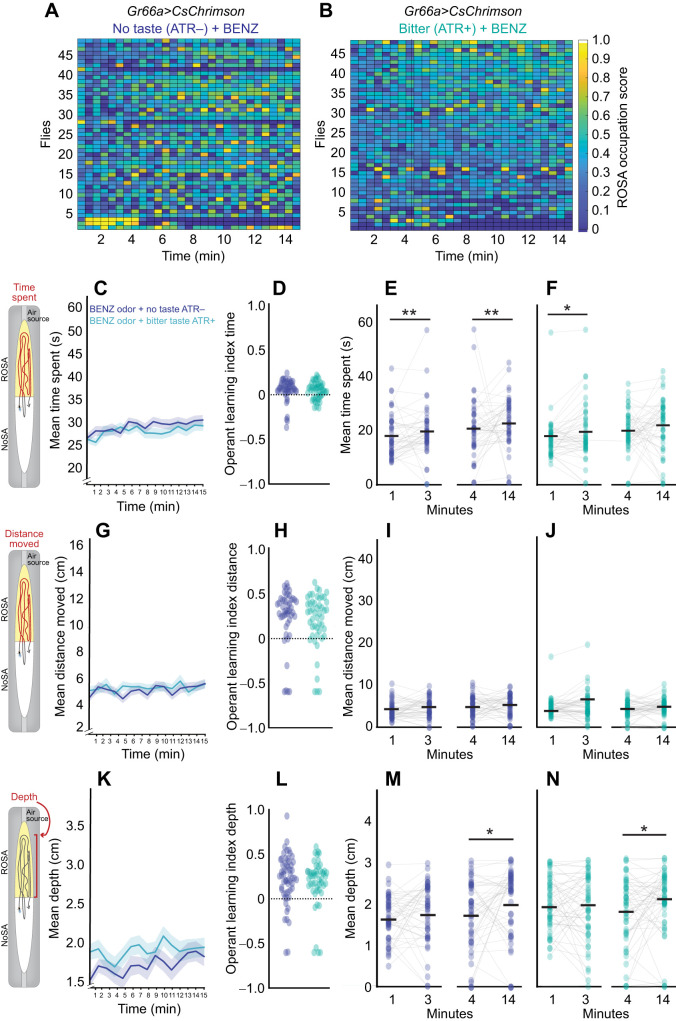
**Behavior dynamics over time indicate bitter taste generally does not potentiate operant learning for odors.** (A,B) Heat maps for each *Gr66a>CsChrimson* fly experiencing (A) BENZ without taste (ATR−) (*n*=49) and (B) BENZ with bitter taste (ATR+) (*n*=48). Scores indicate ROSA occupancy; high=yellow, lower=blue). (C–F) Mean time spent in ROSA (seconds). (G–J) Mean distance moved in ROSA (centimeters). (K–N) Mean depth towards the ROSA odor port across 15 min. (D,H,L) Operant learning index for each metric. (E,F,I,J,M,N) Comparison of each metric over time: 0–1 min (bin 1) versus 2–3 min (bin 3) and 3–4 min (bin 4) versus 13–14 min (bin 14) for BENZ without taste (ATR−; dark blue) and BENZ with Bitter taste (ATR+; turquoise). For line graphs, data are means±s.e.m., and for scatter plots, each data point represents one fly. Asterisks indicate significant differences, *P<0.05, **P<0.01. Detailed statistics in Table S1.

To study whether operant learning in response to odors was influenced by appetitive (Fig. 8) and aversive taste activation (Fig. 9), we obtained individual Boolean ROSA occupation scores to compare flies experiencing odors (ATR–) (Figs 8A and 9A) with those experiencing concurrent odor and taste (ATR+) (Figs 8B and 9B) across the tracking session. We then analyzed how volitional behaviors changed over time in response to appetitive (Fig. 8C–N) and aversive stimuli (Fig. 9C–N).

Modest operant learning was evident for time spent in the ROSA upon concurrent activation of sweet taste and exposure to ACV, since a greater operant learning index was observed (Fig. 8D; *P*=0.004; Table S1) relative to odor-only controls. However, when comparing time spent in ROSA in the first to third or fourth to fourteenth minute, there were no significant differences (Fig. 8E,F, *P*>0.05; Table S1). The operant learning index was also increased for the depth metric (Fig. 8L; *P*=0.006; Table S1) relative to odor-only controls, suggesting that operant learning occurred. There was a significant increase in depth in the fourteenth compared to the fourth minute in response to sweet plus ACV, suggesting that sweet taste stimulus increases proximity to the odor port (Fig. 8M,N, *P*<0.001; Table S1).

In contrast, operant learning was not observed upon concurrent activation of bitter taste and exposure to BENZ (Fig. 9). Activation of aversive taste did not affect aversive odor responses over time, since time spent, distance moved and proximity to odor ports in ROSA remained unchanged relative to odor-only controls (Fig. 9C–N; Table S1). Overall, appetitive multisensory exposure had a modest effect on operant learning, and aversive multisensory exposure had no effect. Thus, these results indicate that appetitive taste has a limited influence on volitional olfactory response.

We next asked whether flies behave differently when avoiding taste activation while in NoSA. In the context of session behaviors, flies that experienced aversive stimuli spent more time (*P*<0.0001) and moved greater distances (*P*<0.0001) in NoSA compared with flies that experienced appetitive stimuli, and the addition of taste activation did not influence these behaviors (Fig. S5A–F, Table S1). In the context of bout behaviors, bout duration in NoSA was comparable between appetitive and aversive stimuli (Fig. S5J, *P*>0.05; Table S1), and there was only a slight increase in bout distance within NoSA for the appetitive stimuli compared with aversive stimuli (Fig. S5K, *P*=0.0100; Table S1). In sum, these results indicate that taste activation broadly does not enhance avoidance of odors.

Subtle sex differences were observed in some measures of volitional behavior (Fig. S6). Females generally spend more time with multisensory stimuli of both valences compared with males (Fig. S6A, *P*=0.0396; Fig. S6L, *P*=0.0098; Table S1). Distance traveled in ROSA (Fig. S6N, *P*=0.0161; Table S1) and depth moved towards the ROSA odor port (Fig. S6P, *P*=0.0156; Table S1) was greater in females solely for the aversive stimulus. On the other hand, bout behaviors between females and males were comparable for stimulus combinations of either valence (Fig. S6G–K,R–V, Table S1). These data suggest that females pursue combined taste and smell more than males, and this manifests for aversive more than appetitive multisensory stimuli.

## DISCUSSION

*Drosophila* is an excellent model to investigate the neural and molecular mechanisms of memory formation. While most studies have focused on classical conditioning in which flies associate an appetitive or aversive unconditioned stimulus, such as sugar or shock, with an odor or visual stimulus (Berry et al., 2008; Davis, 2004; Heisenberg, 2001), flies can also learn operant behaviors necessary to acquire rewards or to avoid negative consequences, which are shaped by environmental outcomes (Baum, 2017; Brembs and Heisenberg, 2000; Brembs and Plendl, 2008; Croteau-Chonka et al., 2022; DeJianne et al., 1985; Ejima and Griffith, 2011; Flores-Valle and Seelig, 2022; Glenn et al., 1992; Neuringer, 2023; Putz et al., 2004; Rajagopalan et al., 2023; Seugnet et al., 2009; Wiggin et al., 2021; Wolf and Heisenberg, 1991). We provide an automated method for quantitative assessment of behavioral nuances underlying operant choice that requires natural behavior in flies and facilitates the study of motivation. Using this Open-LA assay, we show that experience shapes volitional behavior over time by evaluating operant learning and characterizing how flies use distinct bout behaviors for appetitive and aversive stimuli. Our data underscore the importance of moving beyond net behavioral outcomes, such as pursuit or avoidance, to reveal subtle shifts in the types of behaviors flies employ when encountering stimuli.

Consistent with our hypothesis, flies showed an increased preference for ACV, evident by an increase in self-administration throughout the session (Fig. 2). Operant learning occurred early in the session for ACV (Fig. 3), suggesting that flies rapidly learn to pursue appetitive odors. Interestingly, assessment of the operant learning index across stimuli revealed that early learning was greatest for the aversive odor (Fig. 3). Despite a sustained lower preference for the aversive odor compared with the appetitive odor throughout the session (Fig. 2), flies learned to seek the aversive odor over time (Fig. 3). This suggests that BENZ (Liu et al., 1998; Steck et al., 2012; Thum et al., 2007) becomes less aversive with experience, which has implications for odor exposure studies (Guo and Götz, 1997; Keller and Vosshall, 2003; Liu et al., 1998; Semelidou et al., 2019).

Previous work has shown that involuntary brief activation of sweet taste neurons (Gr64f+) is sufficient to induce appetitive multisensory operant matching (Rajagopalan et al., 2023) and induce appetitive responses when paired with consumable stimuli (Jelen et al., 2023). We hypothesized that flies would find voluntary activation of sweet receptor neurons appetitive in an operant context, even in the absence of other stimuli. Our data show that in sated flies, activation of both sweet and bitter taste receptor neurons reduces volitional pursuit of taste across the session compared with ATR– controls (Fig. 4).

The lack of differences observed between volitional activation of sweet versus bitter sensory neurons could be explained by the fact that gustatory sensilla co-house chemosensory and mechanosensory neurons to elicit taste through contact-chemosensation (Gerber et al., 2009). This could suggest that volitional optogenetic activation of *Gr-Gal4* drivers is insufficient to mimic natural taste experience, since gustatory receptor activation does not simultaneously activate mechanosensory neurons. The minimal effect of sweet taste activation on operant learning (Fig. 5) may also stem from flies learning that taste receptor activation does not predict nutrient intake (Gerber et al., 2009). Unlike sweet taste, bitter taste activation increased operant learning throughout the session (Fig. 6). Since bitter taste predicts the presence of toxic substances rather than nutrients, this suggests sweet-related learning may rely more significantly on mechanosensory input, whereas bitter-related learning could rely more on chemosensory activation.

Summative session behaviors alone were not helpful metrics for measuring motivation to receive a taste in sated flies. However, discrete bout behaviors collected throughout the assay demonstrate that flies voluntarily activate sweet taste more than bitter taste. Flies chose to spend more time and move more per bout seeking sweet taste rather than bitter taste (Fig. 4H–K), indicating that behavioral bouts can be used to understand stimulus valence that would be overlooked by assessing session behaviors alone.

Despite this, in our combined taste and smell experiments, sated flies also did not alter their odor-elicited session or bout behaviors upon concurrent sweet activation (Fig. 7). Bitter taste also did not increase aversive odor avoidance, instead, it mildly suppressed time spent with aversive odor and led flies to seek the odor port (Fig. 7). Olfaction, a far-distance sensory modality, primarily functions to direct movement towards or away from an odor source, whereas taste operates at a closer range (Gerber et al., 2009). This likely explains a greater influence of odors on locomotion compared with taste, manifested in a decreased trend in movement observed at the beginning of appetitive multisensory experiments (Fig. 8G) and in distance traveled during aversive multisensory experiments (Fig. 7K), resulting in qualitative rather than quantitative behavioral differences. Only sweet taste enhanced operant learning for the appetitive odor (Fig. 8). This indicates that in the appetitive context, taste does not shape odor response itself but rather the degree of operant learning that occurs.

We suspect that the weaker changes in behavior observed in the multisensory experiments may stem from gustatory receptor neurons functioning as olfactory receptor neurons (Dweck and Carlson, 2023; Wei et al., 2024). Odor activation of taste neurons could induce a ceiling effect, so that additional optogenetic activation of these neurons results in only modest changes in behavior. Recent evidence likewise suggests that benzaldehyde activates Gr66a+ neurons (Wei et al., 2024), which could attenuate the effects of bitter taste on benzaldehyde choice behavior.

Moreover, previous studies demonstrate that behavioral expression of food-associated memory is constrained by satiety (Krashes et al., 2009). Therefore, we speculate that food deprivation will reverse the behavioral trajectory observed in volitional behaviors upon activation of Gr64f+ neurons. We also expect that food deprivation impacts motivational state as opposed to taste perception since food deprivation does not influence activity of Gr5a+ neurons, which co-express Gr64f and serve as its co-receptor in sucrose detection (Dahanukar et al., 2007; Jiao et al., 2008; Kain and Dahanukar, 2015). Given that starvation has consequences for motor control, motivation, stress, memory and the metabolic state of the animal, a broad spectrum of starvation durations (e.g. 12 h, 22 h, 24 h, 48 h) should be examined to fully grasp how starvation impacts volitional behaviors in response to stimuli and shape operant learning across a session (Adel and Griffith, 2021; Fisher et al., 2022; Hernandez et al., 2023; Inagaki et al., 2014, 2012; Musso et al., 2019; Root et al., 2011; Schnaitmann et al., 2010).

Our data also uncovered a potential effect of genetic background or rearing conditions that impacts volitional behavior. *Canton-S* flies reared in 12 h light/dark display increased operant learning in response to appetitive odors (Fig. 3) compared with *Gr64f>CsChrimson* (*w1118* background) ATR– control flies reared in the dark (Fig. 8). This effect is also observed in response to aversive stimuli, since *Canton-S* flies learned more readily to pursue aversive stimuli over time (Fig. 3) compared with *Gr66a>CsChrimson* (*w1118* background) ATR– control flies reared in the dark (Fig. 9). Olfactory antennae respond to light (Krishnan et al., 2001; Senthilan et al., 2012), which could contribute to the differences in behavioral choice of aversive odors. These findings suggest that genetic background and rearing conditions should be considered when investigating operant learning.

Intriguing sex differences emerged in the choices to pursue or avoid appetitive and aversive stimuli, which merit further study. Consistent with previous studies (Anholt et al., 1996; Mackay et al., 1996), we found that males exhibited a stronger avoidance of BENZ than females both in a unisensory (Fig. S2N,P) and multisensory (Fig. S6L,N,P) context. This difference may be attributed to sex-specific variations in the number of olfactory sensilla, with males having 30% more trichoid sensilla and 20% fewer basiconic sensilla compared with females (Stocker, 1994). We also show that females choose to spend more time experiencing sweet and bitter taste and display increased pursuit of multisensory stimuli than males (Fig. S4A,K; Fig. S6A,L,N,P), a phenomenon that could reflect an enhanced innate drive for taste and odor stimuli in females. Interestingly, this pursuit is greater for aversive stimuli. We hypothesize that females choose to explore both multisensory experiences to a greater extent than males partly due to evolutionary pressures linked to reproduction (Billeter and Wolfner, 2018; Camus et al., 2018), such as increased nutrient demands associated with ovary maintenance and the need to avoid unsuitable egg-laying sites that could jeopardize offspring development.

Overall, our findings indicate that while taste influences behavior, odor has a greater impact on volitional choice and operant learning. This is consistent with what is known about fly behavior in the wild. In nature, flies rely on odor plumes during food search to predict food or toxic compounds, and once flies arrive at the odor source, taste receptors help assess food quality (Abu et al., 2018; Chen and Dahanukar, 2020). Although taste activation can be an effective unconditioned stimulus (Haberkern et al., 2019; Rajagopalan et al., 2023), our findings offer new evidence indicating that odor choice reflects valence more effectively than taste activation in sated animals. The Open-LA fills a critical gap in the literature by providing a high-throughput, sensitive operant assay that can be used to characterize how disruptions in circuits and gene expression influence the way that rewards and punishments shape behavioral choices. This provides a promising method for understanding the neural and molecular mechanisms underlying the diversity of individual self-administration of both natural and intoxicating rewards like alcohol and drugs of abuse.

## Supplementary Material

10.1242/jexbio.251074_sup1 Supplementary information
