# Supplementary material for: Shaping of olfactory responses by taste in a new assay for operant learning in Drosophila melanogaster
Source: J Exp Biol. 2025 Dec 19;228(24):jeb251074. doi: 10.1242/jeb.251074 (PMC12766575; doi:10.1242/jeb.251074)
Supplement: Supplementary information [file jexbio-228-251074-s1.pdf]

Table S1. Statistical analysis for experiments conducted.

Supplemental Table 1: Detailed Statistics							
**Figure**	**Experiment Type**	**Comparison Description**	**Statistical test**	**post-hoc P-value**	**Statistic**	**Significance**	**N (# of flies)**
**2A**	Preference Index in **T-maze**	Air vs. 1:100 ACV	One-way ANOVA and post hoc Bonferroni multiple comparisons	p = 0.0275	F (4,108) = 24.38	*	Air n = 22, ACV n = 24, BENZ n = 23
		Air vs. 1:100 BENZ		p < 0.0001		****	
**2D**	OPEN-LA Total **Time spent** in ROSA (Scatter plot, total 15min)	Canton-S Air vs. ACV 15 min	One-Way ANOVA and post hoc Bonferroni multiple comparisons test	p = 0.01	F(2, 213) = 18.27	*	Canton-S ACV n=76, Canton-S BENZ n=74, Canton-S Air n=65
		Canton-S Air vs. BENZ 15min		p = 0.01		*	
		Canton-S ACV vs. BENZ 15min		p < 0.0001		****	
**2E**	OPEN-LA **Preference Index**	Canton-S Air vs. ACV	Kruskall Wallis (statistic = 57.14) and post hoc Dunn's multiple comparisons test	p = 0.01	Z = 2.128	ns	
		Canton-S Air vs. BENZ		p < 0.0001	Z = 4.974	****	
		Canton-S ACV vs. BENZ		p < 0.0001	Z = 7.396	****	
**2F**	OPEN-LA Total **Distance moved** in ROSA (Scatter plot, total 15min)	Canton-S Air vs. ACV 15 min	One-Way ANOVA and post hoc Dunn's multiple comparisons test	p < 0.0001	F(2, 213) = 58.63	****	
		Canton-S Air vs. BENZ 15min		p < 0.0001		****	
		Canton-S ACV vs. BENZ 15min		p > 0.05		ns	
**2G**	OPEN-LA Maximum **Depth** in ROSA (Scatter plot, total 15min)	Canton-S Air vs. ACV 15 min	One-Way ANOVA and post hoc Dunn's multiple comparisons test	p = 0.016	F(2, 213) = 4.416	*	
		Canton-S Air vs. BENZ 15min		p > 0.05		ns	
		Canton-S ACV vs. BENZ 15min		p > 0.05		ns	
**2H**	OPEN-LA **Total entries** into ROSA (Scatter plot, total 15min)	Canton-S Air vs. ACV 15 min	One-Way ANOVA and post hoc Bonferroni multiple comparisons test	p < 0.0001	F(2, 213) = 34.03	****	
		Canton-S Air vs. BENZ 15min		p < 0.0001		****	
		Canton-S ACV vs. BENZ 15min		p = 0.0006		***	
**2I**	OPEN-LA **Bout Duration** in ROSA (Scatter plot, total 15min)	Canton-S Air vs. ACV 15 min	One-Way ANOVA and post hoc Bonferroni multiple comparisons test	p < 0.0001	F(2, 205) = 38.3	****	
		Canton-S Air vs. BENZ 15min		p < 0.0001		****	
		Canton-S ACV vs. BENZ 15min		p = 0.0004		***	
**2J**	OPEN-LA **Occupation duration categories** (Bar graph)	Canton S ACV vs BENZ <2 sec	Two-way ANOVA with Šídák's posthoc comparisons F (7, 1043) = 36.80, p < 0.0001	p = 0.0564	t = 2.764	ns	
		Canton S ACV vs BENZ 2-5 sec		p < 0.0001	t = 6.271	****	
		Canton S ACV vs BENZ 5-10 sec		p < 0.0001	t = 6.995	****	
		Canton S ACV vs BENZ 10-15 sec		p = 0.0006	t = 4.083	***	
		Canton S ACV vs BENZ 15-20 sec		p = 0.0599	t = 2.712	ns	
		Canton S ACV vs BENZ 20-25 sec		p = 0.0001	t = 4.477	***	
		Canton S ACV vs BENZ 25-30 sec		p < 0.0001	t = 5.856	****	
		Canton S ACV vs BENZ 30+ sec		p < 0.0001	t = 10.56	****	
**2K**	OPEN-LA **Bout Distance** in ROSA (Scatter plot, total 15min)	Canton-S Air vs. ACV 15 min	Kruskall Wallis (statistic = 67.57) and post hoc Dunn's multiple comparisons test	p < 0.0001	Z = 7.26	****	
		Canton-S Air vs. BENZ 15min		p > 0.05	Z = 0.131	ns	
		Canton-S ACV vs. BENZ 15min		p < 0.0001	Z = 6.958	****	
**2L**	OPEN-LA **Latency to re-enter** ROSA (Scatter plot, total 15min)	Canton-S Air vs. ACV 15 min	One-Way ANOVA and post hoc Dunn's multiple comparisons test	p < 0.0001	F(2, 198) = 27.04	****	
		Canton-S Air vs. BENZ 15min		p < 0.0001		****	
		Canton-S ACV vs. BENZ 15min		p > 0.05		ns	
**3D**	OPEN-LA Average **Time spent** in ROSA (Line graph)	Canton-S Air vs. ACV 15 min	Mixed-effects model (REML)	p < 0.0001	F (2, 213) = 20.09	****	Canton-S ACV n=76, Canton-S BENZ n=74, Canton-S Air n=65
		Canton-S Air vs. BENZ 15min					
		Canton-S ACV vs. BENZ 15min					
**3E**	OPEN-LA ROSA Operant learning index **Time** (Scatter plot)	Canton-S Air vs. ACV	Kruskall Wallis (statistic = 1.763) and post hoc Dunn's multiple comparisons test	p = 0.8315	Z = 1.087	ns	
		Canton-S Air vs. BENZ		p >0.9999	Z = 0.05946	ns	
		Canton-S ACV vs. BENZ		p = 0.7063	Z = 1.186	ns	
**3F**	OPEN-LA Average **Time spent** in ROSA (Scatter plot, early and late time bin comparison)	Canton-S ACV 0-1min vs 2-3min bin	Wilcoxon matched-pairs signed rank test	p = 0.0083	W = 939	**	
		Canton-S ACV 3-4min vs 13-14min bin		p = 0.0901	W = 520	ns	
**3G**		Canton-S Air 0-1min vs 2-3min bin		p = 0.0852	W = 412	ns	
		Canton-S Air 3-4min vs 13-14min bin		p = 0.3274	W = -100	ns	
**3H**		Canton-S BENZ 0-1min vs 2-3min bin		p < 0.0001	W = 1339	****	
		Canton-S BENZ 3-4min vs 13-14min bin		p = 0.3114	W = -181	ns	
**3I**	OPEN-LA Average **Distance moved** in ROSA (Line graph)	Canton-S Air vs. ACV 15 min	Mixed-effects model (REML)	p < 0.0001	F (2, 213) = 19.16	****	
		Canton-S Air vs. BENZ 15min					
		Canton-S ACV vs. BENZ 15min					
**3J**	OPEN-LA ROSA Operant learning index **Distance** (Scatter plot)	Canton-S Air vs. ACV	Kruskall Wallis (statistic = 15.06) and post hoc Dunn's multiple comparisons test	p = 0.3917	Z = 1.512	ns	
		Canton-S Air vs. BENZ		p = 0.0004	Z = 3.826	***	
		Canton-S ACV vs. BENZ		p = 0.0452	Z = 2.431	*	
**3K**	OPEN-LA Average **Distance moved** in ROSA (Scatter plot, early and late time bin comparison)	Canton-S ACV 0-1min vs 2-3min bin	Wilcoxon matched-pairs signed rank test	p = 0.0787	W = 559	ns	
		Canton-S ACV 3-4min vs 13-14min bin		p = 0.1787	W = -365	ns	
**3L**		Canton-S Air 0-1min vs 2-3min bin		p = 0.4669	W = 26	ns	
		Canton-S Air 3-4min vs 13-14min bin		p = 0.0887	W = -324	ns	
**3M**		Canton-S BENZ 0-1min vs 2-3min bin		p < 0.0001	W = 1839	****	
		Canton-S BENZ 3-4min vs 13-14min bin		p = 0.4978	W = 3	ns	
**3N**	OPEN-LA Average **Depth** in ROSA (Line graph)	Canton-S Air vs. ACV 15 min	Mixed-effects model (REML)	p < 0.0001	F (2, 173) = 15.51	****	
		Canton-S Air vs. BENZ 15min					
		Canton-S ACV vs. BENZ 15min					
**3O**	OPEN-LA ROSA Operant learning index **Depth** (Scatter plot)	Canton-S Air vs. ACV	Kruskall Wallis (statistic = 27.02) and post hoc Dunn's multiple comparisons test	p = 0.2565	Z = 1.72	ns	
		Canton-S Air vs. BENZ		p <0.0001	Z = 5.061	****	
		Canton-S ACV vs. BENZ		p = 0.0014	Z = 3.506	**	
**3P**	OPEN-LA Average **Depth** in ROSA (Scatter plot, early and late time bin comparison)	Canton-S ACV 0-1min vs 2-3min bin	Wilcoxon matched-pairs signed rank test	p < 0.0001	W = 1177	****	
		Canton-S ACV 3-4min vs 13-14min bin		p = 0.3843	W = -79	ns	
**3Q**		Canton-S Air 0-1min vs 2-3min bin		p = 0.2369	W = 216	ns	
		Canton-S Air 3-4min vs 13-14min bin		p = 0.0053	W = -604	**	
**3R**		Canton-S BENZ 0-1min vs 2-3min bin		p < 0.0001	W = 1028	****	
		Canton-S BENZ 3-4min vs 13-14min bin		p = 0.4157	W = 45	ns	
**4C**	OPEN-LA Total **Time spent** in ROSA (Scatter plot, total 15min)	Gr64f Sweet(ATR+) vs. Gr64f No taste(ATR-) 15 min	Mann-Whitney U test	p = 0.0003	U = 1016	***	Gr64f No taste(ATR-) n=56, Gr64f Sweet(ATR+) n=58, Gr66a No taste(ATR-) n=57, Gr66a Bitter(ATR+) n=61
		Gr66a Bitter(ATR+) vs. Gr66a No taste(ATR-) 15 min		p < 0.0001	U = 845	****	
		Gr64f No taste(ATR-) vs Gr66a No taste (ATR-)15min	Mann-Whitney U test	p > 0.05	U = 1437	ns	
		Gr64f Sweet(ATR+) vs. Gr66a Bitter (ATR+) 15min		p > 0.05	U = 1504	ns	
**4D**	OPEN-LA **Preference Index**	Gr64f Sweet(ATR+) vs. Gr64f No taste(ATR-)	Unpaired t test with Welch's correction	p = 0.0259	t=2.258, df=111.1	*	
		Gr66a Bitter(ATR+) vs. Gr66a No taste(ATR-)		p < 0.0001	t=5.166, df=113.8	****	
**4E**	OPEN-LA Total **Distance moved** in ROSA (Scatter plot, total 15min)	Gr64f Sweet(ATR+) vs. Gr64f No taste(ATR-) 15 min	Mann-Whitney U test	p = 0.02	U = 1310	*	
		Gr66a Bitter(ATR+) vs. Gr66a No taste(ATR-) 15 min		p = 0.0019	U = 1166	**	
		Gr64f No taste(ATR-) vs Gr66a No taste (ATR-)15min	Mann-Whitney U test	p = 0.0348	U = 1304	*	
		Gr64f Sweet(ATR+) vs. Gr66a Bitter (ATR+) 15min		p > 0.05	U = 1712	ns	
**4F**	OPEN-LA Total **Depth** in ROSA (Scatter plot, total 15min)	Gr64f Sweet(ATR+) vs. Gr64f No taste(ATR-) 15 min	Mann-Whitney U test	p > 0.05	U = 1534	ns	
		Gr66a Bitter(ATR+) vs. Gr66a No taste(ATR-) 15 min		p = 0.0006	U = 1105	***	
		Gr64f No taste(ATR-) vs Gr66a No taste (ATR-)15min	Mann-Whitney U test	p = 0.0006	U = 1023	***	
		Gr64f Sweet(ATR+) vs. Gr66a Bitter (ATR+) 15min		p > 0.05	U = 1665	ns	
		Gr64f Sweet(ATR+) vs. Gr64f No taste(ATR-) 15 min		p > 0.05	U = 1468	ns	

**4G**	OPEN-LA **Total entries** into ROSA (Scatter plot, total 15min)	Gr66a Bitter(ATR+) vs. Gr66a No taste(ATR-) 15 min		p > 0.05	U = 1694	ns	
		Gr64f No taste(ATR-) vs Gr66a No taste (ATR-)15min	T-test	p > 0.05	t = 1.482, df = 112	ns	
		Gr64f Sweet(ATR+) vs. Gr66a Bitter (ATR+) 15min	Mann-Whitney U test	p > 0.05	U = 1547	ns	
**4H**	OPEN-LA **Bout duration** in ROSA (Scatter plot, total 15min)	Gr64f Sweet(ATR+) vs. Gr64f No taste(ATR-) 15 min	Mann-Whitney U test	p = 0.0055	U = 1159	**	
		Gr66a Bitter(ATR+) vs. Gr66a No taste(ATR-) 15 min		p = 0.0012	U = 1141	**	
		Gr64f No taste(ATR-) vs Gr66a No taste (ATR-)15min	Mann-Whitney U test	p > 0.05	U = 1372	ns	
		Gr64f Sweet(ATR+) vs. Gr66a Bitter (ATR+) 15min		p = 0.0058	U = 1296	**	
**4I**	OPEN-LA **Occupation duration categories** (Bar graph)	Gr64f Sweet(ATR+) vs. Gr66a Bitter (ATR+) <2 sec	Two-way ANOVA with Šídák's posthoc comparisons F (7, 819) = 5.638, P<0.0001	p > 0.05	t = 0.4565	ns	
		Gr64f Sweet(ATR+) vs. Gr66a Bitter (ATR+) 2-5 sec		p > 0.05	t = 1.929	ns	
		Gr64f Sweet(ATR+) vs. Gr66a Bitter (ATR+) 5-10 sec		p = 0.0161	t = 3.177	*	
		Gr64f Sweet(ATR+) vs. Gr66a Bitter (ATR+) 10-15 sec		p = 0.0338	t = 2.924	*	
		Gr64f Sweet(ATR+) vs. Gr66a Bitter (ATR+) 15-20 sec		p > 0.05	t = 1.959	ns	
		Gr64f Sweet(ATR+) vs. Gr66a Bitter (ATR+) 20-25 sec		p = 0.0123	t = 3.246	**	
		Gr64f Sweet(ATR+) vs. Gr66a Bitter (ATR+) 25-30 sec		p > 0.05	t = 2.622	ns	
		Gr64f Sweet(ATR+) vs. Gr66a Bitter (ATR+) 30+ sec		p = 0.0027	t = 3.692	**	
**4J**	OPEN-LA **Bout distance** in ROSA (Scatter plot, total 15min)	Gr64f Sweet(ATR+) vs. Gr64f No taste(ATR-) 15 min	Mann-Whitney U test	p = 0.0083	U = 1183	**	
		Gr66a Bitter(ATR+) vs. Gr66a No taste(ATR-) 15 min		p = 0.0001	U = 1035	***	
		Gr64f No taste(ATR-) vs Gr66a No taste (ATR-)15min	Mann-Whitney U test	p > 0.05	U = 1436	ns	
		Gr64f Sweet(ATR+) vs. Gr66a Bitter (ATR+) 15min		p = 0.011	U = 1338	*	
**4K**	OPEN-LA **Latency to re-enter** ROSA (Scatter plot, total 15min)	Gr64f Sweet(ATR+) vs. Gr64f No taste(ATR-) 15 min	Mann-Whitney U test	p = 0.0055	U = 1159	**	
		Gr66a Bitter(ATR+) vs. Gr66a No taste(ATR-) 15 min		p = 0.0012	U = 1141	**	
		Gr64f No taste(ATR-) vs Gr66a No taste (ATR-)15min	Mann-Whitney U test	p > 0.05	U = 1372	ns	
		Gr64f Sweet(ATR+) vs. Gr66a Bitter (ATR+) 15min		p = 0.0058	U = 1296	**	
**5C**	OPEN-LA Average **Time spent** in ROSA (Line graph)	Gr64f Sweet(ATR+) vs. Gr64f No taste(ATR-) 15 min	Mixed-effects model (REML)	p = 0.0006	F (1, 113) = 12.42	***	Gr64f No taste(ATR-) n=56, Gr64f Sweet(ATR+) n=58
**5D**	OPEN-LA ROSA Operant learning index **Time** (Scatter plot)	Gr64f Sweet(ATR+) vs. Gr64f No taste(ATR-)	Mann-Whitney U test	p = 0.6648	U = 1575	ns	
**5E**	OPEN-LA Average **Time spent** in ROSA (Scatter plot, early and late time bin comparison)	Gr64f No taste(ATR-) 0-1min vs 2-3min bin	Wilcoxon matched-pairs signed rank test	p = 0.078	W = -359	ns	
		Gr64f No taste(ATR-) 3-4min vs 13-14min bin		p = 0.075	W = -318	ns	
**5F**		Gr64f Sweet(ATR+) 0-1min vs 2-3min bin		p = 0.0639	W = -395	ns	
		Gr64f Sweet(ATR+) 3-4min vs 13-14min bin		p = 0.0424	W = -435	*	
**5G**	OPEN-LA Average **Distance moved** in ROSA (Line graph)	Gr64f Sweet(ATR+) vs. Gr64f No taste(ATR-) 15 min	Mixed-effects model (REML)	p = 0.0032	F (1, 113) = 9.080	**	
**5H**	OPEN-LA ROSA Operant learning index **Distance** (Scatter plot)	Gr64f Sweet(ATR+) vs. Gr64f No taste(ATR-)	Mann-Whitney U test	p = 0.4011	U = 1608	ns	
**5I**	OPEN-LA Average **Distance moved** in ROSA (Scatter plot, early and late time bin comparison)	Gr64f No taste(ATR-) 0-1min vs 2-3min bin	Wilcoxon matched-pairs signed rank test	p = 0.001	W = -765	**	
		Gr64f No taste(ATR-) 3-4min vs 13-14min bin		p = 0.0942	W = -316	ns	
**5J**		Gr64f Sweet(ATR+) 0-1min vs 2-3min bin		p = 0.0108	W = -591	*	
		Gr64f Sweet(ATR+) 3-4min vs 13-14min bin		p = 0.0712	W = -371	ns	
**5K**	OPEN-LA Average **Depth** in ROSA (Line graph)	Gr64f Sweet(ATR+) vs. Gr64f No taste(ATR-) 15 min	Mixed-effects model (REML)	p = 0.1634	F (1, 113) = 1.968	ns	
**5L**	OPEN-LA ROSA Operant learning index **Depth** (Scatter plot)	Gr64f Sweet(ATR+) vs. Gr64f No taste(ATR-)	Mann-Whitney U test	p = 0.4916	U = 1649	ns	
**5M**	OPEN-LA Average **Depth** in ROSA (Scatter plot, early and late time bin comparison)	Gr64f No taste(ATR-) 0-1min vs 2-3min bin	Wilcoxon matched-pairs signed rank test	p = 0.2234	W = -164	ns	
		Gr64f No taste(ATR-) 3-4min vs 13-14min bin		p = 0.0303	W = -412	*	
**5N**		Gr64f Sweet(ATR+) 0-1min vs 2-3min bin		p = 0.0302	W = -473	*	
		Gr64f Sweet(ATR+) 3-4min vs 13-14min bin		p = 0.0279	W = -444	*	
**6C**	OPEN-LA Average **Time spent** in ROSA (Line graph)	Gr66a Bitter(ATR+) vs. Gr66a No taste(ATR-) 15 min	Mixed-effects model (REML)	p < 0.0001	F (1, 116) = 29.03	****	Gr66a No taste(ATR-) n=57, Gr66a Bitter(ATR+) n=61
**6D**	OPEN-LA ROSA Operant learning index **Time** (Scatter plot)	Gr66a Bitter(ATR+) vs. Gr66a No taste(ATR-)	Mann-Whitney U test	p = 0.0123	U = 1275	*	
**6E**	OPEN-LA Average **Time spent** in ROSA (Scatter plot, early and late time bin comparison)	Gr66a No taste(ATR-) 0-1min vs 2-3min bin	Wilcoxon matched-pairs signed rank test	p = 0.1208	W = 297	ns	
		Gr66a No taste(ATR-) 3-4min vs 13-14min bin		p = 0.4088	W = 55	ns	
**6F**		Gr66a Bitter(ATR+) 0-1min vs 2-3min bin		p = 0.0417	W = -483	*	
		Gr66a Bitter(ATR+) 3-4min vs 13-14min bin		p = 0.0139	W = -611	*	
**6G**	OPEN-LA Average **Distance moved** in ROSA (Line graph)	Gr66a Bitter(ATR+) vs. Gr66a No taste(ATR-) 15 min	Mixed-effects model (REML)	p < 0.0001	F (1, 116) = 21.57	****	
**6H**	OPEN-LA ROSA Operant learning index **Distance** (Scatter plot)	Gr66a Bitter(ATR+) vs. Gr66a No taste(ATR-)	Mann-Whitney U test	p <0.0001	U = 983	****	
**6I**	OPEN-LA Average **Distance moved** in ROSA (Scatter plot, early and late time bin comparison)	Gr66a No taste(ATR-) 0-1min vs 2-3min bin	Wilcoxon matched-pairs signed rank test	p = 0.084	W = 349	ns	
		Gr66a No taste(ATR-) 3-4min vs 13-14min bin		p = 0.3121	W = 122	ns	
**6J**		Gr66a Bitter(ATR+) 0-1min vs 2-3min bin		p = 0.0278	W = -553	*	
		Gr66a Bitter(ATR+) 3-4min vs 13-14min bin		p = 0.15	W = -291	ns	
**6K**	OPEN-LA Average **Depth** in ROSA (Line graph)	Gr66a Bitter(ATR+) vs. Gr66a No taste(ATR-) 15 min	Mixed-effects model (REML)	p < 0.0001	F (1, 116) = 22.98	****	
**6L**	OPEN-LA ROSA Operant learning index **Depth** (Scatter plot)	Gr66a Bitter(ATR+) vs. Gr66a No taste(ATR-)	Mann-Whitney U test	p = 0.0008	U = 1126	***	
**6M**	OPEN-LA Average **Depth** in ROSA (Scatter plot, early and late time bin comparison)	Gr66a No taste(ATR-) 0-1min vs 2-3min bin	Wilcoxon matched-pairs signed rank test	p = 0.0422	W = 435	*	
		Gr66a No taste(ATR-) 3-4min vs 13-14min bin		p = 0.1253	W = 254	ns	
**6N**		Gr66a Bitter(ATR+) 0-1min vs 2-3min bin		p = 0.2886	W = -153	ns	
		Gr66a Bitter(ATR+) 3-4min vs 13-14min bin		p = 0.0547	W = -404	ns	

Journal of Experimental Biology • Supplementary information

**7C**	OPEN-LA Total **Time spent** in ROSA (Scatter plot, total 15min)	Gr64f ACV+Sweet(ATR+) vs. Gr64f ACV+No taste(ATR-) 15 min	Mann-Whitney U test	p > 0.05	U = 1076	ns	Gr64f ACV+No taste(ATR-) n=47, Gr64f ACV+Sweet(ATR+) n=47, Gr66a BENZ+No taste(ATR-) n=49, Gr66a BENZ+Bitter(ATR+) n=48
		Gr66a BENZ+Bitter(ATR+) vs. Gr66a BENZ+No taste(ATR-) 15 min		p = 0.04	U = 947	*	
		Gr64f ACV+No taste(ATR-) vs. Gr66a BENZ+No taste(ATR-) 15min	Mann-Whitney U test	p < 0.0001	U = 141	****	
		Gr64f ACV+Sweet(ATR+) vs. Gr66a BENZ+Bitter(ATR+) 15min		p < 0.0001	U = 125	****	
**7D**	OPEN-LA **Preference Index**	Gr64f ACV+Sweet(ATR+) vs. Gr64f ACV+No taste(ATR-)	Mann-Whitney U test	p > 0.05	U = 1009	ns	
		Gr66a BENZ+Bitter(ATR+) vs. Gr66a BENZ+No taste(ATR-)		p= 0.0258	U = 906	*	
**7E**	OPEN-LA Total **Distance moved** in ROSA (Scatter plot, total 15min)	Gr64f ACV+Sweet(ATR+) vs. Gr64f ACV+No taste(ATR-) 15 min	Mann-Whitney U test	p > 0.05	U = 927	ns	
		Gr66a BENZ+Bitter(ATR+) vs. Gr66a BENZ+No taste(ATR-) 15 min		p > 0.05	U = 1167	ns	
		Gr64f ACV+No taste(ATR-) vs. Gr66a BENZ+No taste(ATR-) 15min	Mann-Whitney U test	p < 0.0001	U = 438	****	
		Gr64f ACV+Sweet(ATR+) vs. Gr66a BENZ+Bitter(ATR+) 15min		p = 0.006	U = 815	**	
**7F**	OPEN-LA Maximum **Depth** in ROSA (Scatter plot, total 15min)	Gr64f ACV+Sweet(ATR+) vs. Gr64f ACV+No taste(ATR-) 15 min	Mann-Whitney U test	p > 0.05	U = 980	ns	
		Gr66a BENZ+Bitter(ATR+) vs. Gr66a BENZ+No taste(ATR-) 15 min		p = 0.003	U = 797	**	
		Gr64f ACV+No taste(ATR-) vs. Gr66a BENZ+No taste(ATR-) 15min	Mann-Whitney U test	p < 0.0001	U = 435.5	****	
		Gr64f ACV+Sweet(ATR+) vs. Gr66a BENZ+Bitter(ATR+) 15min		p = 0.0007	U = 720	***	
**7G**	OPEN-LA **Total entries** into ROSA (Scatter plot, total 15min)	Gr64f ACV+Sweet(ATR+) vs. Gr64f ACV+No taste(ATR-) 15 min	Mann-Whitney U test	p > 0.05	U = 1025	ns	
		Gr66a BENZ+Bitter(ATR+) vs. Gr66a BENZ+No taste(ATR-) 15 min		p > 0.05	U = 959	ns	
		Gr64f ACV+No taste(ATR-) vs. Gr66a BENZ+No taste(ATR-) 15min	Mann-Whitney U test	p < 0.0001	U = 347.5	****	
		Gr64f ACV+Sweet(ATR+) vs. Gr66a BENZ+Bitter(ATR+) 15min		p < 0.0001	U = 303.5	****	
**7H**	OPEN-LA **Bout duration** in ROSA (Scatter plot, total 15min)	Gr64f ACV+Sweet(ATR+) vs. Gr64f ACV+No taste(ATR-) 15 min	Mann-Whitney U test	p > 0.05	U = 1100	ns	
		Gr66a BENZ+Bitter(ATR+) vs. Gr66a BENZ+No taste(ATR-) 15 min		p > 0.05	U = 1130	ns	
		Gr64f ACV+No taste(ATR-) vs. Gr66a BENZ+No taste(ATR-) 15min	Mann-Whitney U test	p < 0.0001	U = 82	****	
		Gr64f ACV+Sweet(ATR+) vs. Gr66a BENZ+Bitter(ATR+) 15min		p < 0.0001	U = 70	****	
**7I**	OPEN-LA **Occupation duration categories** (Bar graph)	Gr64f ACV+Sweet(ATR+) vs. Gr64f ACV+Sweet(ATR-) <2 sec	Two-way ANOVA with Šídák's posthoc comparisons F(7, 644) = 1.912, p = 0.0652	p = 0.0148	t = 3.123	*	
		Gr64f ACV+Sweet(ATR+) vs. Gr64f ACV+Sweet(ATR-) 2-5 sec		p > 0.9999	t = 0.6060	ns	
		Gr64f ACV+Sweet(ATR+) vs. Gr64f ACV+Sweet(ATR-) 5-10 sec		p > 0.9135	t = 1.119	ns	
		Gr64f ACV+Sweet(ATR+) vs. Gr64f ACV+Sweet(ATR-) 10-15 sec		p > 0.9886	t = .7925	ns	
		Gr64f ACV+Sweet(ATR+) vs. Gr64f ACV+Sweet(ATR-) 15-20 sec		p > 0.9999	t = .3263	ns	
		Gr64f ACV+Sweet(ATR+) vs. Gr64f ACV+Sweet(ATR-) 20-25 sec		p > 0.9999	t = 0.09323	ns	
		Gr64f ACV+Sweet(ATR+) vs. Gr64f ACV+Sweet(ATR-) 25-30 sec		p > 0.9999	t = 0.2797	ns	
		Gr64f ACV+Sweet(ATR+) vs. Gr64f ACV+Sweet(ATR-) 30+ sec		p > 0.9135	t = 1.119	ns	
**7J**	OPEN-LA **Occupation duration categories** (Bar graph)	Gr66a BENZ+Bitter(ATR+) vs. Gr66a BENZ+No Taste(ATR-) <2 sec	Two-way ANOVA with Bonferroni posthoc comparisons F(7, 665) = 7.171, p < 0.0001	p = 0.0032	t = 3.697	**	
		Gr66a BENZ+Bitter(ATR+) vs. Gr66a BENZ+No Taste(ATR-) 2-5 sec		p = 0.1718	t = 2.345	ns	
		Gr66a BENZ+Bitter(ATR+) vs. Gr66a BENZ+No TasteATR-) 5-10 sec		p = 0.2378	t = 2.213	ns	
		Gr66a BENZ+Bitter(ATR+) vs. Gr66a BENZ+No Taste(ATR-) 10-15 sec		p = 0.9234	t = 1.589	ns	
		Gr66a BENZ+Bitter(ATR+) vs. Gr66a BENZ+No Taste(ATR-) 15-20 sec		p > 0.9999	t = 1.292	ns	
		Gr66a BENZ+Bitter(ATR+) vs. Gr66a BENZ+No Taste(ATR-) 20-25 sec		p = 0.0367	t = 2.911	*	
		Gr66a BENZ+Bitter(ATR+) vs. Gr66a BENZ+No Taste(ATR-) 25-30 sec		p = 0.1148	t = 2.511	ns	
		Gr66a BENZ+Bitter(ATR+) vs. Gr66a BENZ+No Taste(ATR-) 30+ sec		p > 0.9999	t = 0.2058	ns	
**7K**	OPEN-LA **Bout distance** in ROSA (Scatter plot, total 15min)	Gr64f ACV+Sweet(ATR+) vs. Gr64f ACV+No taste(ATR-) 15 min	Mann-Whitney U test	p > 0.05	U = 1060	ns	
		Gr66a BENZ+Bitter(ATR+) vs. Gr66a BENZ+No taste(ATR-) 15 min		p < 0.0001	U = 1035	****	
		Gr64f ACV+No taste(ATR-) vs. Gr66a BENZ+No taste(ATR-) 15min	Mann-Whitney U test	p < 0.0001	U = 70	****	
		Gr64f ACV+Sweet(ATR+) vs. Gr66a BENZ+Bitter(ATR+) 15min		p < 0.0001	U = 79	****	
**7L**	OPEN-LA **Latency to re-enter** ROSA (Scatter plot, total 15min)	Gr64f ACV+Sweet(ATR+) vs. Gr64f ACV+No taste(ATR-) 15 min	Mann-Whitney U test	p > 0.05	U = 1100	ns	
		Gr66a BENZ+Bitter(ATR+) vs. Gr66a BENZ+No taste(ATR-) 15 min		p > 0.05	U = 1130	ns	
		Gr64f ACV+No taste(ATR-) vs. Gr66a BENZ+No taste(ATR-) 15min	Mann-Whitney U test	p < 0.0001	U = 106	****	
		Gr64f ACV+Sweet(ATR+) vs. Gr66a BENZ+Bitter(ATR+) 15min		p < 0.0001	U = 82	****	
**8C**	OPEN-LA Average **Time spent** in ROSA (Line graph)	Gr64f ACV+Sweet(ATR+) vs. Gr64f ACV+No taste(ATR-) 15 min	Mixed-effects model (REML)	p > 0.05	F (1, 91) = 0.06389	ns	
**8D**	OPEN-LA ROSA Operant learning index **Time** (Scatter plot)	Gr64f ACV+Sweet(ATR+) vs. Gr64f ACV+No taste(ATR-)	Mann-Whitney U test	p = 0.0037	U = 752	**	
**8E**	OPEN-LA Average **Time spent** in ROSA (Scatter plot, early and late time bin comparison)	Gr64f ACV+No taste(ATR-) 0-1min vs 2-3min bin	Wilcoxon matched-pairs signed rank test	p = 0.3018	W = 100	ns	
		Gr64f ACV+No taste(ATR-) 3-4min vs 13-14min bin		p = 0.1001	W = -236	ns	
**8F**		Gr64f ACV+Sweet(ATR+) 0-1min vs 2-3min bin		p = 0.3208	W = 87	ns	
		Gr64f ACV+Sweet(ATR+) 3-4min vs 13-14min bin		p = 0.0765	W = 255	ns	
**8G**	OPEN-LA Average **Distance moved** in ROSA (Line graph)	Gr64f ACV+Sweet(ATR+) vs. Gr64f ACV+No taste(ATR-) 15 min	Mixed-effects model (REML)	p > 0.05	F (1, 92) = 1.647	ns	

Journal of Experimental Biology • Supplementary information

**8H**	OPEN-LA ROSA Operant learning index **Distance** (Scatter plot)	Gr64f ACV+Sweet(ATR+) vs. Gr64f ACV+No taste(ATR-)	Mann-Whitney U test	p = 0.1119	U = 943	ns	Gr64f ACV+No taste(ATR-) n=47, Gr64f ACV+Sweet(ATR+) n=47
**8I**	OPEN-LA Average **Distance moved** in ROSA (Scatter plot, early and late time bin comparison)	Gr64f ACV+No taste(ATR-) 0-1min vs 2-3min bin	Wilcoxon matched-pairs signed rank test	p = 0.1551	W = 194	ns	
		Gr64f ACV+No taste(ATR-) 3-4min vs 13-14min bin		p = 0.1452	W = -202	ns	
**8J**		Gr64f ACV+Sweet(ATR+) 0-1min vs 2-3min bin		p = 0.313	W = 91	ns	
		Gr64f ACV+Sweet(ATR+) 3-4min vs 13-14min bin		p = 0.3605	W = 67	ns	
**8K**	OPEN-LA Average **Depth** in ROSA (Line graph)	Gr64f ACV+Sweet(ATR+) vs. Gr64f ACV+No taste(ATR-) 15 min	Mixed-effects model (REML)	p > 0.05	F (1, 92) = 0.7741	ns	
**8L**	OPEN-LA ROSA Operant learning index **Depth** (Scatter plot)	Gr64f ACV+Sweet(ATR+) vs. Gr64f ACV+No taste(ATR-)	Mann-Whitney U test	p = 0.0068	U = 779.5	**	
**8M**	OPEN-LA Average **Depth** in ROSA (Scatter plot, early and late time bin comparison)	Gr64f ACV+No taste(ATR-) 0-1min vs 2-3min bin	Wilcoxon matched-pairs signed rank test	p = 0.1782	W = 165	ns	
		Gr64f ACV+No taste(ATR-) 3-4min vs 13-14min bin		p = 0.3796	W = 50	ns	
**8N**		Gr64f ACV+Sweet(ATR+) 0-1min vs 2-3min bin		p = 0.165	W = 163	ns	
		Gr64f ACV+Sweet(ATR+) 3-4min vs 13-14min bin		p = 0.0005	W = 512	***	
**9C**	OPEN-LA Average **Time spent** in ROSA (Line graph)	Gr66a BENZ+Bitter(ATR+) vs. Gr66a BENZ+No taste(ATR-) 15 min	Mixed-effects model (REML)	p > 0.05	F (1, 95) = 1.536	ns	Gr66a BENZ+No taste(ATR-) n=49, Gr66a BENZ+Bitter(ATR+) n=48
**9D**	OPEN-LA ROSA Operant learning index **Time** (Scatter plot)	Gr66a BENZ+Bitter(ATR+) vs. Gr66a BENZ+No taste(ATR-)	Mann-Whitney U test	p = 0.1316	U = 1020	ns	
**9E**	OPEN-LA Average **Time spent** in ROSA (Scatter plot, early and late time bin comparison)	Gr66a BENZ+No Taste(ATR-) 0-1min vs 2-3min bin	Wilcoxon matched-pairs signed rank test	p = 0.0076	W = 470	**	
		Gr66a BENZ+No Taste(ATR-) 3-4min vs 13-14min bin		p = 0.0035	W = 504	**	
**9F**		Gr66a BENZ+Bitter(ATR+) 0-1min vs 2-3min bin		p = 0.0184	W = 419	*	
		Gr66a BENZ+Bitter(ATR+) 3-4min vs 13-14min bin		p = 0.1339	W = 225	ns	
**9G**	OPEN-LA Average **Distance moved** in ROSA (Line graph)	Gr66a BENZ+Bitter(ATR+) vs. Gr66a BENZ+No taste(ATR-) 15 min	Mixed-effects model (REML)	p > 0.05	F (1, 95) = 0.5085	ns	
**9H**	OPEN-LA ROSA Operant learning index **Distance** (Scatter plot)	Gr66a BENZ+Bitter(ATR+) vs. Gr66a BENZ+No taste(ATR-)	Mann-Whitney U test	p = 0.1229	U = 1015	ns	
**9I**	OPEN-LA Average **Distance moved** in ROSA (Scatter plot, early and late time bin comparison)	Gr66a BENZ+No Taste(ATR-) 0-1min vs 2-3min bin	Wilcoxon matched-pairs signed rank test	p = 0.1036	W = 248	ns	
		Gr66a BENZ+No Taste(ATR-) 3-4min vs 13-14min bin		p = 0.1452	W = 202	ns	
**9J**		Gr66a BENZ+Bitter(ATR+) 0-1min vs 2-3min bin		p = 0.1318	W = 227	ns	
		Gr66a BENZ+Bitter(ATR+) 3-4min vs 13-14min bin		p = 0.0938	W = 267	ns	
**9K**	OPEN-LA Average **Depth** in ROSA (Line graph)	Gr66a BENZ+Bitter(ATR+) vs. Gr66a BENZ+No taste(ATR-) 15 min	Mixed-effects model (REML)	p > 0.05	F (1, 95) = 2.875	ns	
**9L**	OPEN-LA ROSA Operant learning index **Depth** (Scatter plot)	Gr66a BENZ+Bitter(ATR+) vs. Gr66a BENZ+No taste(ATR-)	Mann-Whitney U test	p = 0.2442	U = 1287	ns	
**9M**	OPEN-LA Average **Depth** in ROSA (Scatter plot, early and late time bin comparison)	Gr66a BENZ+No Taste(ATR-) 0-1min vs 2-3min bin	Wilcoxon matched-pairs signed rank test	p = 0.1172	W = 234	ns	
		Gr66a BENZ+No Taste(ATR-) 3-4min vs 13-14min bin		p = 0.029	W = 358	*	
**9N**		Gr66a BENZ+Bitter(ATR+) 0-1min vs 2-3min bin		p = 0.2518	W = -136	ns	
		Gr66a BENZ+Bitter(ATR+) 3-4min vs 13-14min bin		p = 0.0498	W = 322	*	
**S1C**	OPEN-LA Average **Time spent** in NoSA (Line graph)	Canton-S Air vs. ACV 15 min	Mixed-effects model (REML)	p < 0.0001	F (2, 213) = 19.16	****	Canton-S ACV n=76, Canton-S BENZ n=74, Canton-S Air n=65
		Canton-S Air vs. BENZ 15min					
		Canton-S ACV vs. BENZ 15min					
**S1D**	OPEN-LA Total **Time spent** in NoSA (Scatter plot, total 15min)	Canton-S Air vs. ACV 15 min	One-way ANOVA; F (2, 213) = 18.09	p < 0.0001	t = 3.454	**	
		Canton-S Air vs. BENZ 15min		p = 0.0677	t = 2.297	ns	
		Canton-S ACV vs. BENZ 15min		p < 0.0001	t = 5.973	****	
**S1E**	OPEN-LA Average **Distance moved** in NoSA (Line graph)	Canton-S Air vs. ACV 15 min	Mixed-effects model (REML)	p = 0.0002	F (2, 213) = 8.819	***	
		Canton-S Air vs. BENZ 15min					
		Canton-S ACV vs. BENZ 15min					
**S1F**	OPEN-LA Total **Distance moved** in NoSA (Scatter plot, total 15min)	Canton-S Air vs. ACV 15 min	Kruskal-Wallis (statistic = 110.5, p < 0.0001) with Dunn's posthoc multiple comparisons	p < 0.0001	Z = 4.678	****	
		Canton-S Air vs. BENZ 15min		p < 0.0001	Z = 10.45	****	
		Canton-S ACV vs. BENZ 15min		p < 0.0001	Z = 6.076	****	
**S1G**	OPEN-LA Average **Depth** in NoSA (Line graph)	Canton-S Air vs. ACV 15 min	Mixed-effects model (REML)	p < 0.0001	F (2, 173) = 41.49	****	
		Canton-S Air vs. BENZ 15min					
		Canton-S ACV vs. BENZ 15min					
**S1H**	OPEN-LA **Maximum Depth** in NoSA (Scatter plot, total 15min)	Canton-S Air vs. ACV 15 min	Kruska-Wallis (statistic = 28.13) with Dunn's posthoc multiple comparisons	p = 0.0678	Z = 2.28	**	
		Canton-S Air vs. BENZ 15min		p < 0.0001	Z = 5.264	****	
		Canton-S ACV vs. BENZ 15min		p = 0.0051	Z = 3.138	*	
**S1I**	OPEN-LA **Bout Duration** in NoSA (Scatter plot, total 15min)	Canton-S Air vs. ACV 15 min	Kruskal-Wallis (statistic = 40.80) with Dunn's posthoc multiple comparisons	p = 0.1346	Z = 2.006	ns	
		Canton-S Air vs. BENZ 15min		p < 0.0001	Z = 4.428	****	
		Canton-S ACV vs. BENZ 15min		p < 0.0001	Z = 6.311	****	
**S1J**	OPEN-LA **Bout Distance** in NoSA (Scatter plot, total 15min)	Canton-S Air vs. ACV 15 min	Kruskal-Wallis (statistic = 12.88) with Dunn's posthoc multiple comparisons	p = 0.0012	Z = 3.54	**	
		Canton-S Air vs. BENZ 15min		p = 0.4849	Z = 1.4	ns	
		Canton-S ACV vs. BENZ 15min		p = 0.0792	Z = 2.22	ns	
**S1K**	OPEN-LA Total **Time spent** in ROSA (Scatter plot, total 15min)	Canton-S ACV 100% concentration vs. 1.5% concentration 15 min	Mann-Whitney U test	p = 0.4232	U = 92	ns	Canton S ACV 100% n = 16, ACV 1.5% n = 14
**S1L**	OPEN-LA **Bout Duration** in ROSA (Scatter plot, total 15min)	Canton-S ACV 100% concentration vs. 1.5% concentration 15 min	Mann-Whitney U test	p = 0.0832	U = 64	ns	
**S1M**	OPEN-LA **Latency to re-enter** ROSA (Scatter plot)	Canton-S ACV 100% concentration vs. 1.5% concentration 15 min	Mann-Whitney U test	p = 0.1934	U = 80	ns	
**S1N**	OPEN-LA **total entries** into ROSA (Scatter plot)	Canton-S ACV 100% concentration vs. 1.5% concentration 15 min	Mann-Whitney U test	p = 0.1074	U = 73	ns	
**S1O**	OPEN-LA Total **Time spent** in ROSA (Scatter plot, total 15min)	Canton-S BENZ 1.5% concentration vs. 0.3% concentration 15 min	Mann-Whitney U test	p = 0.6204	U = 607	ns	Canton S BENZ 1.5% n = 29,BENZ 0.3% n = 45
**S1P**	OPEN-LA **Bout Duration** in ROSA (Scatter plot, total 15min)	Canton-S BENZ 1.5% concentration vs. 0.3% concentration 15 min	Mann-Whitney U test	p = 0.0523	U = 477	ns	
**S1Q**	OPEN-LA **Latency to re-enter** ROSA (Scatter plot)	Canton-S BENZ 1.5% concentration vs. 0.3% concentration 15 min	Mann-Whitney U test	p = 0.9824	U = 650	ns	
**S1R**	OPEN-LA **total entries** into ROSA (Scatter plot)	Canton-S BENZ 1.5% concentration vs. 0.3% concentration 15 min	Mann-Whitney U test	p = 0.4652	U = 586	ns	
**S2A**	OPEN-LA Average **Time spent** in ROSA (Line graph)	Canton-S ACV females vs. males 15 min		p = 0.9316	F (1, 75) = 0.007408	ns	

Journal of Experimental Biology • Supplementary information

**S2B**	OPEN-LA Average **Time spent** in NoSA (Line graph)	Canton-S ACV females vs. males 15 min	Mixed-effects model (REML)	p = 0.9317	F (1, 75) = 0.007386	ns	Canton-S ACV females n = 38, males n = 39
**S2C**	OPEN-LA Average **Distance moved** in ROSA (Line graph)	Canton-S ACV females vs. males 15 min		p = 0.0075	F (1, 75) = 7.560	**	
**S2D**	OPEN-LA Average **Distance moved** in NoSA (Line graph)	Canton-S ACV females vs. males 15 min		p = 0.1837	F (1, 75) = 1.801	ns	
**S2E**	OPEN-LA Average **Depth** in ROSA (Line graph)	Canton-S ACV females vs. males 15 min		p = 0.0384	F (1,75) = 4.444	*	
**S2F**	OPEN-LA Average **Depth** in NoSA (Line graph)	Canton-S ACV females vs. males 15 min		p = 0.1159	F (1, 75) = 2.530	ns	
**S2G**	OPEN-LA **Bout duration** in ROSA (Scatter plot)	Canton-S ACV females vs. males 15 min	Mann-Whitney U test	p = 0.7343	U = 707	ns	
	OPEN-LA **Bout duration** in NoSA (Scatter plot)	Canton-S ACV females vs. males 15 min		p = 0.1683	U = 605	ns	
**S2H**	OPEN-LA **Bout distance** in ROSA (Scatter plot)	Canton-S ACV females vs. males 15 min		p = 0.1416	U = 596	ns	
	OPEN-LA **Bout distance** in NoSA (Scatter plot)	Canton-S ACV females vs. males 15 min		p = 0.7884	U = 714	ns	
**S2I**	OPEN-LA **Latency to re-enter** ROSA (Scatter plot)	Canton-S ACV females vs. males 15 min		p = 0.0299	U = 455	*	
**S2J**	OPEN-LA **<2 sec odor sampling** in ROSA (Scatter plot)	Canton-S ACV females vs. males 15 min		p = 0.3204	U = 227	ns	
**S2K**	OPEN-LA **total entries** into ROSA (Scatter plot)	Canton-S ACV females vs. males 15 min		p = 0.8892	U = 727	ns	
**S2L**	OPEN-LA Average **Time spent** in ROSA (Line graph)	Canton-S BENZ females vs. males 15 min	Mixed-effects model (REML)	p = 0.2153	F (1, 72) = 1.563	ns	Canton-S BENZ females n = 36, males n = 38
**S2M**	OPEN-LA Average **Time spent** in NoSA (Line graph)	Canton-S BENZ females vs. males 15 min		p = 0.2227	F (1, 72) = 1.513	ns	
**S2N**	OPEN-LA Average **Distance moved** in ROSA (Line graph)	Canton-S BENZ females vs. males 15 min		p = 0.0271	F (1, 72) = 5.087	*	
**S2O**	OPEN-LA Average **Distance moved** in NoSA (Line graph)	Canton-S BENZ females vs. males 15 min		p = 0.7454	F (1, 72) = 0.1063	ns	
**S2P**	OPEN-LA Average **Depth** in ROSA (Line graph)	Canton-S BENZ females vs. males 15 min		p = 0.0208	F (1, 72) = 5.087	*	
**S2Q**	OPEN-LA Average **Depth** in NoSA (Line graph)	Canton-S BENZ females vs. males 15 min		p = 0.904	F (1, 72) = 0. 01465	ns	
**S2R**	OPEN-LA **Bout duration** in ROSA (Scatter plot)	Canton-S BENZ females vs. males 15 min	Mann-Whitney U test	p = 0.5706	U = 2157	ns	
	OPEN-LA **Bout duration** in NoSA (Scatter plot)	Canton-S BENZ females vs. males 15 min		p = 0.9309	U = 2134	ns	
**S2S**	OPEN-LA **Bout distance** in ROSA (Scatter plot)	Canton-S BENZ females vs. males 15 min		p = 0.0049	U = 426	**	
	OPEN-LA **Bout distance** in NoSA (Scatter plot)	Canton-S BENZ females vs. males 15 min		p = 0.8508	U = 666	ns	
**S2T**	OPEN-LA **Latency to re-enter** ROSA (Scatter plot)	Canton-S BENZ females vs. males 15 min		p = 0.3818	U = 569	ns	
**S2U**	OPEN-LA **<2 sec odor sampling** in ROSA (Scatter plot)	Canton-S BENZ females vs. males 15 min		p = 0.3661	U = 1172	ns	
**S2V**	OPEN-LA **total entries** into ROSA (Scatter plot)	Canton-S BENZ females vs. males 15 min		p = 0.009	U = 447	**	
**S3A**	OPEN-LA Average **Time spent** in NoSA (Line graph)	Gr64f Sweet(ATR+) vs. Gr64f No taste(ATR-) 15 min	Mixed-effects model (REML)	p = 0.0006	F (1, 113) = 12.41	***	Gr64f No taste(ATR-) n=56, Gr64f Sweet(ATR+) n=58, Gr66a No taste(ATR-) n=57, Gr66a Bitter(ATR+) n=61
**S3B**	OPEN-LA Average **Time spent** in NoSA (Line graph)	Gr66a Bitter(ATR+) vs. Gr66a No taste(ATR-) 15 min	Mixed-effects model (REML)	p < 0.0001	F (1, 116) = 28.81	****	
**S3C**	OPEN-LA Total **Time spent** in NoSA (Scatter plot, total 15min)	Gr64f Sweet(ATR+) vs. Gr64f No taste(ATR-) 15 min	Mann-Whitney U test	p < 0.0001	U = 1012	****	
		Gr66a Bitter(ATR+) vs. Gr66a No taste(ATR-) 15 min		p < 0.0001	U = 848	****	
		Gr64f No taste(ATR-) vs Gr66a No taste (ATR-)15min	Unpaired t-test	p = 0.1274	t (1, 112) = 1.536	ns	
		Gr64f Sweet(ATR+) vs. Gr66a Bitter (ATR+) 15min	unpaired t-test	p = 0.0604	t (1, 117) = 1.562	ns	
**S3D**	OPEN-LA Average **Distance moved** in NoSA (Line graph)	Gr64f Sweet(ATR+) vs. Gr64f No taste(ATR-) 15 min	Mixed-effects model (REML)	p = 0.0305	F (1, 113) = 4.802	*	
**S3E**	OPEN-LA Average **Distance moved** in NoSA (Line graph)	Gr66a Bitter(ATR+) vs. Gr66a No taste(ATR-) 15 min	Mixed-effects model (REML)	p = 0.0005	F (1, 116) = 12.99	***	
**S3F**	OPEN-LA Total **Distance moved** in NoSA (Scatter plot, total 15min)	Gr64f Sweet(ATR+) vs. Gr64f No taste(ATR-) 15 min	Unpaired t-test	p = 0.0078	t (1, 112) = 2.708	**	
		Gr66a Bitter(ATR+) vs. Gr66a No taste(ATR-) 15 min	Mann-Whitney U test	p = 0.034	U = 1345	*	
		Gr64f No taste(ATR-) vs Gr66a No taste (ATR-)15min	Unpaired t-test	p = 0.0588	t (1, 112) = 1.909	ns	
		Gr64f Sweet(ATR+) vs. Gr66a Bitter (ATR+) 15min	Unpaired t-test	p = 0.0028	t (1, 117) = 3.054	**	
**S3G**	OPEN-LA Average **Depth** in NoSA (Line graph)	Gr64f Sweet(ATR+) vs. Gr64f No taste(ATR-) 15 min	Mixed-effects model (REML)	p = 0.0538	F (1, 113) = 3.799	ns	
**S3H**	OPEN-LA Average **Depth** in NoSA (Line graph)	Gr66a Bitter(ATR+) vs. Gr66a No taste(ATR-) 15 min	Mixed-effects model (REML)	p = 0.0586	F (1, 116) = 3.648	ns	
**S3I**	OPEN-LA Total **Depth** in NoSA (Scatter plot, total 15min)	Gr64f Sweet(ATR+) vs. Gr64f No taste(ATR-) 15 min	Mann-Whitney U test	p = 0.9223	U = 1635	ns	
		Gr66a Bitter(ATR+) vs. Gr66a No taste(ATR-) 15 min	Mann-Whitney U test	p = 0.1842	U = 1491	ns	
		Gr64f No taste(ATR-) vs Gr66a No taste (ATR-)15min	Mann-Whitney U test	p < 0.0001	U = 963	***	
		Gr64f Sweet(ATR+) vs. Gr66a Bitter (ATR+) 15min		p = 0.0014	U = 1173	***	
**S3J**	OPEN-LA **Bout duration** in NoSA (Scatter plot, total 15min)	Gr64f Sweet(ATR+) vs. Gr64f No taste(ATR-) 15 min	Mann-Whitney U test	p = 0.1156	U = 1438	ns	
		Gr66a Bitter(ATR+) vs. Gr66a No taste(ATR-) 15 min		p = 0.0123	U = 1275	*	
		Gr64f No taste(ATR-) vs Gr66a No taste (ATR-)15min	Mann-Whitney U test	p = 0.0726	t (1, 112) = 1.812	ns	
		Gr64f Sweet(ATR+) vs. Gr66a Bitter (ATR+) 15min		p = 0.537	U = 1652	ns	
**S3K**	OPEN-LA **Bout distance** in NoSA (Scatter plot, total 15min)	Gr64f Sweet(ATR+) vs. Gr64f No taste(ATR-) 15 min	Mann-Whitney U test	p = 0.4203	U =1508	ns	
		Gr66a Bitter(ATR+) vs. Gr66a No taste(ATR-) 15 min		p = 0.0074	U = 1243	**	
		Gr64f No taste(ATR-) vs Gr66a No taste (ATR-)15min	Mann-Whitney U test	p = 0.1799	U = 1387	ns	
		Gr64f Sweet(ATR+) vs. Gr66a Bitter (ATR+) 15min		p = 0.6892	U = 1693	ns	
**S4A**	OPEN-LA Average **Time spent** in ROSA (Line graph)	Gr64f Sweet(ATR+) females vs. males 15 min		p = 0.0026	F (1, 56) = 9.929	**	
**S4B**	OPEN-LA Average **Time spent** in NoSA (Line graph)	Gr64f Sweet(ATR+) females vs. males 15 min		p = 0.0031	F (1, 56) = 9.531	**	

Journal of Experimental Biology • Supplementary information

**S4C**	OPEN-LA Average **Distance moved** in ROSA (Line graph)	Gr64f Sweet(ATR+) females vs. males 15 min	Mixed-effects model (REML)	p = 0.4264	F (1, 56) = 0.6420	ns	Gr64f Sweet(ATR+) females n = 25, males n = 22
**S4D**	OPEN-LA Average **Distance moved** in NoSA (Line graph)	Gr64f Sweet(ATR+) females vs. males 15 min		p = 0.334	F (1, 56) = 0.9495	ns	
**S4E**	OPEN-LA Average **Depth** in ROSA (Line graph)	Gr64f Sweet(ATR+) females vs. males 15 min		p = 0.2876	F (1, 56) = 1.153	ns	
**S4F**	OPEN-LA Average **Depth** in NoSA (Line graph)	Gr64f Sweet(ATR+) females vs. males 15 min		p = 0.6376	F (1, 56) = 0.2244	ns	
**S4G**	OPEN-LA **Bout duration** in ROSA (Scatter plot)	Gr64f Sweet(ATR+) females vs. males 15 min	Mann-Whitney U test	p = 0.0697	U = 306	ns	
	OPEN-LA **Bout duration** in NoSA (Scatter plot)	Gr64f Sweet(ATR+) females vs. males 15 min		p = 0.0045	U = 252	**	
**S4H**	OPEN-LA **Bout distance** in ROSA (Scatter plot)	Gr64f Sweet(ATR+) females vs. males 15 min		p = 0.3844	U = 399	ns	
	OPEN-LA **Bout distance** in NoSA (Scatter plot)	Gr64f Sweet(ATR+) females vs. males 15 min		p = 0.0418	U = 307	*	
**S4I**	OPEN-LA **Latency to re-enter** ROSA (Scatter plot)	Gr64f Sweet(ATR+) females vs. males 15 min		p = 0.0697	U = 323	ns	
**S4J**	OPEN-LA **total entries** into ROSA (Scatter plot)	Gr64f Sweet(ATR+) females vs. males 15 min		p = 0.0043	U = 251	**	
**S4K**	OPEN-LA Average **Time spent** in ROSA (Line graph)	Gr66a Bitter(ATR+) females vs. males 15 min	Mixed-effects model (REML)	p < 0.0001	F (1, 59) = 37.81	****	Gr66a Bitter(ATR+) females n = 22, males n = 27
**S4L**	OPEN-LA Average **Time spent** in NoSA (Line graph)	Gr66a Bitter(ATR+) females vs. males 15 min		p = 0.7425	F (1, 59) = 0.1090	ns	
**S4M**	OPEN-LA Average **Distance moved** in ROSA (Line graph)	Gr66a Bitter(ATR+) females vs. males 15 min		p = 0.7389	F (1, 59) = 0.1121	ns	
**S4N**	OPEN-LA Average **Distance moved** in NoSA (Line graph)	Gr66a Bitter(ATR+) females vs. males 15 min		p = 0.1308	F (1, 59) = 2.348	ns	
**S4O**	OPEN-LA Average **Depth** in ROSA (Line graph)	Gr66a Bitter(ATR+) females vs. males 15 min		p = 0.1528	F (1, 59) = 2.098	ns	
**S4P**	OPEN-LA Average **Depth** in NoSA (Line graph)	Gr66a Bitter(ATR+) females vs. males 15 min		p = 0.6636	F (1, 59) = 0.1911	ns	
**S4Q**	OPEN-LA **Bout duration** in ROSA (Scatter plot)	Gr66a Bitter(ATR+) females vs. males 15 min	Mann-Whitney U test	p = 0.2857	U = 425	ns	
	OPEN-LA **Bout duration** in NoSA (Scatter plot)	Gr66a Bitter(ATR+) females vs. males 15 min		p = 0.0373	U = 341	*	
**S4R**	OPEN-LA **Bout distance** in ROSA (Scatter plot)	Gr66a Bitter(ATR+) females vs. males 15 min		p = 0.3791	U = 443	ns	
	OPEN-LA **Bout distance** in NoSA (Scatter plot)	Gr66a Bitter(ATR+) females vs. males 15 min		p = 0.0465	U = 348	*	
**S4S**	OPEN-LA **Latency to re-enter** ROSA (Scatter plot)	Gr66a Bitter(ATR+) females vs. males 15 min		p = 0.2857	U = 425	ns	
**S4T**	OPEN-LA **total entries** into ROSA (Scatter plot)	Gr66a Bitter(ATR+) females vs. males 15 min		p > 0.05	U = 373	ns	
**S5A**	OPEN-LA Average **Time spent** in NoSA (Line graph)	Gr64f ACV+Sweet(ATR+) vs. Gr64f ACV+No taste(ATR-) 15 min	Mixed-effects model (REML)	p = 0.9335	F (1, 92) = 0.007011	ns	Gr64f ACV+No taste(ATR-) n=47, Gr64f ACV+Sweet(ATR+) n=47, Gr66a BENZ+No taste(ATR-) n=49, Gr66a BENZ+Bitter(ATR+) n=48
**S5B**	OPEN-LA Average **Time spent** in NoSA (Line graph)	Gr66a BENZ+Bitter(ATR+) vs. Gr66a BENZ+No taste(ATR-) 15 min	Mixed-effects model (REML)	p = 0.1818	F (1, 95) = 1.809	ns	
**S5C**	OPEN-LA Total **Time spent** in NoSA (Scatter plot, total 15min)	Gr64f ACV+Sweet(ATR+) vs. Gr64f ACV+No taste(ATR-) 15 min	Mann-Whitney U test	p = 0.4224	U = 1078	ns	
		Gr66a BENZ+Bitter(ATR+) vs. Gr66a BENZ+No taste(ATR-) 15 min		p = 0.05	U = 948	ns	
		Gr64f ACV+No taste(ATR-) vs. Gr66a BENZ+No taste(ATR-) 15min	Mann-Whitney U test	p < 0.0001	U = 142	****	
		Gr64f ACV+Sweet(ATR+) vs. Gr66a BENZ+Bitter(ATR+) 15min		p < 0.0001	U = 126	****	
**S5D**	OPEN-LA Average **Distance moved** in NoSA (Line graph)	Gr64f ACV+Sweet(ATR+) vs. Gr64f ACV+No taste(ATR-) 15 min	Mixed-effects model (REML)	p = 0.9067	F (1, 92) = 0.01382	ns	
**S5E**	OPEN-LA Average **Distance moved** in NoSA (Line graph)	Gr66a BENZ+Bitter(ATR+) vs. Gr66a BENZ+No taste(ATR-) 15 min	Mixed-effects model (REML)	p = 0.5071	F (1, 95) = 0.4435	ns	
**S5F**	OPEN-LA Total **Distance moved** in NoSA (Scatter plot, total 15min)	Gr64f ACV+Sweet(ATR+) vs. Gr64f ACV+No taste(ATR-) 15 min	Mann-Whitney U test	p = 0.307	U = 1037	ns	
		Gr66a BENZ+Bitter(ATR+) vs. Gr66a BENZ+No taste(ATR-) 15 min		p = 0.4728	U = 1166	ns	
		Gr64f ACV+No taste(ATR-) vs. Gr66a BENZ+No taste(ATR-) 15min	Mann-Whitney U test	p < 0.0001	U = 72	****	
		Gr64f ACV+Sweet(ATR+) vs. Gr66a BENZ+Bitter(ATR+) 15min		p < 0.0001	U = 56	****	
**S5G**	OPEN-LA Average **Depth** in NoSA (Line graph)	Gr64f ACV+Sweet(ATR+) vs. Gr64f ACV+No taste(ATR-) 15 min	Mixed-effects model (REML)	p = 0.6155	F (1, 92) = 0.2540	ns	
**S5H**	OPEN-LA Average **Depth** in NoSA (Line graph)	Gr66a BENZ+Bitter(ATR+) vs. Gr66a BENZ+No taste(ATR-) 15 min	Mixed-effects model (REML)	p = 0.1976	F (1, 95) = 1.683	ns	
**S5I**	OPEN-LA Total **Depth** in NoSA (Scatter plot, total 15min)	Gr64f ACV+Sweet(ATR+) vs. Gr64f ACV+No taste(ATR-) 15 min	Mann-Whitney U test	p = 0.02	U = 822	*	
		Gr66a BENZ+Bitter(ATR+) vs. Gr66a BENZ+No taste(ATR-) 15 min		p = 0.03	U = 917	*	
		Gr64f ACV+No taste(ATR-) vs. Gr66a BENZ+No taste(ATR-) 15min	Mann-Whitney U test	p = 0.0110	U = 821	*	
		Gr64f ACV+Sweet(ATR+) vs. Gr66a BENZ+Bitter(ATR+) 15min		p = 0.0007	U = 720	***	
**S5J**	OPEN-LA **Bout duration** in NoSA (Scatter plot, total 15min)	Gr64f ACV+Sweet(ATR+) vs. Gr64f ACV+No taste(ATR-) 15 min	Mann-Whitney U test	p = 0.0746	U = 913	ns	
		Gr66a BENZ+Bitter(ATR+) vs. Gr66a BENZ+No taste(ATR-) 15 min		p = 0.03	U = 917	*	
		Gr64f ACV+No taste(ATR-) vs. Gr66a BENZ+No taste(ATR-) 15min	Mann-Whitney U test	p = 0.1979	U = 1013	ns	
		Gr64f ACV+Sweet(ATR+) vs. Gr66a BENZ+Bitter(ATR+) 15min		p = 0.173	U = 1022	ns	
**S5K**	OPEN-LA **Bout distance** in NoSA (Scatter plot, total 15min)	Gr64f ACV+Sweet(ATR+) vs. Gr64f ACV+No taste(ATR-) 15 min	Mann-Whitney U test	p = 0.0287	U = 853	*	
		Gr66a BENZ+Bitter(ATR+) vs. Gr66a BENZ+No taste(ATR-) 15 min		p = 0.0096	U = 852	***	
		Gr64f ACV+No taste(ATR-) vs. Gr66a BENZ+No taste(ATR-) 15min	Mann-Whitney U test	p = 0.03	U = 875	*	
		Gr64f ACV+Sweet(ATR+) vs. Gr66a BENZ+Bitter(ATR+) 15min		p = 0.01	U = 839	**	
**S6A**	OPEN-LA Average **Time spent** in ROSA (Line graph)	Gr64f ACV+Sweet(ATR+) females vs. males 15 min	Mixed-effects model (REML)	p = 0.0396	F (1, 45) = 4.493	*	
**S6B**	OPEN-LA Average **Time spent** in NoSA (Line graph)	Gr64f ACV+Sweet(ATR+) females vs. males 15 min		p = 0.3086	F (1, 45) = 1.060	ns	
**S6C**	OPEN-LA Average **Distance moved** in ROSA (Line graph)	Gr64f ACV+Sweet(ATR+) females vs. males 15 min		p = 0.1022	F (1, 45) = 2.782	ns	
**S6D**	OPEN-LA Average **Distance moved** in NoSA (Line graph)	Gr64f ACV+Sweet(ATR+) females vs. males 15 min		p < 0.0001	F (1, 45) = 35.50	****	
**S6E**	OPEN-LA Average **Depth** in ROSA (Line graph)	Gr64f ACV+Sweet(ATR+) females vs. males 15 min		p = 0.4396	F (1, 45) = 0.6082	ns	

**S6F**	OPEN-LA Average **Depth** in NoSA (Line graph)	Gr64f ACV+Sweet(ATR+) females vs. males 15 min		p = 0.9826	F (1, 45) = 0.0004800	ns	
**S6G**	OPEN-LA **Bout duration** in ROSA (Scatter plot)	Gr64f ACV+Sweet(ATR+) females vs. males 15 min		p = 0.6047	U = 250	ns	Gr64f ACV+Sweet(ATR+) females n = 25, males n = 22
	OPEN-LA **Bout duration** in NoSA (Scatter plot)	Gr64f ACV+Sweet(ATR+) females vs. males 15 min		p = 0.2225	U = 217	ns	
**S6H**	OPEN-LA **Bout distance** in ROSA (Scatter plot)	Gr64f ACV+Sweet(ATR+) females vs. males 15 min		p = 0.7918	U = 262	ns	
	OPEN-LA **Bout distance** in NoSA (Scatter plot)	Gr64f ACV+Sweet(ATR+) females vs. males 15 min	Mann-Whitney U test	p = 0.79	U = 262	ns	
**S6I**	OPEN-LA **Latency to re-enter** ROSA (Scatter plot)	Gr64f ACV+Sweet(ATR+) females vs. males 15 min		p = 0.6047	U = 250	ns	
**S6J**	OPEN-LA **<2 sec odor+taste sampling** in ROSA (Scatter plot)	Gr64f ACV+Sweet(ATR+) females vs. males 15 min		p = 0.02	U = 82.5	*	
**S6K**	OPEN-LA **total entries** into ROSA (Scatter plot)	Gr64f ACV+Sweet(ATR+) females vs. males 15 min		p = 0.3997	U = 235	ns	
**S6L**	OPEN-LA Average **Time spent** in ROSA (Line graph)	Gr66a BENZ+Bitter(ATR+) females vs. males 15 min		p = 0.0098	F (1, 47) = 7.239	**	
**S6M**	OPEN-LA Average **Time spent** in NoSA (Line graph)	Gr66a BENZ+Bitter(ATR+) females vs. males 15 min		p = 0.4413	F (1, 47) = 0.6031	ns	
**S6N**	OPEN-LA Average **Distance moved** in ROSA (Line graph)	Gr66a BENZ+Bitter(ATR+) females vs. males 15 min	Mixed-effects model (REML)	p = 0.0161	F (1, 47) = 6.229	*	
**S6O**	OPEN-LA Average **Distance moved** in NoSA (Line graph)	Gr66a BENZ+Bitter(ATR+) females vs. males 15 min		p = 0.2227	F (1, 47) = 1.527	ns	
**S6P**	OPEN-LA Average **Depth** in ROSA (Line graph)	Gr66a BENZ+Bitter(ATR+) females vs. males 15 min		p = 0.0156	F (1, 47) = 6.299	*	
**S6Q**	OPEN-LA Average **Depth** in NoSA (Line graph)	Gr66a BENZ+Bitter(ATR+) females vs. males 15 min		p = 0.7527	F (1, 47) = 0.1004	ns	
**S6R**	OPEN-LA **Bout duration** in ROSA (Scatter plot)	Gr66a BENZ+Bitter(ATR+) females vs. males 15 min		p = 0.1638	U = 227	ns	Gr66a BENZ+Bitter(ATR+) females n = 22, males n = 27
	OPEN-LA **Bout duration** in NoSA (Scatter plot)	Gr66a BENZ+Bitter(ATR+) females vs. males 15 min		p = 0.5846	U = 269	ns	
**S6S**	OPEN-LA **Bout distance** in ROSA (Scatter plot)	Gr66a BENZ+Bitter(ATR+) females vs. males 15 min		p = 0.1352	U = 222	ns	
	OPEN-LA **Bout distance** in NoSA (Scatter plot)	Gr66a BENZ+Bitter(ATR+) females vs. males 15 min	Mann-Whitney U test	p = 0.57	U = 268	ns	
**S6T**	OPEN-LA **Latency to re-enter** ROSA (Scatter plot)	Gr66a BENZ+Bitter(ATR+) females vs. males 15 min		p = 0.0411	U = 171	ns	
**S6U**	OPEN-LA **<2 sec odor+taste sampling** in ROSA (Scatter plot)	Gr66a BENZ+Bitter(ATR+) females vs. males 15 min		p = 0.0467	t(1,40) = 2.05	*	
**S6V**	OPEN-LA **total entries** into ROSA (Scatter plot)	Gr66a BENZ+Bitter(ATR+) females vs. males 15 min		p = 0.4765	t(1,40) = 0.7177	ns	

p>0.05 non-significant (ns), p<0.05*, p<0.01, p<0.001***, p<0.0001******

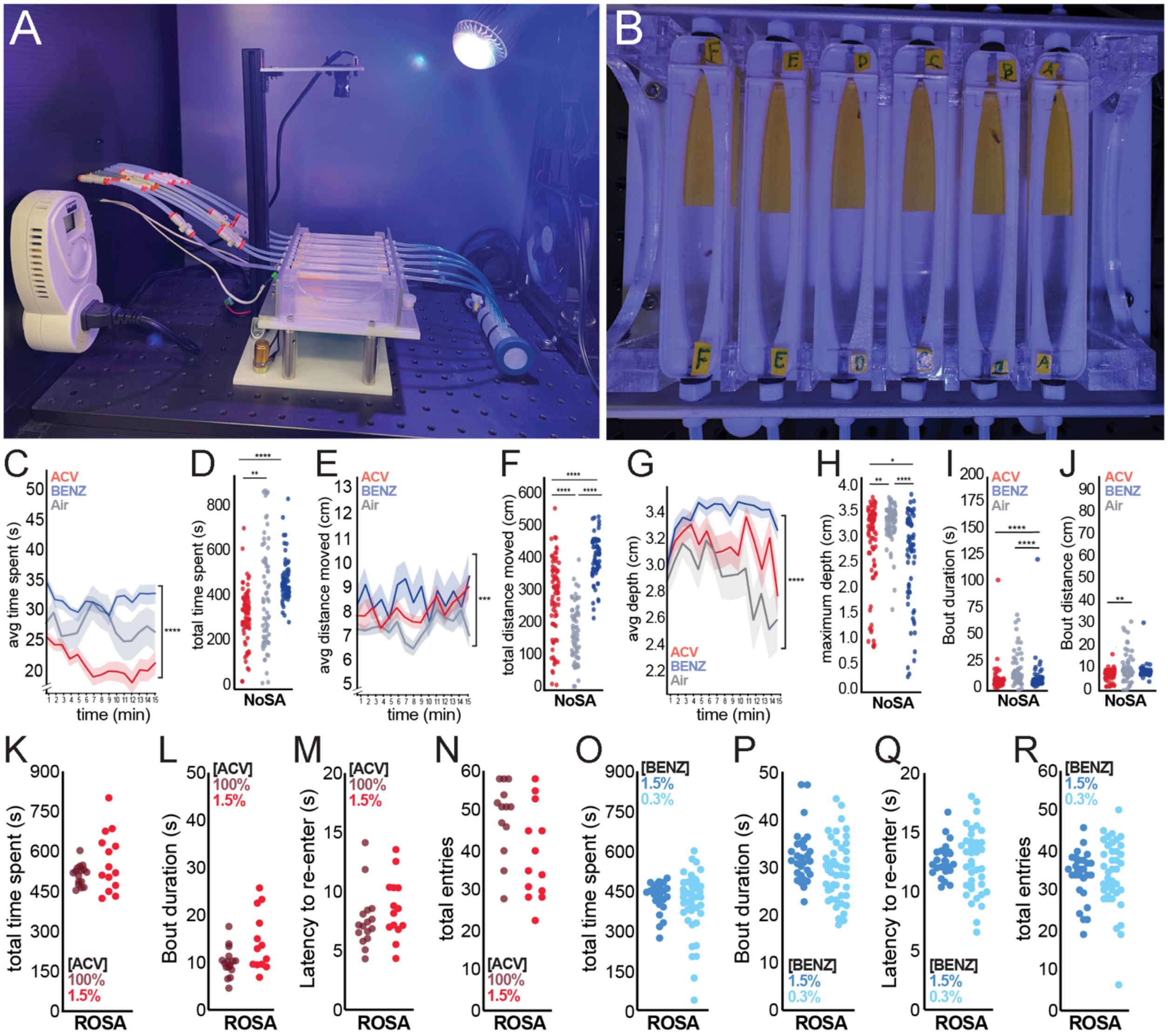

Fig. S1. **Apparatus, behaviors in NoSA upon exposure to appetitive and aversive odors, behaviors in ROSA upon exposure to varying odor concentrations. (A-B)** Pictures of OPEN-LA arenas and apparatus. **(A)** OPEN-LA arenas reside in a temperature, light, and humidity-controlled environment. **(B)** Top view of the six OPEN-LA arenas. **(C-J)** Operant behaviors in NoSA for flies experiencing appetitive and aversive odors alone. Behavioral metrics in NoSA are shown for *Canton-S* flies experiencing apple cider vinegar (ACV, n = 76), humidified Air (n = 65), and benzaldehyde (BENZ, n = 74). **(C)** Line graph depicting the average time spent (seconds) in ROSA across 15 minutes. **(D)** Scatter plot depicting total time spent in ROSA across 15 minutes. Each data point represents the total time spent (sum of time spent across the 15 minutes) for each fly. **(E)** Line graph depicting the average distance moved (centimeters) in ROSA across 15 minutes. **(F)** Scatter plot depicting total distance moved in ROSA across 15 minutes. Each data point represents the total distance moved (sum of distance moved across the 15 minutes) for each fly. **(G)** Line graph depicting the average depth (centimeters) towards the ROSA port across 15 minutes for appetitive stimuli. **(H)** Scatter plot depicting maximum depth towards the ROSA port across 15 minutes for appetitive and aversive stimuli. Each data point represents the maximum depth achieved (average depth across the 15 minutes) by each fly. **(I)** Scatter plot depicting bout duration (seconds) and **(J)** bout distance (centimeters) within NoSA for appetitive and aversive experiences. For line graphs, data are mean ± SEM, and for scatter plots, each data point represents a single fly. **(K-R)**

Behavioral metrics in ROSA are shown for *Canton-S* flies experiencing 100% ACV (n = 16), 1.5% ACV (n = 14), 1.5% BENZ (n = 29), and 0.3% BENZ (n = 45). Scatter plots depicting total time spent **(K,O)**, bout duration **(L-P)**, latency to re-enter **(M-Q),** and total entries **(N-R)** in ROSA for flies experiencing appetitive and aversive odors alone at varying concentrations. Asterisks indicate statistically significant differences, *p < 0.05, **p < 0.01, ***p < 0.001, ****p < 0.0001. Detailed statistics are presented in Table S1.

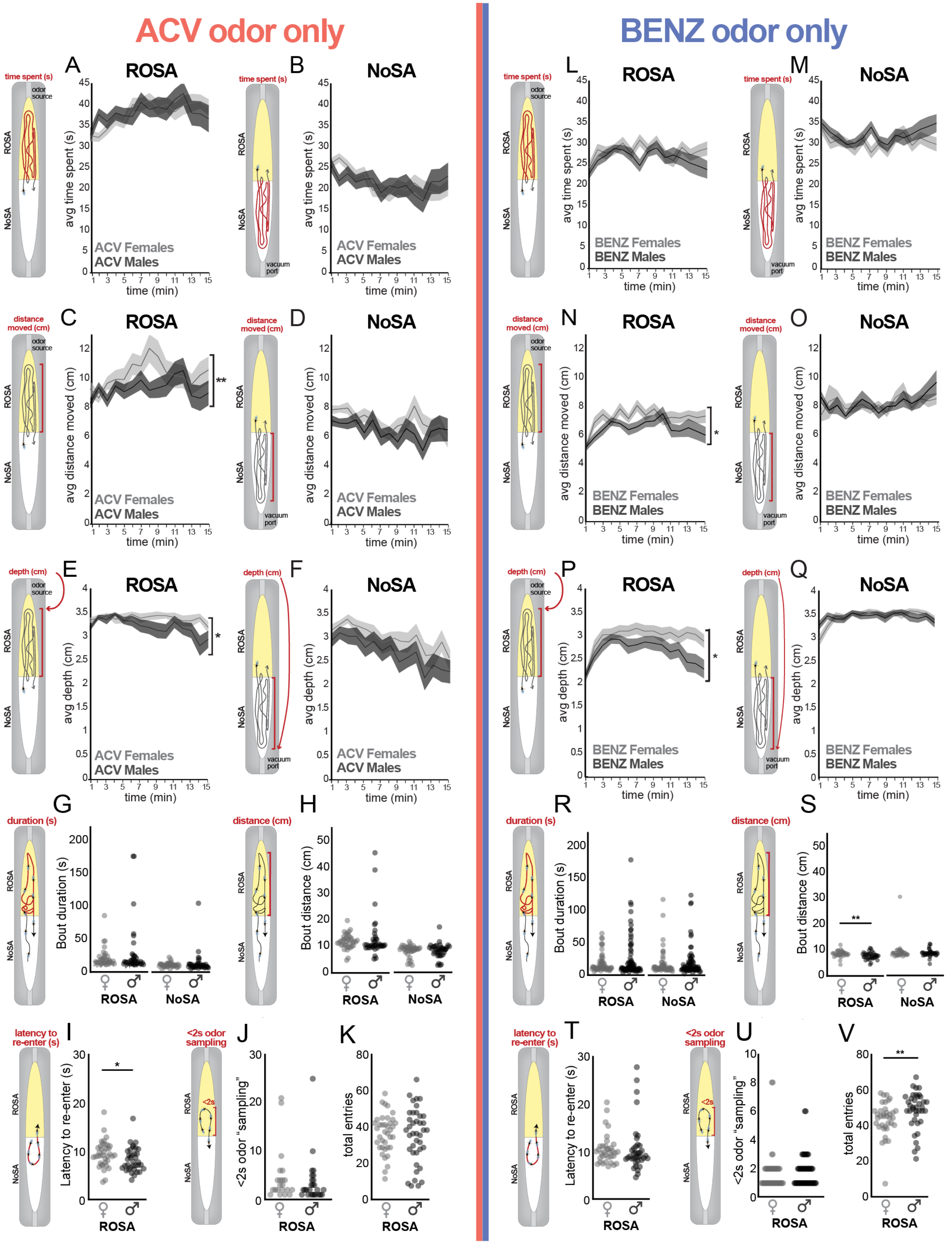

Journal of Experimental Biology • Supplementary information

Fig. S2. Operant behaviors in ROSA and NoSA for female vs. male flies experiencing the appetitive or aversive odor alone. (A-K) Behavioral metrics in ROSA and NoSA are shown for *Canton-S* female (n = 38) and male (n = 39) flies experiencing Apple cider vinegar (ACV) odor. **(A-B)** Line graphs depicting the average time spent (seconds) in **(A)** ROSA and **(B)** NoSA across 15 minutes. **(C-D)** Line graphs depicting the average distance moved (centimeters) in **(C)** ROSA and **(D)** NoSA across 15 minutes. **(E-F)** Line graphs depicting the average depth towards the **(E)** ROSA air/odor port and **(F)** NoSA vacuum port across 15 minutes. For line graphs, data are mean ± SEM, and comparisons were conducted using Two-way ANOVA. **(G-H)** Scatter plots depicting **(G)** bout duration (seconds) and **(H)** bout distance (centimeters) within ROSA and NoSA for each sex. **(I-K)** Scatter plots depicting **(I)** latency to re-enter the ROSA (seconds), **(J)** number of <2-second sampling events in ROSA, and **(K)** total entries into ROSA for each sex. For scatter plots, each data point represents a fly. **(L-V)** Behavioral metrics in ROSA and NoSA are shown for *Canton-S* female (n = 36) and male (n = 38) flies experiencing benzaldehyde (BENZ) odor. **(L-M)** Line graphs depicting the average time spent (seconds) in **(L)** ROSA and **(M)** NoSA across 15 minutes. **(N-O)** Line graphs depicting the average distance moved (centimeters) in **(N)** ROSA and **(O)** NoSA across 15 minutes. **(P-Q)** Line graphs depicting the average depth towards the **(P)** ROSA air/odor port and **(Q)** NoSA vacuum port across 15 minutes. For line graphs, data are mean ± SEM, and comparisons were conducted using Two-way ANOVA. **(R-S)** Scatter plots depicting **(R)** bout duration (seconds) and **(S)** bout distance (centimeters) within ROSA and NoSA for each sex. **(T-V)** Scatter plots depicting **(T)** latency to re-enter the ROSA (seconds), **(U)** number of <2-second sampling events in ROSA, and **(V)** total entries into ROSA for each sex. For scatter plots, each data point represents a fly. Asterisks indicate statistically significant differences, *p < 0.05, **p < 0.01, ***p < 0.001, ****p < 0.0001. Detailed statistics are presented in Table S1.

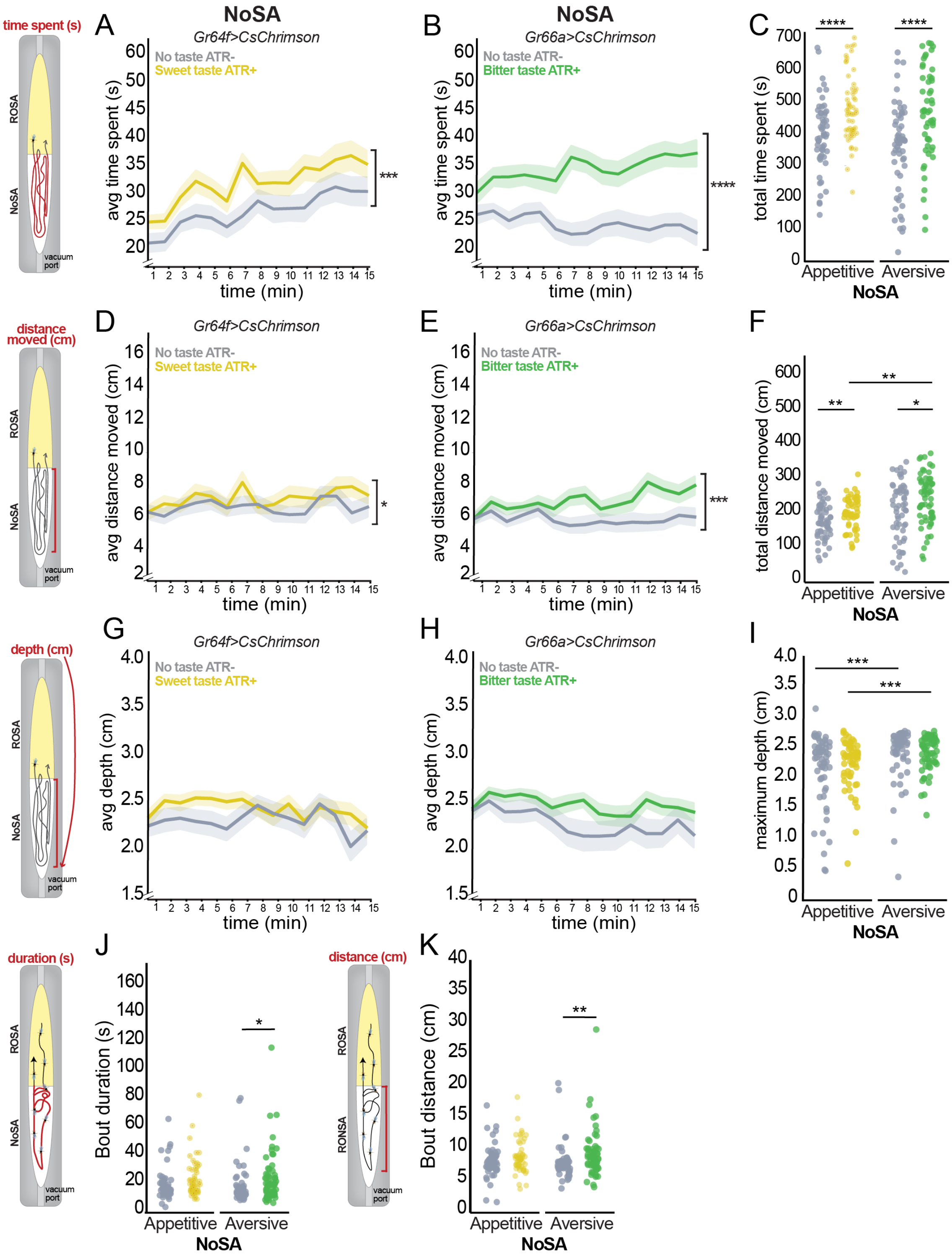

Fig. S3. Operant behaviors in NoSA for flies experiencing appetitive and aversive taste activation. Behavioral metrics in NoSA are shown for *Gr64f>CsChrimson* No taste controls (ATR-) (n = 56), *Gr64f>CsChrimson* Sweet Taste (ATR+) (n = 58), *Gr66a>CsChrimson* No taste controls (ATR-) (n = 57) and *Gr66a>CsChrimson* Bitter Taste (ATR+) (n = 61) flies. **(A)** Line graph depicting the average time spent (seconds) in ROSA across 15 minutes for appetitive taste. **(B)** Line graph depicting the average time spent in ROSA across 15 minutes for aversive taste. **(C)** Scatter plot depicting total time

Journal of Experimental Biology • Supplementary information

spent in ROSA across 15 minutes for appetitive and aversive tastes. Each data point represents the total time spent (sum of time spent across 15 minutes) for each fly. **(D)** Line graph depicting the average distance moved (centimeters) in ROSA across 15 minutes for appetitive taste. **(E)** Line graph depicting the average distance moved in ROSA across 15 minutes for aversive taste. **(F)** Scatter plot depicting total distance moved in ROSA across 15 minutes for appetitive and aversive tastes. Each data point represents the total distance moved (sum of distance moved across 15 minutes) for each fly. **(G)** Line graph depicting the average depth (centimeters) towards the ROSA port across 15 minutes for appetitive taste. **(H)** Line graph depicting the average depth towards the ROSA port across 15 minutes for aversive taste. **(I)** Scatter plot depicting maximum depth towards the ROSA port across 15 minutes for appetitive and aversive tastes. Each data point represents the maximum depth achieved (average depth across 15 minutes) by each fly. **(J)** Scatter plot depicting bout duration (seconds) and **(K)** bout distance (centimeters) within NoSA for appetitive and aversive experiences. For line graphs, data are mean ± SEM, and for scatter plots, each data point represents a single fly. Asterisks indicate statistically significant differences, *p < 0.05, **p < 0.01, ***p < 0.001, ****p < 0.0001. Detailed statistics are presented in Table S1.

Journal of Experimental Biology • Supplementary information

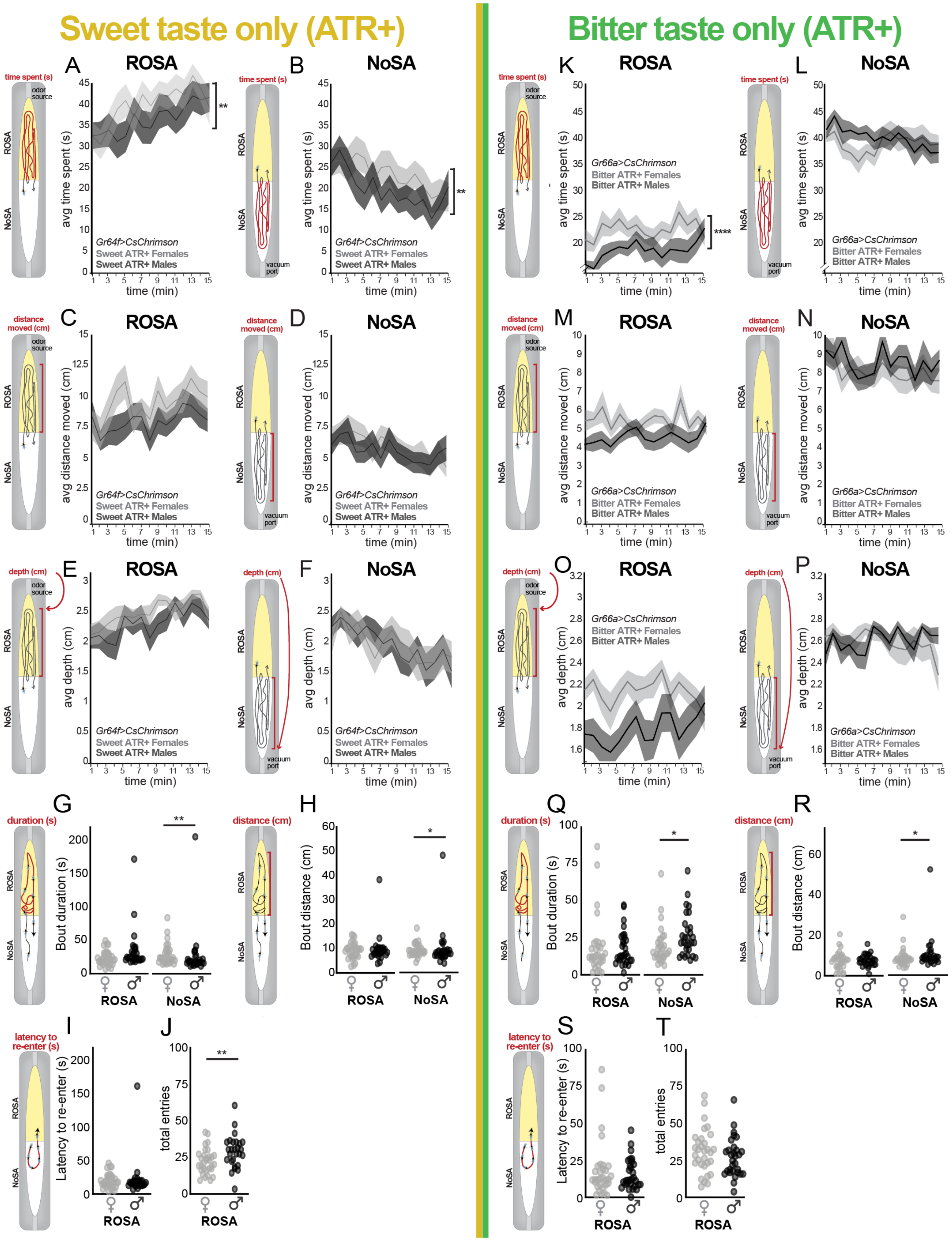

Journal of Experimental Biology • Supplementary information

Fig. S4. Operant behaviors in ROSA and NoSA for female vs. male flies experiencing the appetitive or aversive taste activation alone. (A-J) Behavioral metrics in ROSA and NoSA are shown for *Gr64f>CsChrimson* female (n = 25) and male (n = 22) flies experiencing Sweet taste (ATR+) optogenetic activation. **(A-B)** Line graphs depicting the average time spent (seconds) in **(A)** ROSA and **(B)** NoSA across 15 minutes. **(C-D)** Line graphs depicting the average distance moved (centimeters) in **(C)** ROSA and **(D)** NoSA across 15 minutes. **(E-F)** Line graphs depicting the average depth towards the **(E)** ROSA air port and **(F)** NoSA vacuum port across 15 minutes. For line graphs, data are mean ± SEM. **(G-H)** Scatter plots depicting **(G)** bout duration (seconds) and **(H)** bout distance (centimeters) within ROSA and NoSA for each sex. **(I-J)** Scatter plots depicting **(I)** latency to re-enter the ROSA (seconds), and **(J)** total entries into ROSA for each sex. **(K-T)** Behavioral metrics in ROSA and NoSA are shown for *Gr66a>CsChrimson* female (n = 22) and male (n = 27) flies experiencing Bitter taste (ATR+) optogenetic activation. **(K-L)** Line graphs depicting the average time spent (seconds) in **(K)** ROSA and **(L)** NOSA across 15 minutes. **(M-N)** Line graphs depicting the average distance moved (centimeters) in **(M)** ROSA and **(N)** NoSA across 15 minutes. **(O-P)** Line graphs depicting the average depth towards the **(O)** ROSA air port and **(P)** NoSA vacuum port across 15 minutes. For line graphs, data are mean ± SEM. **(Q-R)** Scatter plots depicting **(Q)** bout duration (seconds) and **(R)** bout distance (centimeters) within ROSA and NoSA for each sex. **(S-T)** Scatter plots depicting **(S)** latency to re-enter the ROSA (seconds), and **(T)** total entries into ROSA for each sex. For scatter plots, each data point represents a fly. Asterisks indicate statistically significant differences, *$p < 0.05$, **$p < 0.01$, ***$p < 0.001$, ****$p < 0.0001$. Detailed statistics are presented in Table S1.

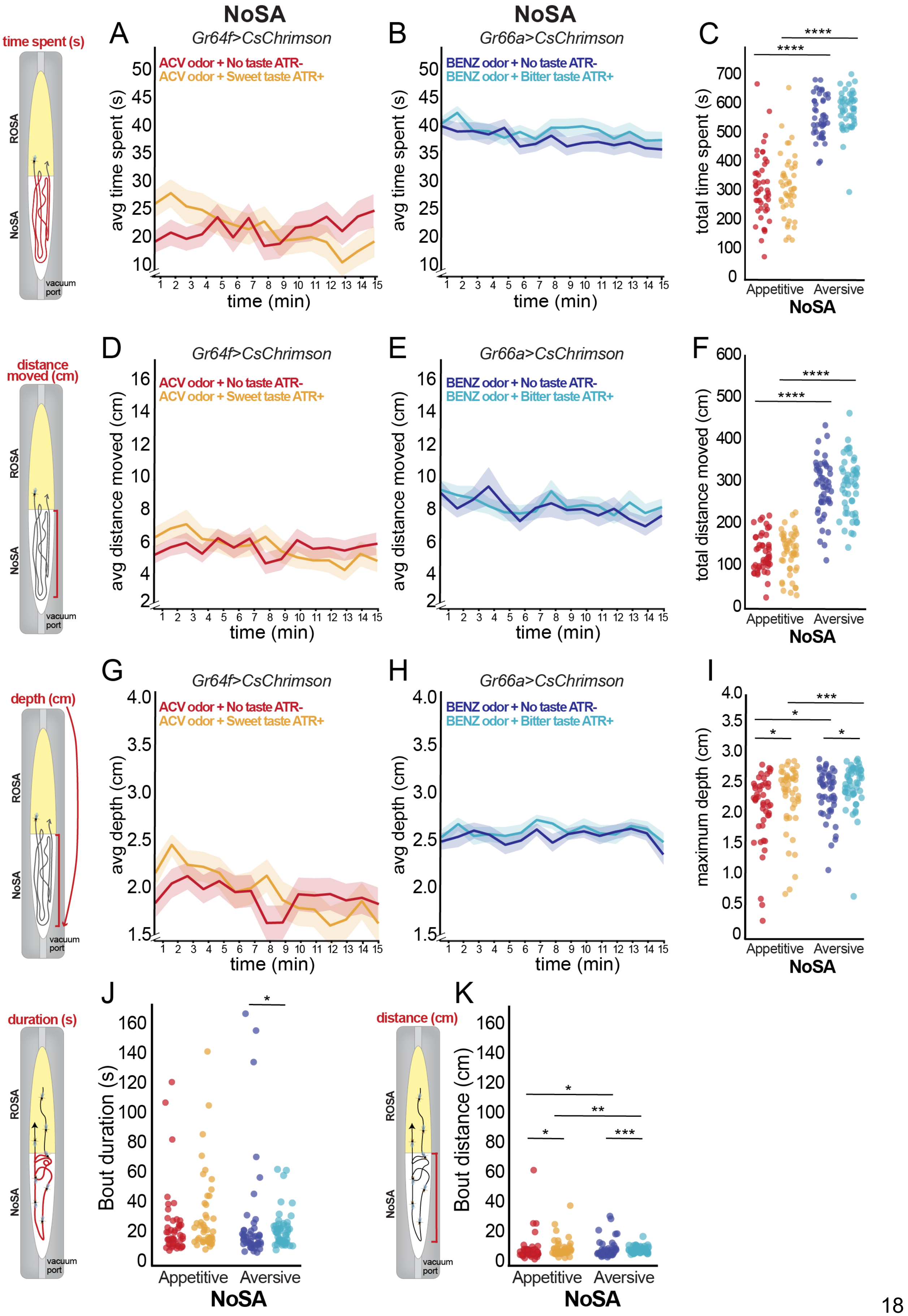

Journal of Experimental Biology • Supplementary information

Fig. S5. Operant behaviors in NoSA for flies experiencing multisensory appetitive and aversive outcomes. Behavioral metrics in NoSA are shown for *Gr64f>CsChrimson* flies experiencing ACV-only (ATR-) (n = 47) and ACV+Sweet (ATR+) (n = 47), as well as *Gr66a>CsChrimson* flies experiencing BENZ-only (ATR-) (n = 49) and BENZ+Bitter (ATR+) (n = 48). **(A)** Line graph depicting average time spent (seconds) in ROSA across 15 minutes for appetitive stimuli. **(B)** Line graph depicting average time spent in ROSA across 15 minutes for aversive stimuli. **(C)** Scatter plot depicting total time spent in ROSA across 15 minutes for appetitive and aversive stimuli. Each data point represents the total time spent (sum of time spent across the 15 minutes) for each fly. **(D)** Line graph depicting the average distance moved (centimeters) in ROSA across 15 minutes for appetitive stimuli. **(E)** Line graph depicting the average distance moved in ROSA across 15 minutes for aversive stimuli. **(F)** Scatter plot depicting total distance moved in ROSA across 15 minutes for appetitive and aversive stimuli. Each data point represents the total distance moved (sum of distance moved across the 15 minutes) for each fly. **(G)** Line graph depicting the average depth (centimeters) towards the ROSA port across 15 minutes for appetitive stimuli. **(H)** Line graph depicting the average depth towards the ROSA port across 15 minutes for aversive stimuli. **(I)** Scatter plot depicting maximum depth towards the ROSA port across 15 minutes for appetitive and aversive stimuli. Each data point represents the maximum depth achieved (average depth across the 15 minutes) by each fly. **(J)** Scatter plot depicting bout duration (seconds) and **(K)** bout distance (centimeters) within for appetitive and aversive experiences. For line graphs, data are mean ± SEM, and for scatter plots depicting averages, each data point is the mean for each fly. Asterisks indicate statistically significant differences, $*p < 0.05$, $**p < 0.01$, $***p < 0.001$, $****p < 0.0001$. Detailed statistics are presented in Table S1.

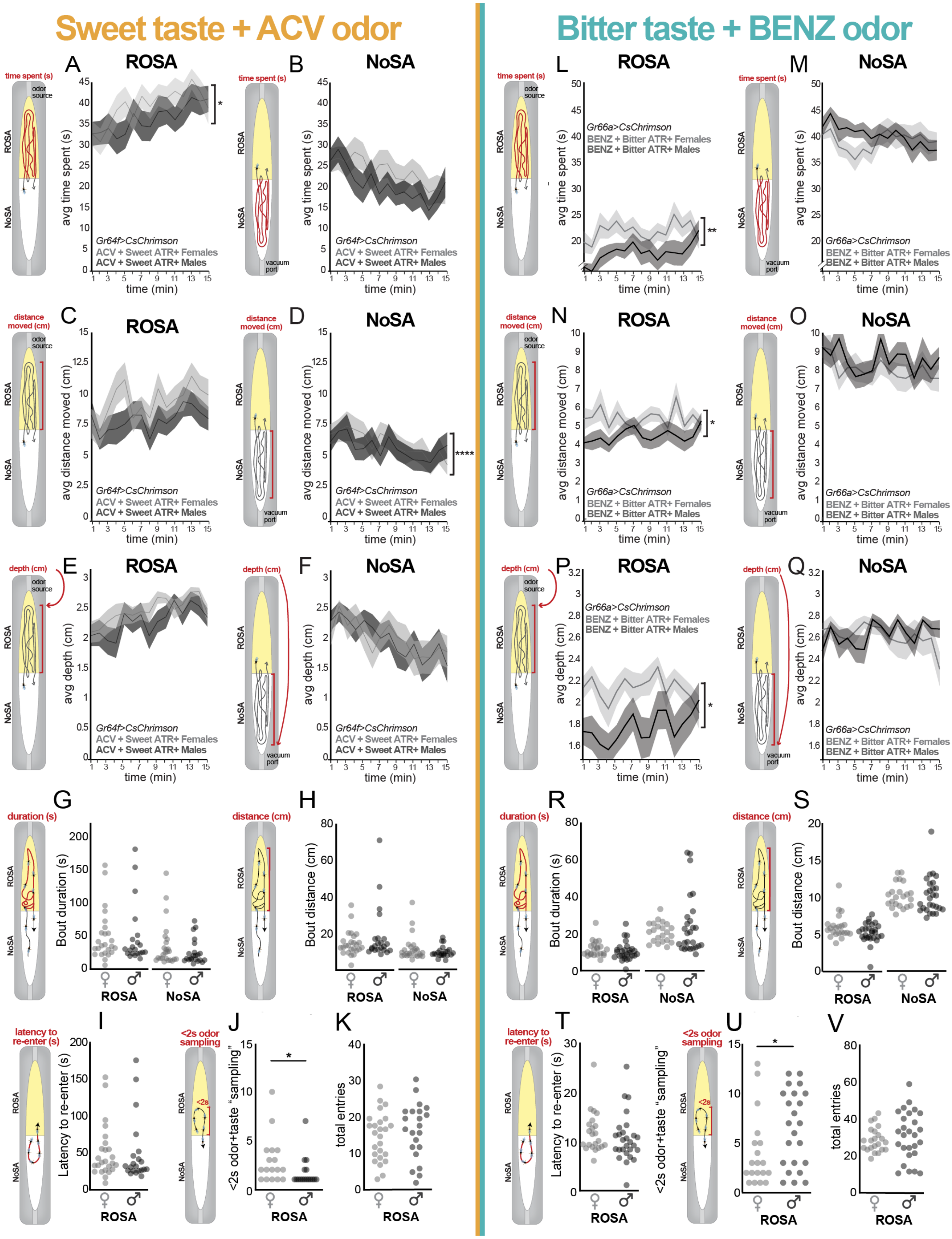

Journal of Experimental Biology • Supplementary information

Fig. S6. Operant behaviors in ROSA and NoSA for female vs. male flies experiencing multisensory appetitive or aversive stimuli. (A-K) Behavioral metrics in ROSA and NoSA are shown for *Gr64f>CsChrimson* female (n = 25) and male (n = 22) flies experiencing apple cider vinegar (ACV) odor and Sweet taste (ATR+). **(A-B)** Line graphs depicting the average time spent (seconds) in **(A)** ROSA and **(B)** NoSA across 15 minutes. **(C-D)** Line graphs depicting the average distance moved (centimeters) in **(C)** ROSA and **(D)** NoSA across 15 minutes. **(E-F)** Line graphs depicting the average depth towards the **(E)** ROSA odor port and **(F)** NoSA vacuum port across 15 minutes. For line graphs, data are mean ± SEM. **(G-H)** Scatter plots depicting **(G)** bout duration (seconds) and **(H)** bout distance (centimeters) within ROSA and NoSA for each sex. **(I-K)** Scatter plots depicting **(I)** latency to re-enter the ROSA (seconds), **(J)** number of <2-second sampling events in ROSA, and **(K)** total entries into ROSA for each sex. For scatter plots, each data point represents a fly. **(L-V)** Behavioral metrics in ROSA and NoSA are shown for *Gr66a>CsChrimson* female (n = 22) and male (n = 27) flies experiencing benzaldehyde (BENZ) odor and Bitter taste (ATR+). **(L-M)** Line graphs depicting the average time spent (seconds) in **(L)** ROSA and **(M)** NoSA across 15 minutes. **(N-O)** Line graphs depicting the average distance moved (centimeters) in **(N)** ROSA and **(O)** NoSA across 15 minutes. **(P-Q)** Line graphs depicting the average depth towards the **(P)** ROSA odor port and **(Q)** NoSA vacuum port across 15 minutes. For line graphs, data are mean ± SEM. **(R-S)** Scatter plots depicting **(R)** bout duration (seconds) and **(S)** bout distance (centimeters) within ROSA and NoSA for each sex. **(T-V)** Scatter plots depicting **(T)** latency to re-enter the ROSA (seconds), **(U)** number of <2-second sampling events in ROSA, and **(V)** total entries into ROSA for each sex. For scatter plots, each data point represents a fly. Asterisks indicate statistically significant differences, *p < 0.05, **p < 0.01. Detailed statistics are presented in Table S1.

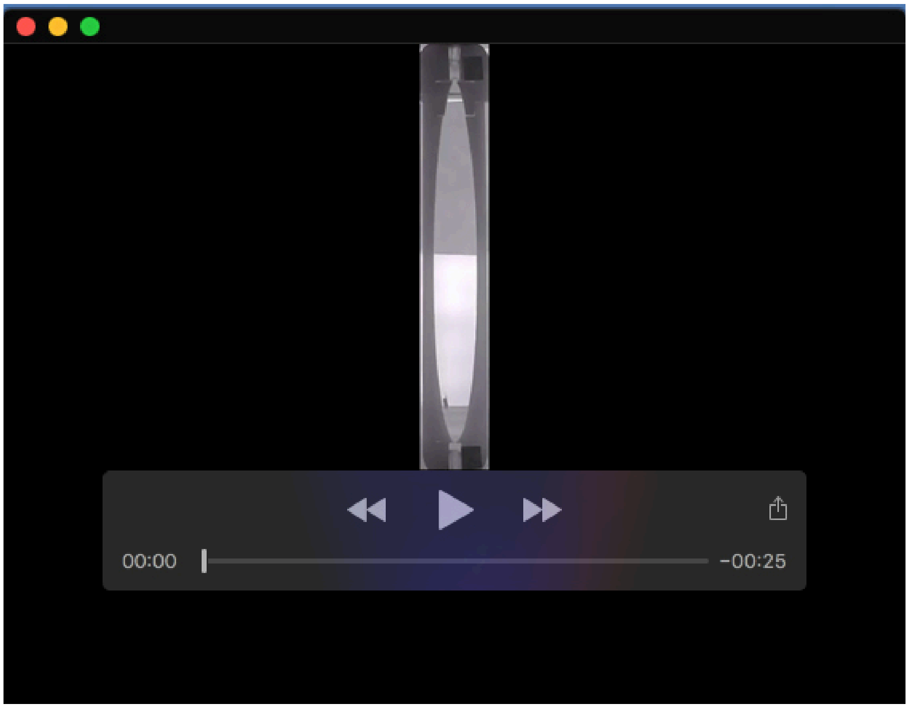

Movie 1. Representative video showing behavioral tracking of *Canton-S* flies in response to ACV. Full 15-minute video showing fly behavior in the OpenLA arena.

Journal of Experimental Biology • Supplementary information

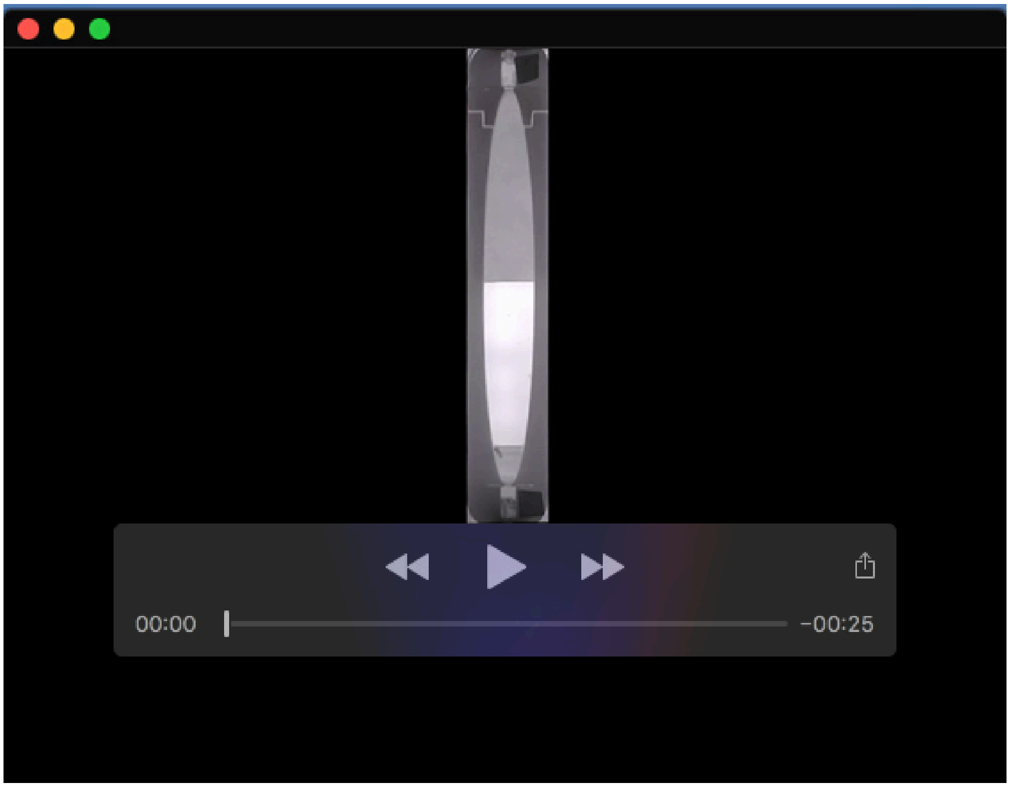

Movie 2. Representative video showing behavioral tracking of *Canton-S* flies in response to Air. Full 15-minute video showing fly behavior in the OpenLA arena.

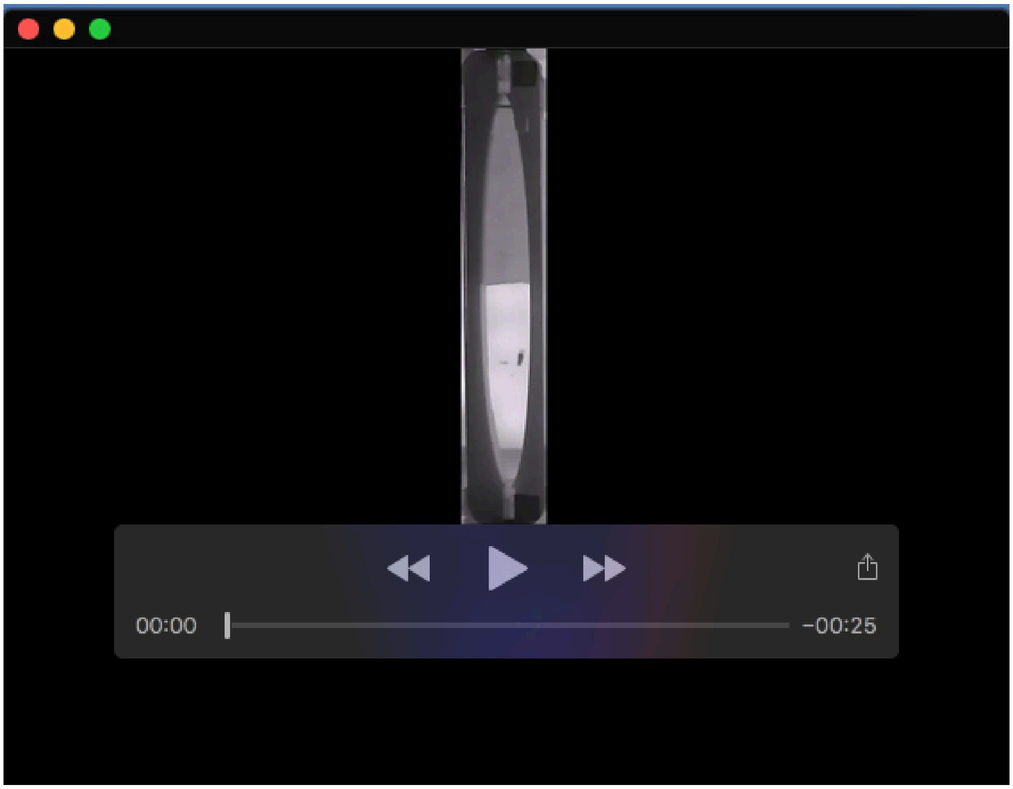

Movie 3. Representative video showing behavioral tracking of *Canton-S* flies in response to BENZ. Full 15-minute video showing fly behavior in the OpenLA arena.

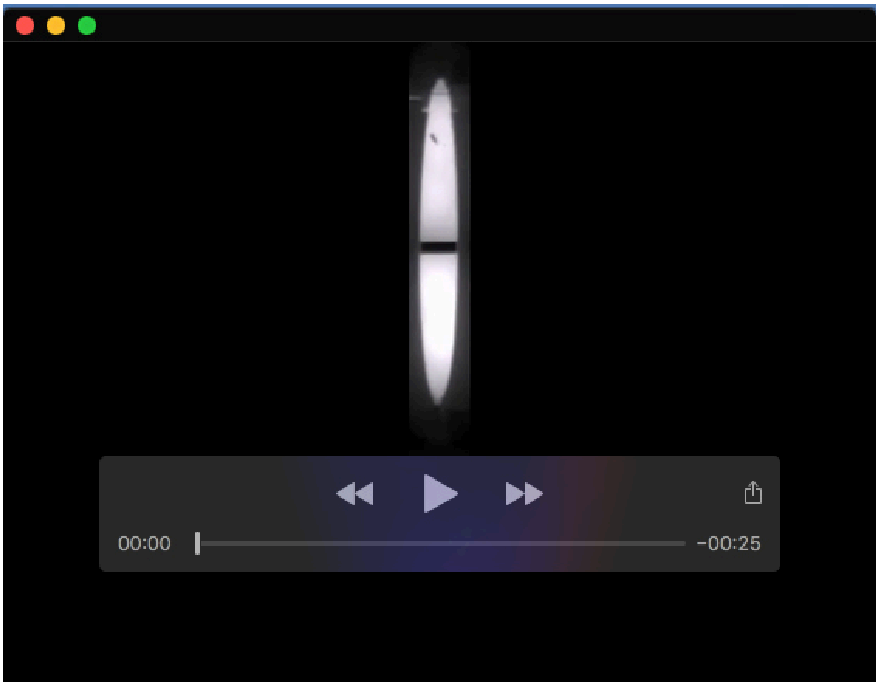

Movie 4. Representative video showing behavioral tracking in response to optogenetic activation of sweet taste (*Gr64f>CsChrimson* ATR+ flies). Full 15-minute video showing fly behavior in the OpenLA arena. Note that the decreased resolution in optogenetic videos is a result of the lower light levels used in the apparatus to minimize the unwanted activation of *Gr>Chrimson.*

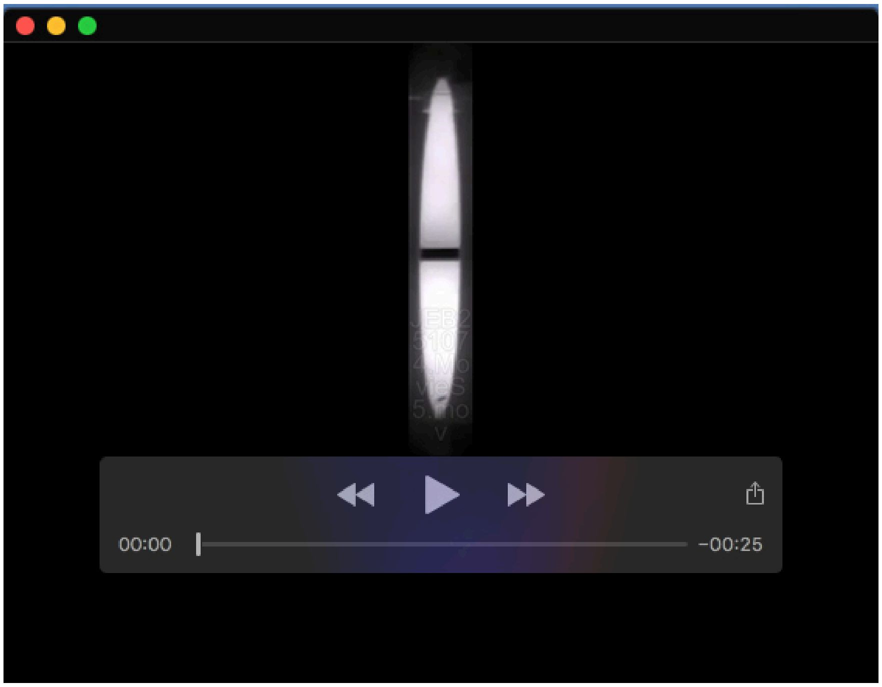

Movie 5. Representative video showing behavioral tracking in response to optogenetic activation of bitter taste (*Gr66a>CsChrimson* ATR+ flies). Full 15-minute video showing fly behavior in the OpenLA arena. Note that the decreased resolution in optogenetic videos is a result of the lower light levels used in the apparatus to minimize the unwanted activation of *Gr>Chrimson.*

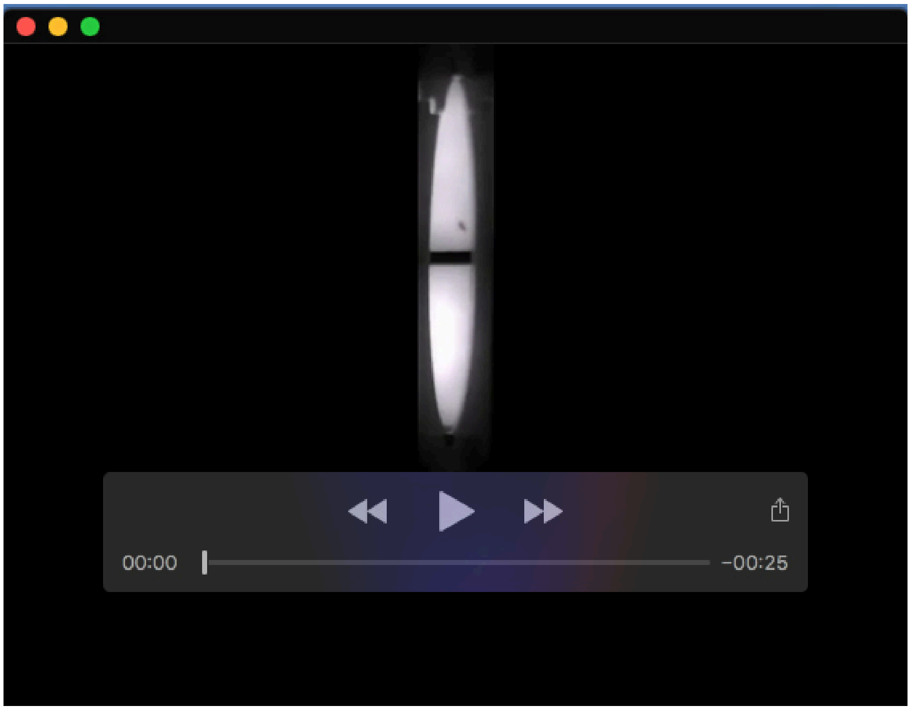

Movie 6. Representative video showing behavioral tracking in response to ACV exposure concurrent with optogenetic activation of sweet taste (*Gr64f>CsChrimson* ATR+ flies). Full 15-minute video showing fly behavior in the OpenLA arena. Note that the decreased resolution in optogenetic videos is a result of the lower light levels used in the apparatus to minimize the unwanted activation of *Gr>Chrimson.*

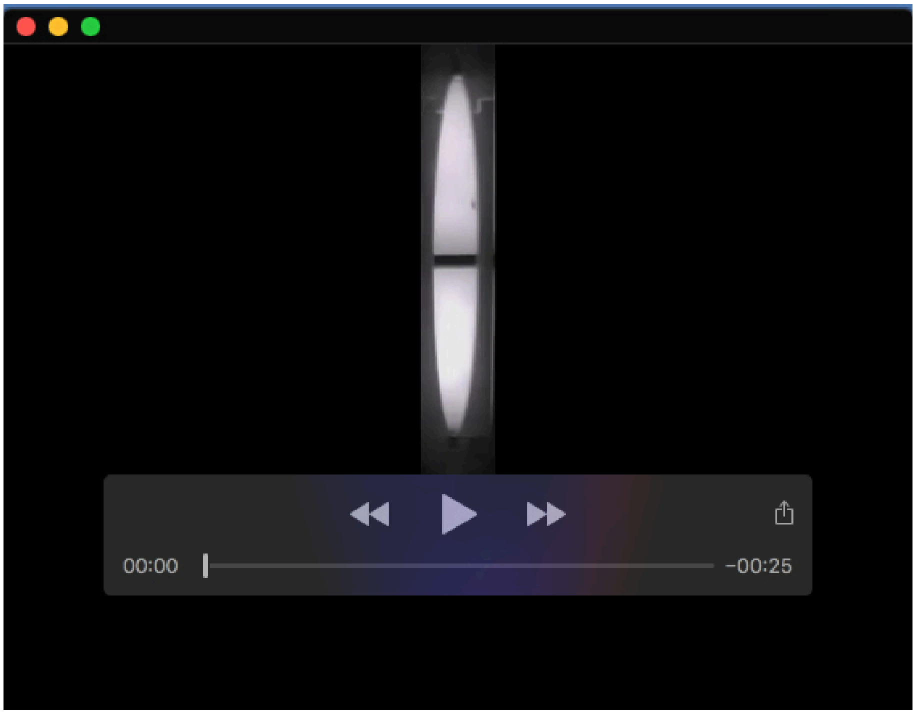

Movie 7. Representative video showing behavioral tracking in response to BENZ exposure concurrent with optogenetic activation of bitter taste (*Gr66a>CsChrimson* ATR+ flies). Full 15-minute video showing fly behavior in the OpenLA arena. Note that the decreased resolution in optogenetic videos is a result of the lower light levels used in the apparatus to minimize the unwanted activation of *Gr>Chrimson.*

Journal of Experimental Biology • Supplementary information
